# Diagnosis and Management of Infectious Encephalitis in Adults: Guidelines From the European Academy of Neurology and European Society of Clinical Microbiology and Infectious Diseases

**DOI:** 10.1111/ene.70647

**Published:** 2026-10-09

**Authors:** Julia Granerod, Johann Sellner, Jean‐Paul Stahl, Sylviane Defres, Alexandra Mailles, Erich Schmutzhard, Elisabeth Aurelius, Thomas Whitfield, Luca Massacesi, Maarten J. Titulaer, Pille Taba, Benedict D. Michael, Tehmina Bharucha, Lance Turtle, Brendan Sargent, Arina Tamborska, Jonathan Cattrall, Yun Huang, Eva Maria Hodel, Suzannah Lant, Glynn Webb, Rajish S. K. Shil, Shalley Dhar, Diederik van de Beek, Marie Studahl, Matthijs C. Brouwer, Uta Meyding‐Lamade, Ava Easton, Bhagteshwar Singh, Tom Solomon

**Affiliations:** ^1^ Department of Clinical Infection, Microbiology & Immunology, Institute of Infection, Veterinary and Ecological Sciences University of Liverpool Liverpool UK; ^2^ Dr JGW Consulting AS Sandefjord Norway; ^3^ Department of Neurology Landesklinikum Mistelbach Mistelbach‐Gänserndorf Austria; ^4^ Karl Landsteiner University of Health Sciences Krems Austria; ^5^ Infectious Diseases Grenoble Alps University Grenoble France; ^6^ ESCMID Study Group on Infections of the Brain (ESGIB) Basel Switzerland; ^7^ Tropical and Infectious Diseases Unit Liverpool University Hospitals NHS Foundation Trust Liverpool UK; ^8^ Faculty of Education Liverpool School of Tropical Medicine Liverpool UK; ^9^ Department of Infectious Diseases French National Public Health Agency Saint Maurice France; ^10^ Department of Neurology Medical University Innsbruck Innsbruck Austria; ^11^ Division of Infectious Diseases, Department of Medicine Karolinska Institute Stockholm Sweden; ^12^ Department of Neurosciences University of Florence Florence Italy; ^13^ Department of Neurology Careggi University Hospital Florence Italy; ^14^ Department of Neurology Erasmus University Medical Center Rotterdam the Netherlands; ^15^ Institute of Clinical Medicine University of Tartu Tartu Estonia; ^16^ Clinic of Neurology Tartu University Hospital Tartu Estonia; ^17^ Liverpool Interdisciplinary Neuroscience Centre University of Liverpool Liverpool UK; ^18^ Department of Neurology The Walton Centre for Neurology and Neurosurgery Liverpool UK; ^19^ Department of Infectious Diseases Imperial College Healthcare NHS Trust London UK; ^20^ Department of Infectious Diseases Imperial College London London UK; ^21^ The Francis Crick Institute London UK; ^22^ National Institute for Health and Care Research (NIHR) Health Protection Research Unit in Emerging and Zoonotic Infections Liverpool UK; ^23^ Department of Brain Sciences, Faculty of Medicine Imperial College London London UK; ^24^ Institute of Social and Preventive Medicine University of Bern Bern Switzerland; ^25^ Multidisciplinary Center for Infectious Diseases University of Bern Bern Switzerland; ^26^ Department of Neurovirology National Institute of Mental Health and Neurosciences Bangalore India; ^27^ Department of Neurology, Amsterdam UMC University of Amsterdam, Amsterdam Neuroscience Amsterdam the Netherlands; ^28^ Department of Infectious Diseases, Institute of Biomedicine, Sahlgrenska Academy University of Gothenburg Gothenburg Sweden; ^29^ Department of Infectious Diseases Sahlgrenska University Hospital Gothenburg Sweden; ^30^ Department of Neurology and Epileptology Hospital Nordwest Frankfurt Frankfurt Germany; ^31^ Encephalitis International Malton UK; ^32^ Department of Clinical Sciences Liverpool School of Tropical Medicine Liverpool UK; ^33^ The Pandemic Institute Liverpool UK

**Keywords:** encephalitis, GRADE, intervention, neurology, practice guidelines

## Abstract

**Background:**

Encephalitis is difficult to diagnose and manage, posing a challenge to clinicians. Since the last European guidelines were published in 2010, novel causes have emerged, diagnostic processes have evolved and treatments assessed which need to be reflected in updated guidelines.

**Objective:**

We aimed to produce recommendations for the diagnosis and management of infectious encephalitis in adults based on current evidence to equip clinicians to improve patient outcomes.

**Methods:**

These guidelines were developed jointly with the support of two European specialist medical organisations. Clinical questions were prioritised and formulated in PICO format. A systematic literature search was conducted for each question, and the quality of evidence was assessed using standardised tools. Recommendations were developed according to the Grading of Recommendations, Assessment, Development and Evaluations (GRADE) approach. Where only indirect evidence was available, a good practice statement was made.

**Results:**

Sixteen questions were considered, with two recommendations reached: for initiation of intravenous aciclovir as soon as possible upon suspicion of encephalitis and against replacing pathogen‐specific polymerase chain reaction (PCR) testing of cerebrospinal fluid (CSF) with multiplex CSF PCR panels for diagnosis of adult patients with suspected infectious encephalitis. For adults with proven HSV encephalitis, we were unable to recommend for or against the use of corticosteroids. A further 13 good practice statements regarding when to clinically suspect infectious encephalitis, microbiological investigations, additional investigations, treatment and ongoing care and rehabilitation were agreed.

**Conclusions:**

Given the clinical severity and public health importance of this condition, the paucity of high‐quality evidence is striking. Transnational, large‐scale research in infective encephalitis is encouraged.

## 1 Background

Encephalitis or inflammation of the brain parenchyma, is a life‐threatening condition associated with significant morbidity and mortality, with survivors frequently experiencing serious ongoing sequelae [1]. The incidence of encephalitis varies around the world, ranging from 0.5 to 16 per 100,000 population [2, 3, 4, 5]. Herpes simplex virus (HSV) is the most common cause of sporadic encephalitis among adults in Europe [6, 7, 8].

Encephalitis is difficult to diagnose and manage, posing a challenge to clinicians. Presenting symptoms, including fever and altered mental state, are non‐specific; only a minority of suspected cases actually have encephalitis [8]. Failure to consider encephalitis and instigate empirical treatment is common and associated with worse outcomes [9, 10]. Many aetiologies are not immediately distinguishable but require different treatments [11, 12]. The aetiology remains unexplained in a large fraction of cases; this varies from 20% to 70% in published studies [13]. Certain pathogens are more likely in individuals with altered immune status or chronic medical conditions (Table 1), and some presenting clinical features are traditionally associated with specific pathogens. Whether clinical features or risk factors can be used to reliably predict encephalitis aetiology remains unresolved.

**TABLE 1:** Infectious causes of acute and subacute/chronic encephalitis in immunocompetent and immunocompromised adults.

	Immunocompetent	Immunocompromised^a^
Acute	Herpesviruses (HSV‐1/2, VZV), enteroviruses, paramyxoviruses (measles, mumps), influenza viruses, adenovirus, parvovirus B19, LCMV, rubella virus, flaviviruses (WNV, JEV, TBEV, Dengue viruses), alphaviruses (WEEV, EEEV, VEEV), bunyaviruses (Lacrosse virus), coltiviruses (Colorado tick fever virus), rhabdoviruses (rabies virus, other lyssaviruses, Chandipura virus), Henipah viruses (Nipah)	Herpesviruses (EBV, CMV, HHV‐6 and ‐7, HSV‐1/2, VZV), *Cryptococcus neoformans* , *Listeria monocytogenes* , *Toxoplasma gondii*
Subacute/Chronic	Measles virus (SSPE), *Mycobacterium tuberculosis* , *Treponema pallidum* , *Borrelia burgdorferi*	Measles virus (inclusion body encephalitis), VZV (multifocal leukoencephalopathy), CMV, HSV (especially HSV‐2), HHV‐6, enteroviruses, JC/BK virus (PML), HIV (dementia), *C. neoformans* , *T. gondii*

*Note:* Reference [14].

Abbreviations: CMV, Cytomegalovirus; EBV, Epstein Barr virus; EEEV, Eastern equine encephalitis virus; HHV, Human herpesvirus; HIV, Human immunodeficiency virus; HSV, Herpes simplex virus; JEV, Japanese encephalitis virus; PML, Progressive multifocal leukoencephalopathy; SSPE, Subacute sclerosing panencephalitis; TBEV, Tick‐borne encephalitis virus; VEEV, Venezuelan equine encephalitis virus; VZV, Varicella zoster virus; WEEV, Western equine encephalitis virus; WNV, West Nile virus.

^a^ These causes affect immunocompromised individuals in addition to the causes listed for immunocompetent individuals.

Cerebrospinal fluid (CSF) obtained through lumbar puncture (LP) is the cornerstone of diagnostic testing in encephalitis. LP is a minimally invasive procedure but requires procedural expertise and may be contraindicated [15, 16, 17]. It is important to clarify when imaging is required before LP, as over‐reliance on computed tomography (CT) head imaging prior to LP may lead to unnecessary delays and negatively affect diagnostic yield and patient outcomes in central nervous system (CNS) infections [18, 19]. Even with timely LPs, some patients remain undiagnosed. Potential reasons for this include a low concentration of pathogens in CSF, insufficient sample volume to perform the required tests, compromised sample integrity through delayed transport or processing and limitations in the sensitivity of available tests [20]. A lack of CSF pleocytosis may result in wrongly excluding encephalitis as a diagnosis [21]. Repeat LP can be necessary for some diagnoses [22]. There is an increasing availability of multiplex polymerase chain reaction (PCR) panels for pathogen detection in the CSF, which may use less CSF volume, and may reduce turnaround time and staff workload [23, 24, 25]. However, multiplex assays may be less sensitive than single‐target assays and appropriate validation is essential [23]. Serological assays detecting intrathecal antibodies improve diagnostic yield but are insufficiently used in clinical practice [20]. Antibody tests are the mainstay for diagnosis of encephalitis caused by arboviruses, such as the flaviviruses West Nile virus and tick‐borne, but are susceptible to false‐positives and cross‐reaction between antibodies to different flaviviruses following previous infection or immunisation [26]. Serum and CSF testing frequently remain negative, and the cause of a suspected case can remain unclear [13]. Brain imaging using CT and magnetic resonance imaging (MRI) are often undertaken before CSF sampling and can reveal characteristic aetiological features (e.g., temporal lobe enhancement in HSV encephalitis) as well as exclude alternative diagnoses [27]. However, these are not usually relied on to distinguish causes of encephalitis. In patients remaining undiagnosed despite extensive diagnostics, a brain biopsy may be considered, however the procedure has potential risks [28].

Treatment for encephalitis depends on the underlying cause. Common causes of encephalitis, such as HSV and varicella zoster virus (VZV), are treated effectively with aciclovir [29]. Uncertainty remains regarding aciclovir duration and the necessity to demonstrate viral clearance from the CSF [11, 30]. A long duration of genome detectability has been found to correlate with poor outcome in HSV encephalitis [31]. There is also significant interest in the benefit of immunomodulation with steroid therapy in HSV encephalitis [32, 33]. Other infectious aetiologies have no proven treatment; therapies such as interferon, ribavirin, immunoglobulin and steroids have been trialled with uncertain efficacy [34, 35, 36, 37, 38].

Critically, there are no recent European guidelines to advise on navigating these uncertainties in diagnosis and management of patients with suspected infectious encephalitis. The latest European guidelines were published in 2010 (focusing on ‘viral meningoencephalitis’); United Kingdom (UK) guidelines on viral encephalitis were published in 2012 and French guidelines on infectious encephalitis were published in 2017 [14, 39, 40]. Since this time, novel causes of encephalitis have emerged and diagnostic processes have evolved, which need to be reflected in updated guidelines.

## 2 Objectives and Scope

We aimed to produce recommendations for the diagnosis and management of infectious encephalitis in adults based on current evidence and expert opinion to optimise diagnosis and treatment and improve patient outcome. The target audience of these guidelines includes all clinicians involved in the diagnosis, treatment and follow‐up of encephalitis in adults in the context of hospital care, including hospital physicians, infectious disease specialists, neurologists, intensive care specialists and microbiologists/virologists.

## 3 Methods

Development of this guideline followed the Grading of Recommendations, Assessment, Development and Evaluations (GRADE) framework and European Academy of Neurology (EAN) recommendations on the development of neurological management guidelines [41, 42]. A Task Force (TF) which consisted of 19 members was convened. TF members were experts in fields relevant to encephalitis (e.g., neurology, infectious disease, microbiology, neurocritical care, rehabilitation, patient representative) or guideline development and represented all geographical areas of Europe. For detailed methodology, including TF composition, see the full guidelines protocol in Appendix S1.

### 3.1 Questions

TF members identified relevant clinical and scientific questions, using previously published guidelines and their own practice and expertise [14]. Sixteen key questions were prioritised by the TF and formulated in Population/Intervention/Comparison/Outcome (PICO) format. Questions were grouped into five topics: when to clinically suspect infectious encephalitis; microbiological investigations; additional investigations; treatment; and ongoing care and rehabilitation. TF members were subdivided into Working Groups (WG), each assigned to one of the five topics.

### 3.2 Search for Studies

A predefined search strategy was devised for each question (see specific search strategies in Appendix S1) and used to search PubMed and the Cochrane Central Register of Controlled Trials (CENTRAL) for relevant studies from 1 January 2008 to 18 March 2021 (14 January 2022 for questions 1 and 2). January 2008 was chosen as the start date as the questions to be answered in these guidelines were informed by the previous UK guidelines which systematically searched the literature up until 2008 [14]. Relevant studies from the previous UK guidelines were included, and reference lists of included papers were reviewed for further relevant articles. A subsequent search was performed to identify new potentially relevant studies up to 26 January 2024.

To increase efficiency of the guidelines process, a number of approaches were taken, detailed in the study protocol in Appendix S1. One such approach was considering inclusion of systematic reviews alongside primary studies [43]. For searches yielding more than 1000 primary study results, the initial search was limited to systematic reviews. If a systematic review (assessed as moderate or high quality by Assessing the Methodological Quality of Systematic Reviews [AMSTAR]‐2 or similar) was found, the search start date was reset to the latest search date of the systematic review.

### 3.3 Study Selection and Data Extraction

Two independent reviewers screened the titles/abstracts and full texts of articles, where necessary, for eligibility. The inclusion criteria for studies were as follows: (1) relevant to the question being addressed and (2) study design as defined by individual PICO criteria (see protocol in Appendix S1; eligible case series must include ≥ 5 cases). Exclusion criteria included primary studies with < 5 cases; editorials, letters and correspondence which did not report primary data; studies including only paediatric populations; studies in any language other than English for which reliable translation was not available; and animal‐only studies.

Data from each included study were extracted into a structured data extraction form. The quality of included studies was assessed using standardised tools: the Cochrane Collaboration's tool for assessing risk of bias in randomised trials (RoB 2); QUADAS‐2 for diagnostic accuracy studies; Risk of Bias in Non‐randomised Studies of Interventions (ROBINS‐I); Joanna Briggs Institute and Newcastle‐Ottawa Scale tools for non‐interventional observational studies; and AMSTAR‐2 [44, 45, 46, 47, 48, 49]. All extracted data and quality assessments were verified by a second reviewer. Differences of opinion were resolved through discussion (with arbitration by a third reviewer if necessary).

### 3.4 Data Synthesis and Certainty in the Evidence

Following data extraction, the body of evidence for each question was summarised in tables. Where appropriate, a meta‐analysis was conducted using standard Cochrane methodology [50]. All factors that might decrease (e.g., study limitations/risk of bias, inconsistency of results, indirectness of evidence, imprecision, publication bias) or increase (e.g., large effect sizes, out‐of‐sample validation of results and dose–response) certainty in the evidence were considered, using the GRADE approach, with resulting labels of high, moderate, low or very low certainty [42].

### 3.5 Formulation of Recommendations

The GRADE approach was used to make recommendations based on the findings, using the Evidence to Decision framework [51]. Strength (strong or conditional) and direction (for or against) of recommendations were determined based on balance between desirable and undesirable effects, certainty in the evidence, values and preferences, required resources, cost effectiveness, equity, acceptability and feasibility. A conditional recommendation was made when the balance was in favour or against a course of action, but this had the potential to vary substantially between different clinical situations, or the balance was not strongly in favour or against the action, such that it may be viewed differently by individual clinicians, patients or policymakers in a given scenario. Where a GRADE‐based recommendation was not deemed appropriate by the WGs and overall TF, due to only indirect evidence being available, a good practice statement was made, in accordance with published guidance from the GRADE Working Group [52]. This lists criteria to be met to justify formulation of a good practice statement and use of the Evidence to Decision process, outlining a structured process. The GRADE‐ADOLOPMENT methodology was also considered, so that existing recommendations from previous guidelines were considered before embarking on a new recommendation process, to prevent duplication of effort where possible [53]. Consensus on recommendations and good practice statements was first achieved within WGs and then endorsed by all TF members at a meeting held at the European Society of Clinical Microbiology and Infectious Diseases (ESCMID) Global conference in Barcelona, Spain on April 28, 2024. As none of the disagreements between the recommendations and statements proposed by the WGs and opinions from the TF remained unresolved after this meeting, a modified Delphi approach was not required to achieve consensus.

## 4 Results

Table 2 provides a summary of recommendations and good practice statements for each question. A brief justification is provided, along with notes that may support implementation of the guidelines in practice. Appendix S1 provides a summary of the evidence, as well as detailed reports of the systematic reviews for each question, including flow diagrams for inclusion of studies, characteristics of included studies and quality/risk of bias assessments. For all questions, results from the studies are provided in evidence summaries and/or descriptive tables; where formal meta‐analysis was deemed appropriate, forest plots and GRADE summary of findings tables are provided.

**TABLE 2:** Summary of recommendations.

Question	Recommendations/Good Practice Statements
When to suspect encephalitis	
1. Presenting clinical features—In adults with suspected encephalitis, how accurate are presenting clinical features in predicting an infectious aetiology compared to PCR?	The presence or absence of specific clinical features should not be relied upon to guide management of patients with suspected infectious encephalitis (*good practice statement*).
2. Presence of risk factors—In adults with infectious encephalitis, does the presence of risk factors (i.e., immune status, travel history, infectious contacts, pets, water exposure, past medical history) compared to their absence result in a higher risk of specific infectious aetiologies (as confirmed by PCR)?	A carefully taken history can reveal clues as to the cause of infectious encephalitis. Table 4 provides some risk factors suggestive of some specific aetiological agents of infectious encephalitis (*good practice statement*).
Microbiological investigations	
3. LP before imaging—In adults with suspected encephalitis, does LP before imaging result in deterioration or worse clinical outcome compared to no LP before imaging? 3a. What are the contraindications for an immediate LP?	All patients with suspected encephalitis should have an LP as soon as possible after admission, unless contraindicated (*good practice statement*; Table 5). If there are clinical symptoms or signs of raised intracranial pressure with potential brain shift, a CT or MRI scan should be performed as soon as possible to evaluate for contraindications and potential features of the disease process (*good practice statement*; Table 5). There should be no delay in performing an LP following imaging unless contraindications are present. If imaging is not needed before LP, imaging (CT or MRI) should be performed after the LP (*good practice statement*; Table 5). There are absolute and relative contraindications for performing LP in patients receiving anticoagulant or antiplatelet therapy. Reversal, if needed, can be performed according to local guidelines (*good practice statement*; Table 5). In situations where an LP is not possible at first, the situation should be reviewed at least every 12–24 h, and an LP performed when it is safe to do so (*good practice statement*).
4. Second LP—In adults with suspected encephalitis and a first non‐diagnostic LP, does a second LP 48 h later result in improved aetiological diagnosis?	If an initial LP is non‐diagnostic and the LP was performed within the first 3 days after onset of the neurological features, a second LP should be performed 2–3 days later if there is an ongoing suspicion of encephalitis (*good practice statement*).
5/5b. In suspected sporadic infectious encephalitis is individualised/specific CSF PCR better than standardised/multiplex CSF PCR panel for first‐line detection of the causative pathogen? Are there additional specific testing panels for immunocompromised patients? 5a. Antibodies—Which CSF antibody testing is useful in the diagnosis of infectious encephalitis?	We conditionally recommend against replacing pathogen‐specific PCR testing of CSF with multiplex CSF PCR panels for diagnosis of adults with suspected infectious encephalitis (*conditional recommendation, low certainty in the evidence*). See Table 6 for guidance specific on CSF antibody testing by pathogen (*good practice statement*). Guidance from an infection specialist should be sought in deciding on and interpreting results from these investigations.
6. Brain biopsy—In patients with suspected infectious encephalitis and no aetiological diagnosis despite extensive investigation, who are deteriorating, does brain biopsy change patient management compared to no brain biopsy?	Brain biopsy has no place in the initial assessment of suspected acute infectious encephalitis (*good practice statement*). Biopsy may inform diagnosis and can influence management when there is no aetiological diagnosis despite extensive investigation, but the potential benefits should be balanced against the potential risks for the individual patient (*good practice statement*). Where possible, focal abnormalities on imaging should guide sampling using targeted stereotactic techniques (*good practice statement*). If imaging shows no focal abnormality, a blind biopsy may be considered (*good practice statement*).
Additional investigations	
7. CT/MRI—In adults with suspected encephalitis, what is the diagnostic accuracy of specific features on CT and MRI to predict an infectious aetiology compared to PCR?	It can be helpful to identify specific patterns of involvement of the brain on MRI to support suspicion of a particular pathogen pending PCR results (*good practice statement*). MRI (including contrast‐enhanced diffusion‐weighted imaging) is the preferred modality and should be performed as soon as possible in suspected infectious encephalitis; ideally within 24 h of hospital admission, certainly within 48 h (*good practice statement*). If the patient's condition precludes MRI scan, or MRI is not available, urgent CT scanning may help evaluate alternative causes of the patient's presentation or potential contra‐indications to immediate LP. However, diagnostic accuracy of CT is not comparable to MRI for making a diagnosis of infectious encephalitis, and CT is less accurate than clinical features for predicting potential complications from an LP (*good practice statement*).
8. EEG—In patients with suspected infectious encephalitis and psychiatric features, does EEG change the patient management compared to no EEG?	EEG need not be performed routinely in all patients with suspected infectious encephalitis (*good practice statement*). For patients with uncertainty whether there is a psychiatric or organic cause, an EEG should be performed to seek encephalopathic changes, as this may influence management (*good practice statement*). EEG should also be performed if subtle motor, or nonconvulsive seizures are suspected (*good practice statement*).
Treatment	
9. Treatment with aciclovir—In patients with suspected encephalitis, does treatment with aciclovir improve patient outcome compared to no aciclovir administration?	IV aciclovir should be started as soon as possible after suspicion of encephalitis (ideally within 6 h from presentation to hospital). In some situations, other antimicrobial treatments should be considered alongside aciclovir (s*trong recommendation, low certainty in the evidence*).
9a. If HSV is not confirmed when can aciclovir be stopped?	If there is a low clinical suspicion of HSV encephalitis, or a confirmed alternative diagnosis, aciclovir can be stopped if ALL of the following apply: (1) negative CSF PCR for HSV (if LP was > 72 h after onset of neurological illness), (2) normal CSF white blood cell count (ignore if an alternative diagnosis would explain a raised count) and (3) no temporal lobe abnormality on MRI of the brain. If there is a high clinical suspicion of HSV encephalitis, aciclovir may be continued despite negative HSV PCR on CSF, absence of temporal lobe encephalitis on MRI brain, and despite an alternative diagnosis being made. In such cases, re‐evaluation should be performed in consultation with an infection specialist and/or neurologist, including repeat CSF examination and brain imaging (*good practice statement*).
10. Duration of aciclovir treatment—In patients with proven HSV encephalitis (positive CSF PCR or intrathecal antibody production), does administering aciclovir for more than two weeks reduce morbidity compared to two‐week aciclovir therapy? 10a. When should aciclovir resistance be suspected and what action should be taken?	In patients with proven HSV encephalitis, a minimum of 14 days of therapy with IV aciclovir should be administered (*good practice statement*). In patients who are improving, it is not necessary to repeat CSF examination before stopping therapy at 14 days (*good practice statement*). In patients who have not improved, are clinically worse, or who are immunocompromised, extension of therapy to 21 days or even 28 days, can be considered. In this case, repeat CSF examination for leukocyte count and HSV PCR may be of benefit, with continuation of aciclovir until results are available (*good practice statement*). Aciclovir resistance in encephalitis is very rare and should not be assumed to be the cause of progressive disease (*good practice statement*). In a patient with progressive disease despite 14 days of IV aciclovir therapy, assessment of the likelihood of aciclovir resistance must be performed alongside exclusion of alternative causes of deterioration. Switching therapy to foscarnet and testing for aciclovir resistance may be considered, in consultation with a neurologist or infection specialist (*good practice statement*). In patients who are immunosuppressed, and/or taking long‐term aciclovir prophylaxis, there may be a lower threshold for considering aciclovir resistance, though it is still considered to be rare in the context of encephalitis (*good practice statement*).
11. Corticosteroids—In patients with proven HSV encephalitis, does the addition of corticosteroid therapy improve outcome compared to standard care alone? 11a. If yes, when should corticosteroids be introduced?	For adults with proven HSV encephalitis, we are unable to recommend for or against the use of corticosteroids.
12. Aciclovir dose in VZV—In patients with proven VZV encephalitis (positive CSF PCR or intrathecal antibody production), does treatment with high dose aciclovir (usually 20 mg/kg three times per day) compared to 10 mg/kg aciclovir three times per day reduce mortality and morbidity?	Patients with proven VZV encephalitis should be treated with a standard dose of 10 mg/kg aciclovir three times daily (if renal function is normal). If an associated vasculitis is confirmed, corticosteroid therapy should be added; in the absence of evidence of vasculitis, empirical corticosteroids should not be started for this indication (*good practice statement*).
13. Prophylactic antiseizure therapy/medication—What is the impact on morbidity and mortality of prophylactic antiseizure treatment versus no prophylactic antiseizure treatment in patients with infectious encephalitis?	From the available indirect evidence, the balance between benefit and potential harm is *against* starting primary prophylaxis (without seizures as part of the illness). Management of seizures in the acute phase should be as per existing guidelines. A decision to continue the antiseizure medication after the acute hospital admission should be individualised based on various factors, including extent and location of abnormalities on brain imaging and number and timing of seizures, in discussion with the patient and the multidisciplinary team (*good practice statement*).
Ongoing care and rehabilitation	
14. Admission to intensive care—In patients with infectious encephalitis and deep/worsening coma score or status epilepticus, does admission to intensive care improve outcome and reduce mortality compared to no admission to intensive care?	Patients with infectious encephalitis and falling level of consciousness or status epilepticus (including non‐convulsive status epilepticus) require admission to an ICU, or a neuro‐critical care unit, if available, for assessment for airway protection and ventilatory support; management as needed of blood pressure, intracranial pressure and cerebral perfusion pressure (using invasive monitoring where appropriate); and correction of metabolic imbalances, including electrolytes, carbon dioxide and glucose (*good practice statement*).
15. HSV encephalitis relapse—In a patient with HSV encephalitis with recurrent/worsening symptoms, does autoantibody testing improve the chance of identifying the cause of relapse compared to not testing for autoantibodies?	In a patient with recent HSV encephalitis with recurrent or worsening symptoms, CSF should be tested for anti‐NMDA receptor antibodies. Testing for other autoantibodies should also be considered (*good practice statement*).
16. Rehabilitation—In patients with infectious encephalitis, does follow‐up care and rehabilitation reduce morbidity compared to no follow‐up care and rehabilitation?	All patients with encephalitis should receive periodic assessment and rehabilitation at each stage of their encephalitis journey. This should be tailored to each patient, in terms of stage and severity of illness, its impact and to their goals, values and preferences (*good practice statement*). Patients (when conscious level permits) and their caregivers should be made aware of the support provided by the health and social care system of their country of residence, and also by non‐governmental organisations such as Encephalitis International (www.encephalitis.info) (*good practice statement*). Patients (and caregivers) should not be discharged from hospital without communication about their diagnosis and its potential impact on their life. Arrangements for outpatient follow‐up and plans for on‐going therapy and rehabilitation should be formulated prior to hospital discharge (*good practice statement*). All patients should be given access to assessment for rehabilitation (*good practice statement*).

Abbreviations: CSF, Cerebrospinal fluid; CT, Computed tomography; EEG, Electroencephalogram; HSV, Herpes simplex virus; ICU, Intensive care unit; IV, Intravenous; LP, Lumbar puncture; MRI, Magnetic resonance imaging; NMDA, N‐methyl D‐aspartate; PCR, Polymerase chain reaction; VZV, Varicella zoster virus.

### 4.1 When to Suspect Encephalitis

***Background question: What are the typical presenting clinical features of infectious encephalitis (including possible variations due to altered immune status)?***

The classical clinical features of infectious encephalitis include fever and abnormal mental status, often with severe headache, nausea and vomiting [14]. Altered mental state, seizures, fever and aphasia have been identified as common features in HSV encephalitis, whereas focal neurological symptoms and rash were most commonly seen in VZV encephalitis in a UK prospective study [8, 62, 63, 64]. A large Indian prospective cohort study reported seizures in three quarters of patients with HSV encephalitis [64]. Immunocompromised patients with infectious encephalitis are more likely to present with focal neurological deficits and confusion and to have low CSF glucose concentration and high CSF neutrophil count than immunocompetent patients [65].

***1. In adults with suspected encephalitis, how accurate are presenting clinical features in predicting an infectious aetiology compared to PCR?***

***Good Practice Statement***

The presence or absence of specific clinical features (e.g., altered behaviour, seizures, coma, fever, focal neurological signs) should not be relied upon to guide management of patients with suspected infectious encephalitis.

***Justification***

Six relevant studies were found from which data for two studies could be pooled through meta‐analysis [8, 62, 63, 64, 66, 67]. As none of the studies were designed to primarily compare clinical features against a gold standard of PCR for a specific pathogen, the evidence was considered too indirect for a recommendation. The results, though of very low certainty, suggest suboptimal performance of the presence of altered behaviour, seizures, coma, fever and focal neurological signs in predicting HSV and VZV as causative pathogens (Tables S1.2 and S1.3).

***Practical Notes***

Even though clinical features cannot definitively distinguish between aetiologies, some specific features may point towards a specific cause and prompt further investigation (Table 3).

**TABLE 3:** Clinical features suggestive of encephalitis aetiologies in adults^a^.

Clinical feature	Pathogen/disease to consider^b^
*Neurological*	
Abnormal behaviour (new psychosis, mania, delirium, aggression, agitation, catatonia)	Autoimmune conditions^c^
Myoclonus	Measles virus/SSPE, *Tropheryma whipplei* , Prion disease, Autoimmune conditions^c^
Brainstem features, including autonomic dysfunction, bulbar signs and cranial neuropathies^d^	*Listeria monocytogenes* , *Brucella* species, *Mycobacterium tuberculosis* and some viral CNS infections
Bulbar or ocular dysfunction	HHV‐6, *L. monocytogenes* , *T. whipplei* , Autoimmune conditions^c^
Rapid neurological decompensation^4^	Rabies virus
Parkinsonism or tremor	Flaviviruses (WNV, JEV, SLEV, TBEV, DENV), *Toxoplasma gondii*, *T. whipplei* , *Borrelia burgdorferi*
Acute flaccid paralysis	Polio virus, other enteroviruses, flaviviruses (e.g., tick‐borne encephalitis virus, WNV), rabies virus
Cerebellar ataxia	VZV, mumps virus, *T. whipplei*
*Other*	
Skin rash, prior or current	VZV (vesicular/polymorphic), HHV‐6, WNV, rubella virus, enteroviruses (palms and soles and/or oral ulcers), HIV, *Rickettsia* (palms and soles, papular), *Mycoplasma pneumoniae* , *Borrelia* species, *Treponema pallidum* (palms and soles, preceding genital ulcer), measles virus (papular), dengue virus (macular or petechial), *Neisseria meningitidis* (petechial)
Chronic skin ulcer	*Acanthamoeba, Balamuthia*
Eschar	*Orientia tsutsugamushi* , *Rickettsia* species
Lymphadenopathy	*Bartonella henselae* , HIV, EBV, CMV, measles virus, rubella virus, *T. pallidum* , *M. tuberculosis* , *Orientia tsutsugamushi*
Hepatitis	*Coxiella burnetii* , *O. tsutsugamushi* , *DENV*
Parotitis and/or epididymo‐orchitis	Mumps virus, CMV
Pulmonary abnormalities	Influenza virus, *M. pneumoniae, C. burnetii, M. tuberculosis, Histoplasma capsulatum, Cryptococcus neoformans, SARS‐CoV‐2, Nocardia species*
Retinitis	CMV, HSV, VZV, *B. henselae, T. pallidum, HIV, Toxoplasma gondii*
Hydrophobia	Rabies virus

*Note:* References [14, 54].

Abbreviations: CMV, Cytomegalovirus; CNS, Central nervous system; DENV, Dengue virus; EBV, Epstein Barr virus; EV‐71, Enterovirus‐71; HHV‐6, Human herpesvirus 6; HIV, Human immunodeficiency virus; HSV, Herpes simplex virus; JEV, Japanese encephalitis virus; SLE, St. Louis encephalitis; SSPE, Subacute sclerosing panencephalitis; TBEV, Tick‐borne encephalitis virus; VZV, Varicella zoster virus; WNV, West Nile virus.

^a^ In Europe, HSV is the most likely pathogen with any presenting features above and therefore is only mentioned alongside presenting features that are particularly specific to HSV encephalitis.

^b^ This is not an exhaustive list.

^c^ Autoimmune encephalitis, like anti‐NMDAR encephalitis and others.

^d^ Some pathogens are prone to causing raised intracranial pressure, which may present with ‘false‐localising’ cranial nerve signs, most commonly abducens nerve palsy or rapid neurological decompensation. These include *Cryptococcus neoformans* , *Mycobacterium tuberculosis* and *Histoplasma capsulatum*. Pyogenic meningitis (usually due to *Streptococcus pneumoniae* , *Neisseria meningitidis* or *Haemophilus influenzae* ) can also present with such features, which is a major differential diagnosis of infectious encephalitis.

***Subgroups***

Clinical features for individual pathogens may differ between immunocompetent and immunocompromised individuals, though the evidence for specific pathogens is lacking.

***Research Needs***

Studies should be conducted to compare presenting clinical features against a gold standard for diagnosis of common encephalitis pathogens to support clinicians with decisions about diagnostics and empirical therapy.

***2. In adults with infectious encephalitis, does the presence of risk factors (i.e., immune status, travel history, infectious contacts, pets, water exposure, past medical history) compared to their absence result in a higher risk of specific infectious aetiologies (as confirmed by PCR)?***

***Good Practice Statement***

While evidence from well conducted large studies is lacking, a carefully taken history can reveal clues as to the cause of infectious encephalitis. Table 4 provides some risk factors (e.g., immunocompromise, recent infection, travel, animal exposure) suggestive of some specific aetiological agents of infectious encephalitis.

**TABLE 4:** Risk factors suggestive of encephalitis aetiologies in adults^a^.

Risk factor/exposure	Pathogen/disease to consider
Mosquito bites	WNV, SLEV, WEEV, JEV, La Crosse, MVEV, dengue virus, Usutu virus, Chikungunya virus, Zika virus
Tick bite	TBEV, Powassan virus, *Borrelia* species
Animal exposure	
Dog	Rabies virus
Bat	Rabies virus, Nipah virus
Bird	Usutu virus, WNV
Pig	Nipah virus, JEV
Cat	*Bartonella* species, *Toxoplasma gondii*
Rodent	LCMV, *Leptospira* species, Lassa virus, Hanta virus, *Francisella tularensis*
Macaque primate	Herpes B virus
Sheep/goat	*Coxiella burnetii*
Raccoon	*Baylisascaris procyonis*
Animal urine	*Leptospira* species
Travel	
Asia	JEV (China, Southeast Asia, Indian subcontinent), Nipah virus (Malaysia, Singapore, Bangladesh, India), TBEV (Russia, China, Japan), dengue virus (widespread), Chikungunya virus (widespread), *Orientia tsutsugamushi* (scrub typhus; widespread), Rabies virus
Europe	TBEV (central, eastern and northern countries), Toscana virus, WNV
Africa	Chikungunya, *Plasmodium falciparum* (malaria), dengue virus, Lassa virus (West Africa), Yellow fever virus
North America	WNV, EEEV, La Crosse virus, SLEV, WEEV
South America	Zika virus, Chikungunya virus, dengue virus, yellow fever virus, EEEV
Illness exposure	
Chickenpox	VZV
Measles	Measles virus
Respiratory infection	Mycoplasma, influenza virus, SARS‐CoV‐2, enterovirus, *Streptococcus pneumoniae*
Meningitis or sepsis	*Neisseria meningitidis*
Food exposure	
Unpasteurised milk and milk products	TBEV, *Listeria monocytogenes, Brucella* species, *C.burnetii*
Fresh water	*Naegleria fowleri, Leptospira* species, *Acanthamoebae* species
Soil	*Balamuthia mandrillaris, Acanthamoeba* species
Immunocompromise	HIV, *T.gondii, Cryptococcus neoformans, Mycobacterium tuberculosis*, CMV, JC virus, HHV‐6, *L.monocytogenes*
Occupation	
Forest workers	TBEV, *Borrelia* species
Work with animals	Rabies virus, Leptospira

*Note:* References [54, 55, 56, 57].

Abbreviations: CMV, Cytomegalovirus; EEEV, Eastern equine encephalitis virus; HHV‐6, Human herpesvirus 6; HIV, Human immunodeficiency virus; JEV, Japanese encephalitis virus; LCMV, Lymphocytic choriomeningitis virus; MVEV, Murray Valley encephalitis virus; SLEV, St. Louis encephalitis virus; TBEV, Tick‐borne encephalitis virus; VZV, Varicella zoster virus; WEEV, Western equine encephalitis virus; WNV, West Nile virus.

^a^ It is important to note that this list is not complete and prioritises pathogens of greatest importance in Europe.

***Justification***

Four potentially relevant studies were found from which data could not be pooled through meta‐analysis, nor could a recommendation be made due to missing data, indirectness and not primarily performing a risk factor analysis for causative pathogens [8, 63, 66, 68]. Two that aligned closely with the PICO suggested older age may predict HSV as a causative pathogen, but the certainty in this was too low and overall, the evidence was too indirect to support a recommendation (Table S2.2) [8, 68].

***Practical Notes***

Even though risk factors cannot definitively distinguish between aetiologies, some specific risk factors may point towards a specific cause and prompt further investigation (Table 4). Clinicians should remain informed about the spread of previously tropical or geographically restricted pathogens (e.g., TBEV, Chikungunya, dengue) as the epidemiology of infective encephalitis evolves due to population movements and climate change.

***Subgroups***

These are covered in Table 4.

***Research Needs***

Large multicentre prospective studies are required to inform risk factors for specific infectious aetiologies.

### 4.2 Microbiological Investigations

***3. In adults with suspected encephalitis, does LP before brain imaging result in deterioration or worse clinical outcome compared to no LP before brain imaging?***

***3a. What are the contraindications for LP?***

***Good Practice Statements***

- All patients with suspected encephalitis should have an LP as soon as possible after hospital admission, unless there is a contraindication.
- If there are clinical symptoms or signs of raised intracranial pressure with potential brain shift (Table 5), a brain CT or MRI scan should be performed as soon as possible to evaluate for contraindications and potential features of the disease process. Aciclovir therapy should not be delayed awaiting imaging or LP (Figure 1).
- There should be no delay in performing an LP following imaging unless contraindications are present (Table 5). If imaging is not needed before LP, imaging (CT or MRI) should be performed after the LP.
- There are absolute and relative contraindications for performing LP in patients receiving anticoagulant or antiplatelet therapy. Reversal, if needed, can be performed according to local guidelines.
- In situations where an LP is not possible at first, the situation should be reviewed at least every 12–24 h, and an LP performed when it is safe to do so.

**TABLE 5:** Contraindications for LP.

When to suspect raised intracranial pressure with potential brain shift, which indicates brain imaging should be performed before LP
Altered mental status (GCS < 10)
New‐onset seizures
Focal neurological signs, such as hemiparesis, aphasia, hemianopsia/hemianopia and cranial nerve dysfunction^a^
Presence of papilloedema^b^
Severe immunocompromise (e.g., untreated HIV, transplant recipient, immunosuppressive therapy)
Situations which may prevent immediate LP, independent of imaging findings
Local infection at LP site
Severely abnormal coagulation tests, if performed^c^
Low platelet count (< 40 × 10^9^/L)^c^, ^d^
Anticoagulant or antiplatelet therapy (except for low‐dose [75 mg once daily] aspirin/acetylsalicylic acid, which can be continued)^e^

Abbreviations: GCS, Glasgow coma scale; HIV, Human immunodeficiency virus; INR, International normalised ratio; LP, Lumbar puncture.

^a^ Although relative bradycardia with hypertension and abnormal ‘doll's eye’ movement are included as contraindications in other guidelines, they were felt to be unhelpful and rarely occur in the absence of other focal neurological signs. While cranial nerve signs are excluded from focal neurological abnormalities when considering contraindications to lumbar puncture in the European meningitis guidelines [58], in the context of suspected encephalitis, cranial nerve signs are more likely to indicate a focal lesion which may predispose to the rare event of brain herniation during lumbar puncture.

^b^ If full visualisation of the optic discs is not possible, or there is uncertainty, the inability to rule out papilloedema should not be the sole criterion for brain imaging before LP.

^c^ These thresholds vary between local and/or national guidelines and protocols, which should be consulted where available.

^d^ If there is evidence of platelet dysfunction, local expert input should be sought.

^e^ This should be guided by local and/or national guidelines and protocols, and a clinical assessment of benefit‐versus‐risk, for both performing‐versus‐deferring the lumbar puncture and for stopping‐versus‐continuing the therapy. Key factors include the indication for and dose of the anticoagulant or antiplatelet agents, and the potential harm from delaying the lumbar puncture and therefore extending diagnostic uncertainty. An example of clinical guidelines on this, incorporating these factors, has been made by the Association of British Neurologists [59].

**FIGURE 1:**
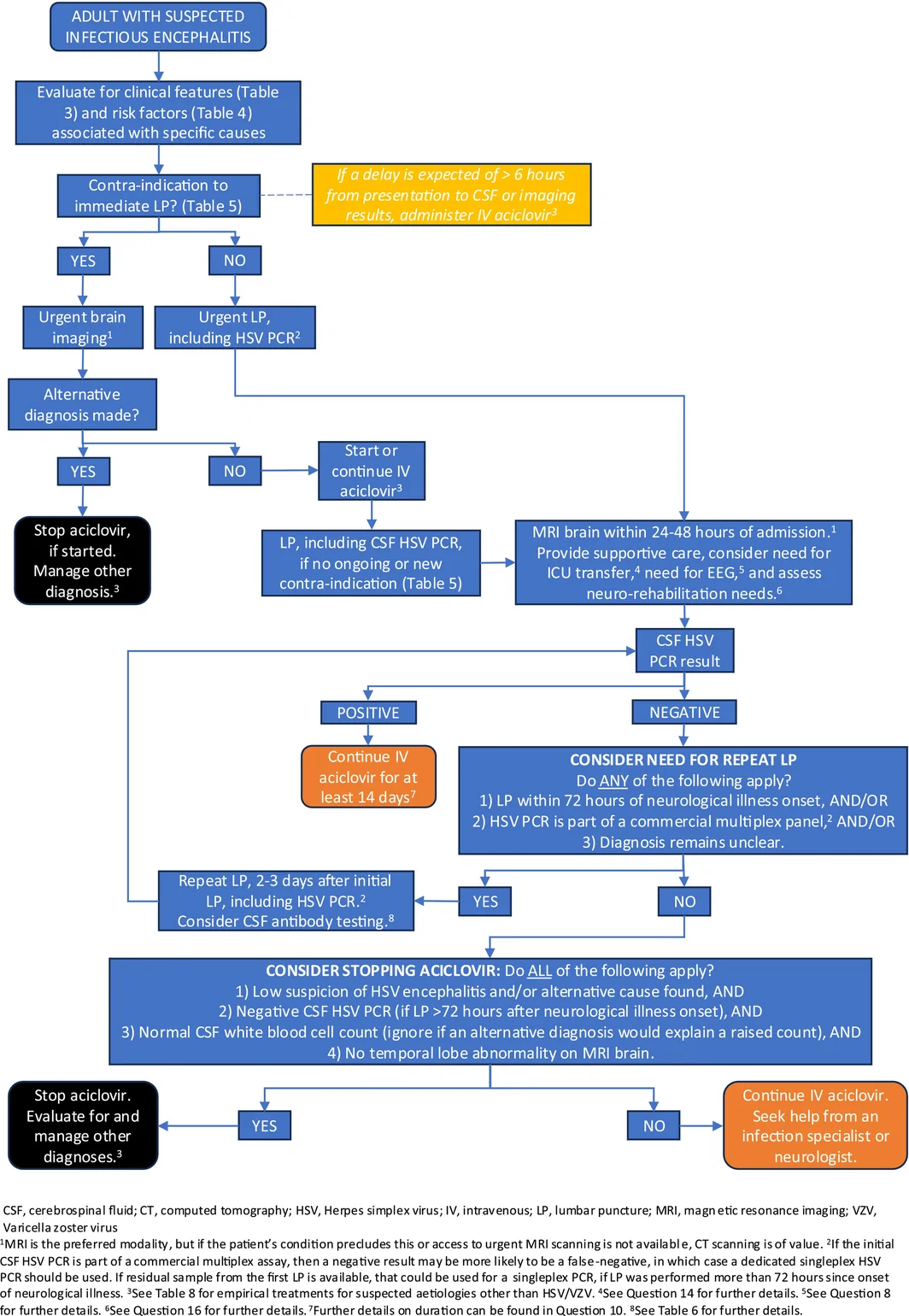
Algorithm for management of adults with suspected infectious encephalitis. Adapted from [14, 69].

***Justification***

No eligible studies were found which compare outcomes with LP before imaging to no LP before imaging; this meant a recommendation could not be justified. It has been shown in acute bacterial meningitis that LP without prior CT scan is associated with significantly earlier treatment and a favourable outcome [19]. Four studies were identified that reported on contraindications for LP [70, 71, 72, 73]. Contraindications such as focal neurological signs and papilloedema were reported in most studies, while coagulation abnormalities, for example, were only mentioned in a few. Suggestions regarding contraindications from the ESCMID guideline on bacterial meningitis were also incorporated [58].

***Practical Notes***

Potential contraindications not included in Table 5 may be included in local and national guidelines which should also be considered when making this decision. Ultimately, to arrive at the best possible decision a good health care organisation is required which provides access to expertise and clinical experience at patient admission. While the inability to obtain CSF should not delay IV aciclovir therapy, brain imaging may provide alternative diagnoses. Therefore, within the period of 6 h after presentation to hospital (see question 9), it may be beneficial to wait for imaging before administering aciclovir.

***Subgroups***

The same contraindications should apply for immunocompromised as for immunocompetent individuals, though it is recognised that severe immunocompromise is itself a contraindication according to some authorities.

***Research Needs***

Recent direct comparisons of outcomes following LP with and without prior imaging are required to inform clinical decision‐making about the use of imaging for patients presenting with suspected encephalitis.

***4. In adults with suspected encephalitis and a first non‐diagnostic LP, does a second*** ***LP*** ***48 h later result in improved aetiological diagnosis?***

***Good Practice Statement***

If an initial LP does not confirm a diagnosis, and the LP was performed within the first 3 days after onset of the neurological features, a second LP should be performed 2–3 days later if there is an ongoing suspicion of encephalitis.

***Justification***

No article met the inclusion criteria, however, the WG decided that a good practice statement based on indirect evidence was necessary to ensure optimal care. In HSV encephalitis, it has been shown that a first PCR, usually within 3 days of onset of the neurological illness, may be negative whereas a repeat PCR is positive [74, 75, 76]. In addition, evolution of CSF parameters such as differential leukocyte count may be useful in the diagnosis of other pathogens, e.g., enteroviruses and *Mycobacterium tuberculosis* may cause an initial predominance of polymorphonuclear cells (polymorphs) which switches to mononuclear (lymphocyte) predominance later in the illness [77].

***Practical Notes***

Pending a second HSV PCR result, if the patient is considered to have a high likelihood of infectious encephalitis, aciclovir should be continued, unless an alternative diagnosis is established. The aim should be for LP to be repeated as close as possible to 48 h after the initial LP, to avoid delay in making a diagnosis and informing management. In addition to repeating leukocyte count (total and differential), protein, glucose and PCR for HSV and other viruses, further tests should be performed according to the clinical situation (e.g., larger volume sample for *M.tuberculosis* or tests for other pathogens), in consultation with an infectious disease specialist and/or neurologist.

***Subgroups***

There was a lack of evidence for guidance focusing on specific subgroups.

***Research Needs***

Studies are required to address this question specifically.

***5/5b. In suspected sporadic infectious encephalitis is individualised/specific CSF PCR better than standardised/multiplex CSF PCR panel for first‐line detection of the causative pathogen? Are there additional specific testing panels for immunocompromised patients?***

***Recommendation***

We conditionally recommend against replacing pathogen‐specific PCR testing of CSF with multiplex CSF PCR panels for diagnosis of adult patients with suspected infectious encephalitis (*Conditional recommendation; low certainty in the evidence*).

***Justification***

The main concern about current CSF multiplex PCR panels is the high risk of false‐negative results for HSV‐1, the most common cause of sporadic infectious encephalitis in adults in Europe. The corresponding Section 5 (Tables S5.2–S5.5) in Appendix S1 provides the full Evidence to Decision which underpins the recommendation. Two cross‐sectional studies met the inclusion criteria, and a further systematic review/meta‐analysis was subsequently identified and included [23, 78, 79]. The most widely studied multiplex PCR panel used in suspected encephalitis is the BioFire FilmArray Meningitis/Encephalitis (ME) Panel. This includes targets for 14 pathogens with the potential to cause meningitis or encephalitis including HSV‐1. However, in a meta‐analysis of three studies (6883 participants) comparing the FilmArray ME against pathogen‐specific ‘single‐plex’ PCR, pooled sensitivity for HSV‐1 PCR was 76% (see Table S5.7 for quality assessment using the Preferred Reporting Items for Systematic reviews and Meta‐Analyses (PRISMA) checklist for diagnostic test accuracy). Though the certainty in this estimate was very low, this raised enough concerns about the ability of the FilmArray ME and other multiplex PCR panels to ‘rule out’ the most important treatable viral pathogen. While pooled sensitivity for other key encephalitis pathogens was better (VZV: 91%; HSV‐2: 94%; enterovirus: 94%) and pooled specificity was 99%–100% for all, the certainty in the evidence for these estimates was also low or very low. In addition, false‐positive results have been reported widely, especially for less common pathogens. Despite the potential for earlier results and reduced need for human, consumable and infrastructure resources in the laboratory, this prompted the WG to decide on a recommendation against use of multiplex PCR in place of pathogen‐specific PCR for testing CSF in patients with suspected infectious encephalitis.

***Practical Notes***

The WG considered that some laboratories have the BioFire FilmArray or other similar platforms in place for encephalitis as well as other syndromes. In such situations, if a negative PCR for HSV‐1 on the multiplex panel can be followed by a dedicated single‐plex HSV‐1 PCR, that may be an adequate approach. However, it would mean that decision making for patient care may need to be deferred until that second PCR result. Communicating the initial negative result may be challenging to ensure this does not lead to premature cessation of antiviral therapy or other potential harmful effects.

***Subgroups***

The available evidence did not suggest a benefit for multiplex CSF PCR panels in specific subgroups of patients with suspected infectious encephalitis, for example, immunocompromised individuals.

***Monitoring and Evaluation***

All laboratories and their referring clinical units using multiplex PCRs for CSF testing in infectious encephalitis should perform a local evaluation to compare these against dedicated single‐plex PCRs, as accuracy and impact of a positive or negative test may vary in different settings and for different patients.

***Research Needs***

To inform the use of multiplex PCRs for encephalitis in adults, future studies should include more patients with HSV encephalitis, as well as disaggregated results for adults with suspected encephalitis (rather than combined with meningitis). The uncertainty in sensitivity estimates in recent systematic reviews highlights the urgent need for head‐to‐head clinical diagnostic test accuracy studies of single‐plex versus multiplex HSV PCRs [23, 80]. As a significant proportion of encephalitis cases remain undiagnosed, novel diagnostics are needed, particularly the development of untargeted approaches. Cost‐effectiveness studies in microbiological diagnostics of infectious encephalitis would be beneficial, as would studies on metagenomics and proteomics to possibly detect more aetiologies in encephalitis of hitherto unknown origin.

***5a. Which CSF antibody testing is useful in the diagnosis of infectious encephalitis?***

***Good Practice Statement***

See Table 6 for Good Practice Statement by pathogen. Guidance from a specialist in infectious diseases or clinical microbiology/virology should be sought in deciding on and interpreting results from these investigations.

**TABLE 6:** Good practice statements for CSF antibody testing by pathogen.

Pathogen	Good Practice Statements
Herpesviruses (HSV, VZV and others)	For patients with suspected encephalitis in whom HSV PCR of the CSF was not performed early in the illness, a paired CSF and serum sample taken 7–10 days from onset of the neurological illness should be sent for HSV‐specific IgG antibody testing. For patients with suspected encephalitis in whom HSV PCR of the CSF was found to be repeatedly negative during the first week, and no other cause has been found, a CSF and a serum sample should be sent for HSV‐specific IgG antibody testing. Testing for intrathecal antibodies^a^ against other herpesviruses may be useful, but this should be decided on a case‐by‐case basis.
Flaviviruses (e.g., TBEV, JEV, WNV)	In suspected flavivirus encephalitis, CSF IgM antibody testing is diagnostic and should be repeated if initially negative, if the suspicion remains. If the individual is previously vaccinated IgM response may be delayed or ameliorated, and intrathecal CSF IgG antibodies^a^ may be diagnostic.
SSPE	In patients with suspected SSPE, demonstration of intrathecal anti‐measles antibodies^a^ is diagnostic.
Borrelia	In patients with suspected neuroborreliosis, demonstration of intrathecal antibodies^a^ is diagnostic.
Imported pathogens	For returning travellers where there may be suspicion of encephalitis acquired from the place of travel, CSF antibody testing can assist in making a diagnosis, but a specialist in imported infections should be consulted.

Abbreviations: CSF, Cerebrospinal fluid; HSV, Herpes simplex virus; JEV, Japanese encephalitis virus; SSPE, Subacute sclerosing panencephalitis; TBEV, Tick‐borne encephalitis virus; VZV, Varicella zoster virus; WNV, West Nile virus.

^a^ This is the demonstration of antibody production within the central nervous system, rather than the antibodies having crossed into the CSF space from the blood [60, 61].

***Justification***

Four suitable studies were found, though they were small and focused on specific pathogens, providing evidence that was too heterogeneous and indirect for a recommendation to be made [20, 81, 82, 83]. CSF and serum testing for HSV‐specific IgG antibodies can increase overall diagnosis of HSV encephalitis, including when PCR for HSV is negative [20] and diagnosis can be made long after the onset of HSV encephalitis since intrathecal antibodies can persist for many years [84]. Evidence for the value of anti‐VZV IgG testing in addition to VZV CSF PCR is relatively lacking. A few studies have reported concurrent positivity by CSF PCR and VZV IgG antibodies in patients with VZV CNS infections [81, 85]; however, another study failed to detect intrathecal production in CSF PCR positive patients [20]. Anti‐TBEV IgM was detected in all and IgG antibodies in most CSF samples from patients infected in different areas of Europe who presented with neurological signs of TBE (*n* = 5) [82]. Diagnosis of subacute sclerosing panencephalitis is confirmed by demonstrating intrathecal production of measles‐specific IgG [20, 86].

***Practical Notes***

Timing of testing, and if relevant, re‐testing for antibodies in CSF should be decided with the help of a specialist in infectious diseases or clinical microbiology/virology. Testing for other pathogens, including flaviviruses such as dengue, JEV and Toscana viruses, should be considered according to the geographical area in which the infection is thought to have been acquired.

***Subgroups***

Returning travellers from outside of Europe can have encephalitis due to various pathogens, each of which may require bespoke testing protocols which should be supported by an expert in imported infections.

***Research Needs***

Updated direct evidence from larger studies designed to address this question is required to inform optimal specific tests, timing and samples for intrathecal antibody testing and its potential pitfalls.

***6. In adults with suspected infectious encephalitis and no aetiological diagnosis despite extensive investigation, who are deteriorating, does brain biopsy change patient management compared to no brain biopsy?***

***Good Practice Statement***

- Brain biopsy has no place in the initial assessment of suspected acute infectious encephalitis.
- Biopsy may inform diagnosis and can influence management when there is no aetiological diagnosis despite extensive investigation (including direct and indirect evidence for infectious and autoimmune causes as well as MRI and functional imaging such as PET), but the potential benefits should be balanced against the potential risks for the individual patient.
- Where possible, focal abnormalities on imaging should guide sampling using targeted stereotactic techniques.
- If imaging shows no focal abnormality, a blind biopsy may be considered.

***Justification***

Two suitable studies were identified [87, 88]. Brain biopsy has been proposed to improve the diagnosis of encephalitis in these smaller case series or cohort studies, some of which focus on specific types of encephalitis (e.g., brainstem). However, not all studies report on change in management and a comparison with those not having biopsy is not made in most studies making the evidence indirect and therefore requiring a good practice statement.

***Practical Notes***

The balance of benefit versus potential harm needs to be weighed carefully, and by experts in the procedure as well as the potential causes of the illness, so decision to perform brain biopsy should ideally be discussed in a multidisciplinary team. This should include at least an infection specialist, neurologist and neurosurgeon. This team should also decide on which tests should be performed on the brain tissue, which should be tailored to ongoing differential diagnoses, the type and amount of tissue obtained, which tests have already been performed, and the accessibility of investigations in local and reference laboratories.

***Subgroups***

Specific patient subgroups, for example those with immunocompromise or other comorbidities, may require a tailored approach to timing and indications for biopsy.

***Research Needs***

Studies seeking to assess the value of brain biopsy in the diagnosis of encephalitis should be conducted prospectively and focus on the impact on patient management.

### 4.3 Additional Investigations

***Background question: What are the typical CT and MRI abnormalities in infectious encephalitis?***

Patterns of imaging abnormalities are described in cohorts of more common encephalitis aetiologies (Table 7). HSV encephalitis typically exhibits bilateral asymmetrical involvement of the limbic system, including the medial temporal lobes, insular cortices and inferolateral frontal lobes and cingulate gyri, on MRI [93, 94]. In Japanese encephalitis, thalamic lesions on MRI are commonly seen, but the diagnosis is not excluded by their absence [89]. In TBE, a pathologic MRI is less common in the acute phase; however, with time, lesions may appear in thalami, basal ganglia and/or cerebellum [95].

**TABLE 7:** Key MRI features suggestive of specific viral causes.

Virus	Key MRI findings	Typical distribution
HSV‐1	T2 hyperintensity, restricted diffusion, sometimes haemorrhages are seen	Frontal and temporal lobes, rarely extratemporal
VZV	T2 hyperintensity with areas of restricted diffusion; vasculopathy, haemorrhage and necrosis may occur	Cerebellum, thalami, cerebral cortex, basal ganglia
CMV	Nonspecific T2/FLAIR periventricular hyperintensities and/or ventriculitis, more prominent in HIV‐associated cases	Periventricular, cerebral hemispheres
JEV	T2 hyperintensity, restricted diffusion. Sometimes haemorrhages are seen.	Thalami commonly involved. Basal ganglia, pons, midbrain, cerebellum are sometimes involved.
Dengue virus	T2 hyperintensity with areas of restricted diffusion. Sometimes petechial haemorrhages and diffuse cerebral oedema may be seen.	Bilateral thalami, pons, medulla
HIV	Diffuse cerebral atrophy. T2 hyperintensities in periventricular and deep white matter.	Periventricular and deep white matter

*Note:* References [89, 90, 91, 92].

Abbreviations: CMV, Cytomegalovirus; HIV, Human immunodeficiency virus; HSV, Herpes simplex virus; JEV, Japanese encephalitis virus; VZV, Varicella zoster virus.

***7. In adults with suspected encephalitis, what is the diagnostic accuracy of specific features on CT and MRI to predict an infectious aetiology compared to PCR?***

***Good Practice Statement***

- The diagnostic accuracy of brain imaging in predicting specific infectious aetiologies in encephalitis is unclear. It can be helpful to identify specific patterns of involvement of the brain on MRI to support suspicion of a particular pathogen pending PCR results.
- MRI (including contrast‐enhanced diffusion‐weighted imaging) is the preferred imaging modality and should be performed as soon as possible on patients with suspected infectious encephalitis; ideally this should be within 24 h of hospital admission, but certainly within 48 h.
- If the patient's condition precludes MRI scan, or MRI is not available, urgent CT scanning may help evaluate alternative causes of the patient's presentation or potential contra‐indications to immediate LP. However, the diagnostic accuracy of CT is not comparable to MRI for making a diagnosis of infectious encephalitis, and CT is less accurate than clinical features for predicting potential complications from a lumbar puncture.

***Justification***

Seven relevant studies were identified, none of which set out to make a direct comparison of the diagnostic accuracy of brain imaging versus PCR testing [27, 62, 63, 66, 67, 91, 96]. The presence of temporal lobe involvement in most patients with HSV encephalitis was reported in the majority of studies [27, 62, 63, 66, 67, 96]. The sensitivity and specificity of CT imaging for HSV was 83% and 100%, respectively, in a UK study; MRI had a sensitivity of 85% and specificity of 100% for HSV [27]. For JEV in India, the commonest feature on MRI was thalamic hyperintensity, brainstem involvement and basal ganglia involvement [91]. The included studies comprised a small number of participants, with substantial heterogeneity in terms of population and comparator diagnoses, so their results were not pooled using meta‐analysis and a recommendation was not made.

***Practical Notes***

Some features on an MRI scan of the brain may support continuation of therapy while awaiting PCR results, or when a PCR result is negative. For example, temporal lobe hyperintensity on MRI brain is suggestive of HSV encephalitis, so continuing aciclovir if the first PCR is negative, is advised. As CT is insensitive for establishing or excluding a diagnosis of encephalitis, it should not alone guide continuation or cessation of treatment. However, if an alternative diagnosis is established based on clinical features and CT, then cessation of presumptive encephalitis treatment can be considered (e.g., subdural haematoma in a patient on anticoagulation following a head injury), with the caveat that CT scans of patients with encephalitis may be reported as suggestive of an alternative pathology (e.g., ischaemic or haemorrhagic stroke or neoplasm). The timing of MRI can be of importance and repeated examinations might be necessary (especially if the patient continues to have features of encephalitis).

***Subgroups***

Patients may have potential alternative diagnoses that are better evaluated by CT imaging, or require urgent imaging, which may inform the decision on which modality to use.

***Research Needs***

Direct comparisons of brain imaging versus pathogen‐specific microbiological testing would inform relative accuracy of each.

***8. In patients with suspected encephalitis and psychiatric features, does electroencephalography (EEG) change the patient management compared to no EEG?***

***Good Practice Statement***

- The evidence is very uncertain about the impact of EEG on patient management. EEG need not be performed routinely in all patients with suspected infectious encephalitis.
- For patients with uncertainty whether there is a psychiatric or organic cause, an EEG should be performed to seek encephalopathic changes, as this may influence management.
- EEG should also be performed if subtle motor or nonconvulsive seizures are suspected.

***Justification***

One study reported that the majority of patients with probable limbic encephalitis of unknown aetiology (95%) referred from psychiatry for neurological assessment were diagnosed with organic rather than psychiatric disease following an abnormal EEG [97]. This was a small, single centre study from outside of Europe and did not delineate what change in management was due to EEG results versus other aspects of the neurological evaluation [97]. However, various diagnoses were made more likely by the EEG results; EEG is known to be a useful test to discriminate features of an organic encephalopathy from a psychiatric diagnosis [14, 98], and in suspected cases of non‐convulsive status epilepticus, encephalitis may be one of the most common causes [99].

***Practical Notes and Subgroups***

An abnormal EEG result should be interpreted in the context of the pre‐test probability of infectious encephalitis, as EEG does not distinguish adequately between infectious encephalitis and other types of encephalopathy for example autoimmune encephalitis and hepatic encephalopathy. Patients with suspected encephalitis in whom there is a clinical suspicion of non‐convulsive or subtle‐motor status epilepticus (Long & Koyfman's review of clinical features is helpful for non‐experts [100]) should be managed in clinical settings where EEG is available, ideally with capacity for continuous or prolonged monitoring. Patients at higher risk of seizures (e.g., HSV encephalitis, abnormal Glasgow Coma Scale, greater degree of temporal lobe oedema [101]), should be considered for transfer to settings with such capacity [102].

***Research Needs***

Larger prospective studies are needed to inform future recommendations on the use of EEG in patients with suspected encephalitis.

### 4.4 Treatment

***9. In patients with suspected encephalitis, does treatment with aciclovir improve patient outcome compared to no aciclovir administration?***

***Recommendation***

Upon suspicion of encephalitis, we recommend initiation of IV aciclovir as soon as possible (ideally within 6 h from presentation to hospital) (*Strong recommendation*; *low certainty in the evidence*). In some situations, other antimicrobial treatments should be considered alongside aciclovir (Table 8).

**TABLE 8:** Available treatments for common causes of encephalitis^a^.

Pathogen	Treatment
Viruses	
CMV	Ganciclovir or foscarnet
HIV	Antiretrovirals
HSV	IV aciclovir
VZV	IV aciclovir
Bacteria	
*Leptospira* species	Benzylpenicillin, ceftriaxone or doxycycline
*Listeria monocytogenes*	Ampicillin/amoxicillin, co‐trimoxazole or meropenem
*Mycobacterium tuberculosis*	Regimens vary, for drug‐susceptible disease most include rifampicin, isoniazid, pyrazinamide and ethambutol or a fluoroquinolone, with dexamethasone
*Orientia tsutsugamushi* (Scrub typhus)	Doxycycline and/or azithromycin
*Streptococcus pneumoniae* (pneumococcus, usually causes meningitis)	Ceftriaxone (+/− vancomycin, depending on local risk of ceftriaxone resistance)
*Treponema pallidum* (syphilis)	Penicillin G, procaine penicillin, ceftriaxone or doxycycline

Abbreviations: CMV, Cytomegalovirus; HIV, Human immunodeficiency virus; HSV, Herpes simplex virus; IV, Intravenous; VZV, Varicella zoster virus.

^a^ Adapted from [1, 69].

***Justification***

One eligible study was identified [103]. Data from an early randomised trial showed the superiority of aciclovir compared to vidarabine in terms of morbidity and mortality (19% vs. 50%, *p* = 0.04) for the treatment of HSV encephalitis [29, 103]. While randomised trials of aciclovir treatment have not been conducted for all patients with suspected encephalitis at presentation to hospital, the WG concluded that IV aciclovir should be started as soon as possible after suspicion of encephalitis based on HSV often being the most frequently reported aetiology of encephalitis in Europe [13]; high mortality and morbidity from HSV encephalitis [29, 104]; the benefit of early aciclovir administration in patients with HSV encephalitis [29, 104, 105, 106, 107, 108, 109, 110]; and aciclovir also being active against VZV [13, 96].

The TF agreed that the benefits of IV aciclovir administration far outweighed the risks; overall certainty in the evidence was moderate (as some of the evidence was indirect); and aciclovir is an easily affordable drug likely to be acceptable to patients, clinicians and policymakers and feasible to implement for patients with suspected encephalitis. Taking the other supporting rationale into account, the TF decided to adopt a recommendation made by previous guidelines, in line with GRADE‐ADOLOPMENT methodology [14, 39, 53, 54]. In doing so, the TF acknowledged limited evidence for the 6‐h target for initiating treatment, but felt this captured the balance of potential harm from delaying treatment against the practicalities of performing clinical assessment, evaluating for alternative common differential diagnoses and prescription and administration of an IV medication.

***Practical Notes***

The initial clinical picture may include other differential diagnoses. Thus, broad treatment should be initiated according to the individual situation until test results are available. Available treatments for specific causes of encephalitis are displayed in Table 8.

***Subgroups***

The recommendation also applies to immunocompromised patients. Pregnancy is not considered an absolute contraindication for aciclovir initiation.

***Research Needs***

There is interest in the potential use of high‐dose oral valaciclovir as an alternative to IV aciclovir, which requires further investigation [1, 111].

***9a. If HSV is not confirmed, when can aciclovir be stopped?***

***Good Practice Statements***

- If there is a low clinical suspicion of HSV encephalitis, or a confirmed alternative diagnosis, aciclovir can be stopped if ALL of the following apply: (1) negative CSF PCR for HSV (if LP was > 72 h after onset of neurological illness), (2) normal CSF white blood cell count (ignore if an alternative diagnosis would explain a raised count) and (3) no temporal lobe abnormality is found on MRI of the brain.
- If there is a high clinical suspicion of HSV encephalitis, aciclovir may be continued despite negative HSV PCR on CSF, the absence of temporal lobe encephalitis on brain MRI, and despite an alternative diagnosis being made. In such cases, re‐evaluation should be performed in consultation with an infection specialist and/or neurologist, including repeat CSF examination and brain imaging.

***Justification***

No study met the inclusion criteria, so a recommendation was not formulated. As HSV is a common cause of infectious encephalitis in adults in Europe (diagnosed in 10%–20% of those with suspected encephalitis) [6, 8, 112, 113], and HSV encephalitis can present with initial normal CSF leukocyte count, negative CSF HSV PCR and normal MRI brain appearances, it was decided that empirical treatment should only be stopped if all of these are present, when the clinical probability is felt to be low [22, 114, 115].

***Practical Notes***

Some PCR tests have suboptimal sensitivities and negative predictive values for HSV, so these may not be relied upon for meeting the above criteria for stopping treatment, as false‐negatives may be common [23].

***Subgroups***

The good practice statement may not apply universally, and specific subgroups may require an alternative approach, for example, immunocompromised patients and those with a differential diagnosis of VZV encephalitis (where aciclovir treatment may still be required).

***Research Needs***

Conditions for stopping aciclovir treatment should be evaluated and refined in prospective studies of patients with suspected HSV encephalitis receiving aciclovir.

***10. In patients with proven HSV encephalitis (positive CSF PCR or intrathecal antibody production), does administering aciclovir for more than 2 weeks reduce morbidity compared to 2‐week aciclovir therapy?***

***Good Practice Statements***

- In patients with proven HSV encephalitis, a minimum of 14 days of therapy with IV aciclovir should be administered.
- In patients who are improving, it is not necessary to repeat CSF examination before stopping therapy at 14 days.
- In patients who have not improved, are clinically worse, or who are immunocompromised, extension of therapy to 21 days or even 28 days, can be considered. In this case, repeat CSF examination for leukocyte count and HSV PCR may be of benefit, with continuation of aciclovir until those results are available.

***Justification***

One observational study of infectious encephalitis from France suggests longer durations of aciclovir (beyond 14 days) are not associated with improved patient outcomes [109]. Our ability to provide a decision regarding the effect of treatment duration is limited; however, due to the retrospective nature of the study with unmeasured confounding, and no adjustments for known confounders. No randomised trials have been conducted to compare durations of aciclovir therapy for HSV encephalitis. Early trials of aciclovir versus vidarabine used 10 days of aciclovir for HSV encephalitis, but relapses were later reported [29, 116, 117, 118]. Ongoing immune‐mediated inflammation is thought to drive most relapses, though viral replication can persist. As a result, most clinicians now use at least 14–21 days IV treatment in confirmed cases.

***Practical Notes***

If a patient is worsening despite IV aciclovir therapy for 14 days, alternative causes should be considered, such as co‐infection with another pathogen or non‐infective pathology including autoimmune. Since there is a risk of renal toxicity during aciclovir treatment, sufficient hydration is essential, and renal blood test monitoring should be performed at regular intervals.

***Subgroups***

Immunocompromised patients may require a different approach, as detailed in the good practice statements.

***Research Needs***

Prospective, preferably randomised, head‐to‐head comparisons of durations of aciclovir therapy should be conducted. Pharmacokinetic studies of aciclovir and its metabolites might help to inform the efficacy and safety of various durations, doses and formulations.

***10a. When should aciclovir resistance be suspected and what action should be taken?***

***Good Practice Statements***

- Aciclovir resistance in encephalitis is very rare and should not be assumed to be the cause of progressive disease.
- In a patient with progressive disease despite 14 days of IV aciclovir therapy, assessment of the likelihood of aciclovir resistance must be performed alongside exclusion of alternative causes of deterioration. Switch of therapy to foscarnet and testing for mutations in the HSV thymidine kinase gene may be considered, in consultation with a neurologist or infectious disease physician and a microbiologist/virologist.
- In patients who are immunosuppressed, and/or taking long‐term aciclovir prophylaxis, there may be a lower threshold for considering aciclovir resistance, though it is still considered to be rare in the context of encephalitis.

***Justification***

No studies were eligible for inclusion, though the body of indirect evidence available allowed a good practice statement to be formulated, which was deemed necessary to guide practice. Aciclovir resistance in encephalitis is described very rarely in the literature. Among non‐neurological isolates from immunocompetent patients who are not taking long‐term suppressive aciclovir therapy, resistance is also very rare [119, 120, 121]. Resistance, evidence for which comes mostly from non‐encephalitis patients, is mostly described in individuals who are immunosuppressed and/or taking long‐term suppressive or preventative aciclovir therapy [122].

***Practical Notes***

Phenotypic aciclovir resistance testing can be difficult to access, and there can be difficulties interpreting genotypic test results [123]. Turnaround times can also be long.

***Subgroups***

Immunocompromised patients may require a different approach, as detailed in the good practice statements.

***Research Needs***

Systematic analysis of HSV genomes recovered from CSF would be valuable in determining the absolute risk of resistance in patients with HSV encephalitis.

***11. In patients with proven HSV encephalitis, does the addition of corticosteroid therapy improve outcome compared to standard care alone?***

***11a. If yes, when should corticosteroids be introduced?***

***Recommendation***

For adults with proven HSV encephalitis, we are unable to recommend for or against the use of corticosteroids.

***Justification***

Effectiveness of corticosteroids has not been demonstrated, but harm from use of corticosteroids has also not been reported in trials, with moderate certainty in the evidence.

Three reports were eligible for inclusion [104, 124, 125]. A systematic review was deemed to be of low quality, so its results were not considered (Table S11.3) [124]. The German trial on Aciclovir and Corticosteroids in Herpes‐simplex‐virus Encephalitis (GACHE) terminated prematurely due to slow recruitment, but did not show any difference in outcome measures between adults with HSV encephalitis who received dexamethasone in addition to aciclovir compared to those who received aciclovir alone [104]. DexEnceph, a similar more recent trial in the UK, randomised 47 patients to aciclovir plus adjunctive dexamethasone and 47 to aciclovir alone, reporting no difference in primary or secondary outcomes between groups [125].

Further research may inform updates to this recommendation in the future. The corresponding Section 11 in Appendix S1 provides the full Evidence to Decision documentation which underpins the decision. The summary of findings table (Table S11.2) contains only the primary outcome results for each trial (GACHE and DexEnceph), as no significant difference was reported for any of the many secondary outcomes in either trial. The results of the two trials were not combined. The DexEnceph trial report was just published, and it was decided that a potentially premature meta‐analysis would be unlikely to provide evidence for benefit or harm, as none of the outcomes had substantially different results between the groups in each trial.

***Practical Notes***

Currently, in patients with suspected encephalitis where the aetiology may be HSV, another infection or an autoimmune cause, corticosteroids are not usually given until HSV has been excluded. However, the evidence now suggests that corticosteroid therapy given as part of empirical treatment for other suspected brain infections such as bacterial meningitis and CNS tuberculosis, or for suspected autoimmune encephalitis, is unlikely to be harmful in patients ultimately diagnosed with HSV encephalitis.

***Subgroups***

No subgroup analyses were performed in the German trial. Predefined subgroup analyses did not demonstrate the benefit of corticosteroid use versus placebo within the UK‐based trial.

***Research Needs***

Future research should focus on the effect of early administration of corticosteroids alongside aciclovir therapy, compared against aciclovir therapy without corticosteroids. This was not fully addressed by the UK trial, in which there was a median of 7 days from presentation to starting corticosteroid therapy. This trial also reported in a post hoc analysis, among patients receiving dexamethasone, that deterioration in the primary outcome (the Wechsler memory score) was associated with a delay in administration of dexamethasone [125].

***12. In patients with proven VZV encephalitis (positive CSF PCR or intrathecal antibody production), does treatment with high dose aciclovir (usually 20 mg/kg three times per day) compared to 10 mg/kg aciclovir three times per day reduce mortality and morbidity?***

***Good Practice Statement***

Patients with proven VZV encephalitis should be treated with a standard dose of 10 mg/kg aciclovir three times daily (if renal function is normal). If an associated vasculitis is confirmed, corticosteroid therapy should be added; in the absence of evidence of vasculitis, empirical corticosteroids should not be started for this indication.

***Justification***

No comparative studies investigating the use of 20 mg/kg per dose of aciclovir were identified, thus there is insufficient evidence for a recommendation to be made regarding dosing of aciclovir in VZV encephalitis. The WG agreed to a standard dose of 10 mg/kg based on lack of evidence of potential benefit but well‐established potential harm. Aciclovir has several potential adverse effects, including renal failure, which may be more common with higher doses [126, 127]. When radiological evidence of CNS vasculitis in patients with VZV CNS infection is detected, corticosteroids might be considered, in addition to antiviral treatment, in accordance with treatment of other CNS vasculitides [98]. While corticosteroids are used widely to treat post‐infectious vasculitis [98], there is no evidence of benefit for patients with VZV encephalitis without vasculitis.

***Practical Notes and Subgroups***

The suggested dosing also applies to immunocompromised individuals. If renal insufficiency exists or develops, aciclovir dosing will require adjustment [127]. Concentrations of aciclovir and the metabolite 9‐carboxymethoxymethylguanine, which correlates with toxicity symptoms, can be measured in serum and CSF [128, 129]. There are no head‐to‐head studies comparing corticosteroid doses for vasculitis; thus, specific steroid dosing regimens remain unclear.

***Research Needs***

Comparative studies are needed to inform optimal dosing of aciclovir therapy in VZV encephalitis.

***13. What is the impact on morbidity and mortality of prophylactic antiseizure treatment versus no prophylactic antiseizure treatment in cases of infectious encephalitis?***

***Good Practice Statements***

- Evidence for use of prophylactic anti‐seizure medication in patients with infectious encephalitis is lacking. From the available indirect evidence, the WG concluded that the balance between benefit and potential harm is *against* starting primary prophylaxis (without seizures as part of the illness).
- Management of seizures in the acute phase should be as per existing guidelines.
- A decision to continue the anti‐seizure medication after the acute hospital admission should be individualised based on various factors, including the extent and location of abnormalities on brain imaging and the number and timing of seizures and should be made in discussion with the patient and the multidisciplinary team.

***Justification***

No studies were eligible for inclusion, so a recommendation could not be made. However, two cohort studies have shown that the number of anti‐seizure drugs was not significantly associated with terminal seizure freedom after the acute phase [130] and the risk of seizure relapse did not differ between patients on anti‐seizure drugs and those without [131].

***Practical Notes***

The decision to continue/stop will depend on whether seizures are prolonged; whether seizures are recurrent; whether there are large areas of abnormality on MRI scan of the brain; whether specific areas are affected; and the results of EEG. The anti‐seizure medication administered should be chosen as per usual epilepsy management guidelines.

***Subgroups***

The conclusions made by the WG also apply to specific subgroups, including immunocompromised patients.

***Research Needs***

Studies should evaluate the benefits versus harms of primary prophylactic anti‐seizure medication and the optimal timing of stopping anti‐seizure medication post‐encephalitis.

### 4.5 Ongoing Care and Rehabilitation

***14. In patients with infectious encephalitis and deep/worsening coma score or status epilepticus, does admission to intensive care improve outcome and reduce mortality compared to no admission to intensive care?***

***Good Practice Statement***

Patients with infectious encephalitis and falling level of consciousness or status epilepticus (including non‐convulsive status epilepticus) require admission to an intensive care unit (ICU) or a neuro‐critical care unit, if available, for assessment for airway protection and ventilatory support; management as needed of blood pressure, intracranial pressure and cerebral perfusion pressure (using invasive monitoring where appropriate); and correction of metabolic imbalances, including electrolytes, carbon dioxide and glucose.

***Justification***

One retrospective cohort study of consecutive adult patients admitted with HSV encephalitis reported that ICU admission was not associated with an unfavourable outcome at discharge in multivariate analysis when adjusting for other potential prognostic indicators, though its retrospective nature meant that it was not deemed suitable to inform a recommendation directly [108]. Relevant indirect evidence exists from other causes of coma and other neurological conditions (e.g., bacterial meningitis, stroke) which supports the use of critical care [132, 133, 134].

***Practical Notes and Subgroups***

The correction of simple precipitating factors, e.g., hypo−/hyperglycaemia or hypoxia or hypercapnia, should be attempted before planning transfer to ICU or started if ICU care is needed anyway. Although most patients with infectious encephalitis and falling level of consciousness or status epilepticus require admission to an ICU, it should be noted that some patients may present with situations making ICU transfer less desirable, for example, those at a terminal stage of another disease at the time of onset of encephalitis. The decision for ICU care should be made through discussion between the treating clinicians, ICU team and patient/relatives.

***Research Needs***

Prospective studies including patients with a variety of causes of infectious encephalitis could help strengthen the evidence in this area.

***15. In a patient with HSV encephalitis with recurrent/worsening symptoms, does autoantibody testing improve the chance of identifying the cause of relapse compared to not testing for autoantibodies?***

***Good Practice Statement***

In a patient with recent HSV encephalitis with recurrent or worsening symptoms, CSF should be tested for anti‐N‐methyl‐D‐aspartate (anti‐NMDA) receptor antibodies. Testing for other autoantibodies should also be considered.

***Justification***

No studies were eligible for inclusion; the lack of direct evidence precluded formulation of a recommendation. Other studies have found that a significant proportion (27%–57%) of patients with relapsing symptoms post‐HSV encephalitis have autoantibodies, predominantly anti‐NMDA receptor antibodies [135, 136, 137].

***Practical Notes***

The WG considered the few reports of other autoantibodies (apart from anti‐NMDA receptor antibodies) that can be responsible for worsening or recurrent symptoms in patients with recent HSV encephalitis, including GAD65 and Caspr2 [138, 139]. Since the body of literature is limited, exactly which other antibodies to test for should not be specified at this point, and this decision should be taken by a specialist, usually a neurologist. This may change as further knowledge accumulates.

***Subgroups***

There is insufficient evidence to inform how this good practice statement may apply differently to specific subgroups.

***Research Needs***

There is a need for large prospective studies of patients with recurrent or worsening symptoms after HSV encephalitis, with systematic application of a variety of autoantibody and other tests.

***16. In patients with infectious encephalitis, does follow‐up care and rehabilitation reduce morbidity compared to no follow‐up care and rehabilitation?***

***Good Practice Statements***

- All patients with encephalitis should receive periodic assessment and rehabilitation at each stage of their encephalitis journey, from acute presentation to their return to the community. This should be tailored to the patient, in terms of their stage and severity of illness and its impact, and to their goals, values and preferences.
- Patients (when conscious level permits) and their caregivers should be made aware of the support provided by the health and social care system of their country of residence, and also by non‐governmental organisations such as Encephalitis International (www.encephalitis.info).
- Patients (and caregivers) should not be discharged from hospital without communication about their diagnosis and its potential impact on their life. Arrangements for outpatient follow‐up and plans for ongoing therapy and rehabilitation should be formulated prior to hospital discharge.
- All patients should be given access to assessment for rehabilitation.

***Justification***

Two studies were included [140, 141]. A systematic review reported a potential beneficial effect of rehabilitation interventions in a variety of settings among paediatric and adult patients with infectious encephalitis, though only small studies with low methodological quality were included [140]. The other study conducted narrative interviews of people affected by HSV encephalitis and their caregivers, concluding that focused rehabilitation facilitated a more supported transition between the hospital and home [141]. While the evidence did not directly address morbidity outcomes, a good practice statement was considered necessary, as the benefit of rehabilitation following similar illnesses far outweighs the potential harms and opportunity costs [142, 143, 144].

***Practical Notes***

Neurorehabilitation should be started as early as possible, even if the patient is not capable of engaging with this actively, to avoid sequelae from loss of neurological function. While patients with any critical illness benefit from rehabilitation, neurorehabilitation is preferred for patients with encephalitis. Neurorehabilitation teams integrate physical, cognitive and behavioural issues and have more experience in specific complications of injury to the brain such as epilepsy and painful hypertonia of limbs.

Patients and caregivers (i.e., person who provides regular support to someone who has an illness, disability or long‐term condition and needs help with daily life or health‐related needs) should be involved in formulation of the rehabilitation objectives. Care should be taken to ensure that all patients with encephalitis should have an assessment for rehabilitation, even if they are determined to have mild disease or full recovery. Some needs may be subtle or masked, and the patient's perspective on impact of their illness on their life may differ from that of healthcare professionals.

***Subgroups***

The majority of studies focus on HSV encephalitis, limiting generalisability to all patients with encephalitis.

***Research Needs***

Prospective studies of rehabilitation following encephalitis should be conducted, informed by work to determine patient‐reported outcome measures and core outcome sets for post‐encephalitis rehabilitation, including morbidity, quality of life and functional outcomes.

## 5 Discussion

While prevention of encephalitis through targeted vaccination against infections such as measles and JEV is critical, these clinical practice guidelines focus on diagnosis and management. Recommendations and good practice statements are provided based on current evidence. It is hoped that this will help improve diagnostic and management practices for infectious encephalitis across Europe. Given the clinical severity and public health importance of this condition, the paucity of high‐quality evidence is striking. Transnational, large‐scale research in infective encephalitis is encouraged. Research should focus on novel treatments and diagnostics, the development of a core outcome set for use in future trials of interventions for acute care and post‐encephalitis rehabilitation, and other identified areas that have been highlighted under the relevant questions in this guideline. The search strategy for the guideline was limited to two databases for practical reasons; however, it is unlikely that important papers were missed as task force experts are also familiar with relevant literature. This guideline summarises the available evidence and provides a comprehensive set of statements for use by clinicians, laboratory specialists and policymakers to inform decisions around diagnosis and management of suspected and confirmed infectious encephalitis in adults.

## Author Contributions

**Julia Granerod:** methodology, formal analysis, investigation, data curation, writing – original draft, supervision, project administration, writing – review and editing. **Johann Sellner:** formal analysis, writing – review and editing. **Jean‐Paul Stahl:** formal analysis, writing – review and editing. **Sylviane Defres:** formal analysis, writing – review and editing. **Alexandra Mailles:** formal analysis, writing – review and editing. **Erich Schmutzhard:** formal analysis, writing – review and editing. **Elisabeth Aurelius:** formal analysis, writing – review and editing. **Thomas Whitfield:** formal analysis, funding acquisition, writing – review and editing. **Luca Massacesi:** formal analysis, writing – review and editing. **Maarten J. Titulaer:** formal analysis, writing – review and editing. **Pille Taba:** formal analysis, writing – review and editing. **Benedict D. Michael:** formal analysis, writing – review and editing. **Tehmina Bharucha:** data curation, writing – review and editing. **Lance Turtle:** data curation, writing – review and editing. **Brendan Sargent:** data curation, writing – review and editing. **Arina Tamborska:** data curation, writing – review and editing. **Jonathan Cattrall:** data curation, writing – review and editing. **Yun Huang:** data curation, writing – review and editing. **Eva Maria Hodel:** data curation, writing – review and editing. **Suzie Lant:** data curation, writing – review and editing. **Glynn Webb:** data curation, writing – review and editing. **Rajish S. K. Shil:** data curation, writing – review and editing. **Shalley Dhar:** data curation, writing – review and editing. **Diederik van de Beek:** formal analysis, writing – review and editing. **Marie Studahl:** formal analysis, writing – review and editing. **Matthijs C. Brouwer:** formal analysis, writing – review and editing. **Uta Meyding‐Lamade:** formal analysis, writing – review and editing. **Ava Easton:** formal analysis, writing – review and editing. **Bhagteshwar Singh:** methodology, formal analysis, investigation, writing – original draft, supervision, writing – review and editing. **Tom Solomon:** conceptualization, methodology, supervision, funding acquisition, writing – review and editing.

## Funding

The development of this guideline was funded jointly by ESCMID and EAN. EAN and ESCMID approval of the guideline was received on 13 and 28 November, 2025, respectively.

## Conflicts of Interest

EAN Register of Interest forms were collected from all TF members in May 2020 and submitted to the EAN in June 2020. ESCMID Conflicts of Interest (COI) forms were collected also from TF members in 2020. Updated forms were collected upon submission of the draft manuscript in March 2025. For each WG, COI forms were reviewed by the guidelines management team: T.S., J.G. and B.S. (methodologist). For each potential COI, the Guidelines International Network principles were followed [145]. Specifically, all WG and TF members within the meetings were made aware of COIs relevant to the question being discussed, so that this was taken into account when that member spoke. It was not necessary to prevent voting on any recommendation for a specific COI. Data extraction and risk of bias/quality assessment were not performed by a group member who had been an author on a given study.

## Supporting information


**Figure S1:1.** Flow chart for study inclusion for PICO 1.
**Table S1:1** Characteristics of included studies for PICO 1.
**Table S1:2** Presence of seizures as a presenting feature of HSV among included studies.
**Table S1:3** Presence of seizures and fever as a presenting feature of VZV among included studies.
**Figure S1:2** Pooled sensitivity and specificity of seizures for the identification of HSV encephalitis.
**Figure S1:3** Pooled sensitivity and specificity of fever for the identification of HSV encephalitis.
**Figure S1:4** Sensitivity and specificity of altered behaviour for the identification of HSV encephalitis.
**Table S1:4** Quality assessment of included diagnostic accuracy studies using QUADAS‐2.
**Figure S2:1** Flow chart for study inclusion for PICO 2.
**Table S2:1** Characteristics of included studies for PICO 2.
**Table S2:2** Sensitivity, specificity, NPV and PPV of age, sex and preceding URTI as predictors of HSV encephalitis.
**Table S2:3** Sensitivity, specificity, NPV and PPV of age as a predictor of VZV encephalitis.
**Table S2:4** Quality assessment of included diagnostic accuracy studies using QUADAS‐2.
**Figure S3:1** Flow chart for study inclusion for PICO 3/3a.
**Table S3:1** Characteristics of included studies for PICO 3a.
**Figure S4:1** Flow chart for study inclusion for PICO 4.
**Figure S5:1** Flow chart for study inclusion for PICO 5/5b.
**Figure S5:2** Flow chart for study inclusion for PICO 5a.
**Table S5:1** Characteristics of included studies for PICOs 5/5b and 5a.
**Table S5:2** Question: Should BioFire FilmArray ME be used to diagnose HSV‐1 infection in patients with suspected encephalitis?
**Table S5:3** Question: Should BioFire FilmArray ME be used to diagnose HSV‐2 infection in patients with suspected encephalitis?
**Table S5:4** Question: Should BioFire FilmArray ME be used to diagnose VZV infection in patients with suspected encephalitis?
**Table S5:5** Question: Should BioFire FilmArray ME be used to diagnose enterovirus infection in patients with suspected encephalitis?
**Table S5:6** Quality assessment of included diagnostic accuracy studies using QUADAS‐2.
**Table S5:7** Quality assessment of Trujillo‐Gomez et al. using PRISMA checklist.
**Figure S6:1** Flow chart for study inclusion for PICO 6.
**Table S6:1** Characteristics of included studies for PICO 6.
**Table S6:2** Quality assessment of included diagnostic accuracy studies using JBI case series.
**Figure S7:1** Flow chart for study inclusion for PICO 7.
**Table S7:1** Characteristics of included studies for PICO 7.
**Figure S8:1** Flow chart for study inclusion for PICO 8.
**Table S8:1** Characteristics of included studies for PICO 8.
**Table S8:2** Quality assessment of Chandra et al. using Newcastle Ottawa scale for cross‐sectional studies.
**Figure S9:1** Flow chart for study inclusion for PICO 9 and 9a.
**Table S9:1** Characteristics of included studies for PICO 9.
**Table S9:2** Quality assessment of Hjalmarsson/Skoldenberg using revised Cochrance risk‐of‐bias tool for randomised trials (RoB 2).
**Figure S10:1** Flow chart for study inclusion for PICO 10 and 10a.
**Table S10:1** Characteristics of included studies for PICO 10.
**Table S10:2** Quality assessment of study using ROBINS‐I assessment tool.
**Figure S11:1** Flow chart for study inclusion for PICO 11 and 11a.
**Table S11:1** Characteristics of included studies for PICO 11.
**Table S11:2** Corticosteroid therapy compared to no corticosteroid therapy for adults with proven HSV encephalitis.
**Table S11:3** Methodological quality of Ramos‐Estebanes et al. systematic review using AMSTAR 2 assessment tool.
**Table S11:4** Risk of bias assessment of Meyding‐Lamade et al. and Solomon et al. study using ROB 2 assessment tool.
**Figure S12:1** Flow chart for study inclusion for PICO 12.
**Figure S13:1** Flow chart for study inclusion for PICO 13.
**Figure S14:1** Flow chart for study inclusion for PICO 14.
**Table S14:1** Characteristics of included studies for PICO 14.
**Table S14:2** Quality assessment of Singh et al. using Newcastle Ottawa scale for cross‐sectional studies.
**Figure S15:1** Flow chart for study inclusion for PICO 15.
**Figure S16:1** Flow chart for study inclusion for PICO 16.
**Table S16:1** Characteristics of included studies for PICO 16.
**Table S16:2** Quality assessment of Christie et al. study using AMSTAR 2 assessment tool.
**Table S16:3** Quality assessment of Cooper et al. using JBI critical appraisal checklist for qualitative research.

## Data Availability

The data that support the findings of this study are available in Appendix S1 of this article. These data were derived from the following resources available in the public domain: PubMed at https://pubmed.ncbi.nlm.nih.gov/ and the Cochrane Central Register of Controlled Trials at https://www.cochranelibrary.com/central/about‐central.
