# Supplementary material for: Diagnosis and Management of Infectious Encephalitis in Adults: Guidelines From the European Academy of Neurology and European Society of Clinical Microbiology and Infectious Diseases
Source: Eur J Neurol. 2026 Oct 9;33(10):e70647. doi: 10.1111/ene.70647 (PMC13650609; doi:10.1111/ene.70647)
Supplement: Supplementary file 1 [file ENE-33-e70647-s001.pdf]

Supplementary material

EAN/ESCMID guideline on the diagnosis and management of encephalitis in adults caused by infection

Contents

Study protocol 5

Appendix 1. PICO questions 15

Appendix 2. Search strategies 18

1. In adults with suspected encephalitis, how accurate are presenting clinical features in predicting an infectious aetiology compared to PCR? 22

- Evidence summary 22
- Search string 22
- Flowchart 23
- Characteristics of included studies 24
- Forest plots 27
- Quality assessment 30

2. In adults with infectious encephalitis, does the presence of risk factors (i.e., immune status, travel history, infectious contacts, pets, water exposure, past medical history) compared to their absence result in a higher risk of specific infectious aetiologies (as confirmed by PCR)? 31

- Evidence summary 31
- Search string 31
- Flowchart 32
- Characteristics of included studies 33
- Quality assessment 36

3. In adults with suspected encephalitis, does LP before imaging result in deterioration or worse clinical outcome compared to no LP before imaging? 37

3a. What are the contraindications for an immediate LP? 37

- Evidence summary 37
- Search string 37
- Flowchart 38
- Characteristics of included studies 39

4. In adults with suspected encephalitis and a first non-diagnostic LP, does a second LP 48 hours later result in improved aetiological diagnosis? 40

- Evidence summary 40
- Search string 40
- Flowchart 41

5/5b. In suspected sporadic infectious encephalitis is individualised/specific CSF PCR better than standardised/multiplex CSF PCR panel for first-line detection of the causative pathogen? Are there additional specific testing panels for immunocompromised patients? 42
5a. Which CSF antibody testing is useful in the diagnosis of infectious encephalitis? 42
Evidence summary 42
Search string 43
Flowchart 44
Characteristics of included studies 46
Summary of findings tables 47
Quality assessment 55
6. In adults with suspected infectious encephalitis and no aetiological diagnosis despite extensive investigation, who are deteriorating, does brain biopsy change patient management compared to no brain biopsy? 59
Evidence summary 59
Search string 59
Flowchart 60
Characteristics of included studies 61
Quality assessment 61
7. In adults with suspected encephalitis, what is the diagnostic accuracy of specific features on CT and MRI to predict an infectious aetiology compared to PCR? 62
Evidence summary 62
Search string 63
Flowchart 63
Characteristics of included studies 64
Quality assessment 65
8. In patients with suspected encephalitis and psychiatric features, does EEG change the patient management compared to no EEG? 66
Evidence summary 66
Search string 66
Flowchart 67
Characteristics of included studies 68
Quality assessment 69
9. In patients with suspected encephalitis, does treatment with acyclovir improve patient outcome compared to no acyclovir administration? 70
9a. If HSV is not confirmed when can acyclovir be stopped? 70
Evidence summary 70
Search string 70

Flowchart ... 71
Characteristics of included studies ... 72
Quality assessment ... 73
10. In patients with proven HSV encephalitis (positive CSF PCR or intrathecal antibody production), does administering aciclovir for more than two weeks reduce morbidity compared to two-week aciclovir therapy? ... 74
10a. When should aciclovir resistance be suspected and what action should be taken? ... 74
Evidence summary ... 74
Search string ... 75
Flowchart ... 75
Characteristics of included studies ... 76
Quality assessment ... 77
11. In patients with proven HSV encephalitis, does the addition of corticosteroid therapy improve outcome compared to standard care alone? ... 78
11a. If yes, when should steroids be introduced? ... 78
Evidence summary ... 78
Search string ... 78
Flowchart ... 79
Characteristics of included studies ... 80
Summary of findings tables ... 82
Quality assessment ... 83
12. In patients with proven VZV encephalitis (positive CSF PCR or intrathecal antibody production), does treatment with high dose aciclovir (usually 20 mg/kg three times per day) compared to 10 mg/kg aciclovir three times per day reduce mortality and morbidity? ... 85
Evidence summary ... 85
Search string ... 85
Flowchart ... 86
13. What is the impact on morbidity and mortality of prophylactic antiseizure treatment versus no prophylactic antiseizure treatment in cases of infectious encephalitis? ... 87
Evidence summary ... 87
Search string ... 87
Flowchart ... 88
14. In patients with infectious encephalitis and/or deep worsening coma score or status epilepticus, does admission to intensive care improve outcome and reduce mortality compared to no admission to intensive care? ... 89
Evidence summary ... 89
Search string ... 89
Flowchart ... 90

Characteristics of included studies 91
Quality assessment 92
15. In a patient with HSE with relapse, does autoantibody testing improve the chance of identifying the cause of relapse compared to not testing for autoantibodies 93
Evidence summary 93
Search string 93
Flowchart 94
16. In patients with infectious encephalitis, does follow-up care and rehabilitation reduce morbidity compared to no follow-up care and rehabilitation? 95
Evidence summary 95
Search string 95
Flowchart 96
Characteristics of included studies 97
Quality assessment 98
References 100

Study protocol

1. Background

Encephalitis, or inflammation of the brain, is a syndrome of multiple pathogeneses and aetiologies. Direct infection of the central nervous system is generally considered the most common pathogenic mechanism (i.e., infectious encephalitis); however, encephalitis can also be immune-mediated. More than 100 different organisms have been recognised as causative agents.

Encephalitis is a life-threatening condition associated with significant morbidity and mortality, and survivors are often left with serious ongoing sequelae [1-3]. The global incidence of encephalitis in western settings varies but is estimated to be between 0.7 and 12.6 per 100,000 per year for adults, with herpes simplex virus (HSV) the most common aetiology (1 in 250,000) [4]. Encephalitis poses a significant financial burden with herpes encephalitis alone estimated to result in >10,000 hospital bed days annually in the United Kingdom (UK), 45% of sufferers unable to return to work, and an estimated cost of >$50,000 per case in the United States [5,6].

Encephalitis is difficult to diagnose, investigate, and manage and thus presents a challenge to clinicians. European guidelines will help to inform timely treatment choices and optimise patient care. The diagnostic challenge in encephalitis occurs in part due to nonspecific presenting symptoms including fever and altered mental state; only a minority of those initially suspected have encephalitis [7,8]. Encephalitis should be considered a differential diagnosis as failure to consider the condition and instigate treatment is both common and associated with worse patient outcome [9,10]. Guidance on when it is appropriate to consider and treat encephalitis as part of first line assessment would be beneficial.

Once a diagnosis of encephalitis is established, further uncertainty can result from the myriad of possible aetiologies which are not immediately distinguishable and have different treatments [11,12]. The aetiology remains unexplained in 21% to 72.5% of cases [13]. Guidelines are imperative for the development of a standardised investigative protocol, giving clinicians validity in their approach, awareness of potential second line investigations, and treatment options to pursue.

A clinician is also hampered by the equivocal nature of some tests. Repeat lumbar puncture (LP) is necessary for some diagnoses, and polymerase chain reaction (PCR) and antibody tests must be performed and interpreted correctly. Antibody tests for West Nile virus (WNV) and tick-borne encephalitis virus (TBEV) for example are nonspecific and uncertainty may arise from antibody cross reaction [14,15]. A standardised approach to investigation of the encephalitic patient in general is required to optimise diagnostic certainty as far as possible.

Treatment for encephalitis is dependent on the underlying cause. Common causes of encephalitis such as HSV and varicella zoster virus (VZV) can be treated effectively with aciclovir [1]; however, uncertainty remains regarding aciclovir duration and the necessity of

viral clearance from the cerebrospinal fluid (CSF) [12,16]. There is also significant interest in the benefit of immunomodulation with steroid therapy in herpes simplex encephalitis (HSE) [17,18]. Other infectious aetiologies have no proven treatment; therapies such as interferon, ribavirin, immunoglobulin, and steroids have been trialled with uncertain efficacy [19-23]. A robust review of and consensus opinion regarding these potential treatments would be valuable for clinicians dealing with this condition.

The most recent European and UK-specific guidelines were published in 2010 and 2012, respectively [24,25]. Over the last decade; however, the epidemiology has changed across Europe with increasing prevalence of people living with human immunodeficiency virus (HIV), increased incidence of syphilis, and a decline in rabies cases [26-28]. Newly emergent viruses, such as Zika and severe acute respiratory syndrome coronavirus 2 (SARS-CoV-2), have been shown to sporadically result in encephalitis [29,30]. Flavivirus transmission (WNV and TBEV) has increased across Europe over the past decade with a concurrent rise in encephalitis cases [31,32]. Increases in these conditions are projected to continue and guidelines will need to account for this. In addition, technological advances in diagnostics have been made over the last decade and new guidance is needed to reflect these developments.

The European Academy of Neurology (EAN) Scientific Panel (SP) on Infectious disease, along with the European Society of Clinical Microbiology and Infectious Diseases (ESCMID), has appointed a Task Force to develop guidelines on the diagnosis and management of infectious encephalitis. The main purpose is to produce recommendations for the diagnosis and management of infectious encephalitis in adults based on current evidence and expert opinion.

The EAN SP of Neuroimmunology is working on guidelines for the management of autoimmune encephalitis. As a result of overlap between the two topics (particularly in the initial approach to patients presenting with an "encephalitis syndrome"), the two groups will collaborate to ensure a complimentary diagnostic and treatment algorithm and to avoid discrepancies.

2. Previous guidelines

Despite the availability of established protocols for the diagnosis of common causes of encephalitis, there is limited evidence available in terms of the clinical picture for novel causes of encephalitis including Ebola and Zika viruses. There is conflicting evidence regarding the benefit of corticosteroids for HSE and TBE [18,23]. Interferon alpha has been shown to be beneficial in single cases and small case series of patients with WNV encephalitis; however, when interferon was administered during the 2000 WNV outbreak in Israel no benefit was recorded [20,21]. Critically, there are no current European guidelines to advise on navigating a suspected encephalitis case factoring in these uncertainties. The latest European guidelines were published in 2010 [24], and UK guidelines were published in 2012 (**Table 1**) [25]. Since this time, novel causes of encephalitis have emerged and this, along with diagnostic processes, needs to be reflected in current guidance.

Table 1. Existing guidelines with relevance to present EAN/ESCMID Guideline

Ref	Year	Title	Published/endorsed by	Description
24	2010	Viral meningoencephalitis: A review of diagnostic methods and guidelines for management	European Federation of Neurological Sciences	addresses the optimal clinical approach to CNS infections caused by viruses<br>Addresses optimal clinical approach to viral meningoencephalitis
25	2012	Management of suspected viral encephalitis in adults	Association of British Neurologists and British Infection Association National Guidelines	Written to aid early recognition and appropriate investigation and management of patients with suspected encephalitis

3. Objectives

This Guideline was proposed at a meeting of the Scientific Panel of the Encephalitis Society in 2018. The aim is to provide the European neurological community with state-of-the-art evidence regarding diagnosis and management of infectious encephalitis in adults.

4. Scope

The scope of this Guideline is to cover the diagnosis and management of infectious encephalitis, including when to suspect infectious encephalitis, microbiological investigations, additional investigations (including neuroimaging and electroencephalogram [EEG]), treatment, and ongoing care and rehabilitation. The target population includes all adults, including those who are immunocompromised. All infectious aetiologies of encephalitis will be considered with some questions focussed on specific causes (e.g., aciclovir in HSV). The target audience and intended users of this Guideline include all clinicians involved in diagnosis, treatment, and follow-up of encephalitis in adults in the context of hospital care, including infectious disease specialists, neurologists, intensive care specialists, and microbiologists.

5. Task Force Members

A Task Force has been convened for this purpose and includes a Chair, Senior coordinating advisor, two chairs of the EAN SP on Infectious Diseases, two representatives of the EAN SP on Neuroimmunology, 12 other experts/advisors in the field of encephalitis, and one methodologist. The Task Force includes nine EAN members, six ESCMID members, and three members of both organizations. One representative of a patient organization (i.e., The Encephalitis Society) who is also an expert/advisor in the field of encephalitis, is included in the Task Force. Geographical representation of the Task Force was sought with representatives from Western, Eastern, Northern, and Southern Europe.

The Task Force Members include:

Tom Solomon (Chair and Professor of Neurology), Department of Clinical Infection, Microbiology & Immunology, Institute of Infection and Global Health, University of Liverpool, Liverpool, UK

Julia Granerod (Senior Coordinating Advisor), Department of Clinical Infection, Microbiology & Immunology, Institute of Infection and Global Health, University of Liverpool, Liverpool, UK
Bhagteshwar Singh (Methodologist), Department of Clinical Infection, Microbiology & Immunology, Institute of Infection and Global Health, University of Liverpool, Liverpool, UK
Pille Taba (EAN SP Infectious Diseases), Department of Neurology, University of Tartu, Tartu, Estonia
Johann Sellner (EAN SP Infectious Diseases), Department of Neurology, Landesklinikum Mistelbach, Mistelbach, Austria
Ava Easton (Patient representative and subject expert), Encephalitis International (formerly The Encephalitis Society), Malton, UK
Jean-Paul Stahl, Infectious Diseases Department, University Hospital Grenoble, Grenoble, France
Benedict Michael, Department of Clinical Infection, Microbiology & Immunology, Institute of Infection and Global Health, University of Liverpool, Liverpool, UK
Sylviane Defres, Department of Clinical Infection, Microbiology & Immunology, Institute of Infection and Global Health, University of Liverpool, Liverpool, UK
Alexandra Mailles, Department of Epidemiology, French National Public Health Agency, Saint Maurice, France
Diederik van de Beek, Department of Neurology, University of Amsterdam, Amsterdam, Netherlands
Erich Schmutzhard, Department of Neurology, Medical University Innsbruck, Innsbruck, Austria
Marie Studahl, Department of Infectious Diseases, University of Gothenburg, Gothenburg, Sweden
Elisabeth Aurelius, Division of Infectious Diseases, Department of Medicine, Karolinska Institute, Stockholm, Sweden
Uta Meyding-Lamade, Department of Neurology and Epileptology, Hospital Nordwest Frankfurt, Frankfurt, Germany
Matthijs Brouwer, Department of Neurology, University of Amsterdam, Amsterdam, Netherlands
Thomas Whitfield, Department of Clinical Infection, Microbiology & Immunology, Institute of Infection and Global Health, University of Liverpool, Liverpool, UK
Maarten Titulaer (EAN SP Neuroimmunology), Department of Neurology, Erasmus University Medical Center, Rotterdam, Netherlands
Luca Massacesi (EAN SP Neuroimmunology), Department of Neurology, University of Florence, Florence, Italy

6. Clinical questions

The proposed procedure for guideline development is in accordance with EAN practical recommendations for proposing, planning, and writing neurological management guidelines and ECSMID guidance for clinical practice guidelines [33,34]. Possible topics and questions to be addressed by the Guidelines were identified from previous published guidelines and circulated to the Task Force ahead of the first virtual meeting [25]. A meeting of the Task Force was held via Zoom on 7th September 2020. These questions were discussed in break-out groups at the meeting and brought back to the whole group for agreement. Key

questions were agreed by consensus and formulated in PICO (Population, Intervention, Control, Outcome) format as recommended by the EAN [33]. Using the PICO approach, we have phrased the following questions (for more detail and definition of outcomes see **Appendix 1**):

A. WHEN TO SUSPECT ENCEPHALITIS
- *PICO1*: In adults with suspected encephalitis, how accurate are presenting clinical features in predicting an infectious aetiology compared to PCR?
- *Background question*: What are the typical presenting clinical features of infectious encephalitis (including possible variations due to altered immune status)?
- *PICO2*: In adults with infectious encephalitis, does the presence of risk factors (i.e., immune status, travel history, infectious contacts, pets, water exposure, past medical history) compared to their absence result in a higher risk of specific infectious aetiologies (as confirmed by PCR)?

B. MICROBIOLOGICAL INVESTIGATIONS
- *PICO3*: In adults with suspected encephalitis, does LP before imaging result in deterioration or worse clinical outcome compared to no LP before imaging?
    - *Part of above question (3a)*: What are the contraindications for an immediate LP?
- *PICO4*: In adults with suspected encephalitis and a first non-diagnostic LP, does a second LP 48 hours later result in improved aetiological diagnosis?
- *PICO5*: In suspected sporadic infectious encephalitis, is individualized/specific CSF PCR better than standardized/multiplex CSF PCR panel for first-line detection of the causative pathogen?
    - *Part of above question (5a)*: Which CSF antibody testing is useful in the diagnosis of infectious encephalitis?
    - *Part of above question (5b)*: Are there additional specific testing panels for immunocompromised patients?
- *PICO6*: In patients with suspected infectious encephalitis and no aetiological diagnosis despite extensive investigation, who are deteriorationg, does brain biopsy change patient management compared to no brain biopsy?

C. ADDITIONAL INVESTIGATIONS
- *Background question*: What are the typical computer tomography (CT) and magnetic resonance imaging (MRI) abnormalities in infectious encephalitis?
- *PICO7*: In adults with suspected encephalitis, what is the diagnostic accuracy of specific features on CT and MRI to predict an infectious aetiology compared to PCR?
- *PICO8*: In patients with suspected infectious encephalitis and psychiatric features, does EEG change the patient management compared to no EEG?

D. TREATMENT
- *PICO9*: In patients with suspected encephalitis, does treatment with aciclovir improve patient outcome compared to no aciclovir administration?
    - *Part of above question (9a)*: If HSV is not confirmed when can aciclovir be stopped?

- *PICO10*: In patients with proven HSV encephalitis (positive CSF PCR or intrathecal antibody production), does administering aciclovir for more than two weeks reduce morbidity compared to two-week aciclovir therapy?
    - *Part of above question (10a)*: When should aciclovir resistance be suspected and what action should be taken?
- *PICO11*: In patients with proven HSV encephalitis, does the addition of corticosteroid therapy improve outcome compared to standard care alone?
    - *Part of above question (11a)*: If yes, when should steroids be introduced?
- *PICO12*: In patients with proven VZV encephalitis (positive CSF PCR or intrathecal antibody production), does treatment with high dose aciclovir (usually 20 mg/kg three times per day) compared to 10 mg/kg aciclovir three times per day reduce mortality and morbidity?
- *PICO13*: What is the impact on morbidity and mortality of prophylactic antiseizure treatment versus no prophylactic antiseizure treatment in patients with infectious encephalitis?

E. ONGOING CARE AND REHABILITATION

- *PICO14*: In patients with infectious encephalitis and deep/worsening coma score or status epilepticus, does admission to intensive care improve outcome and reduce mortality compared to no admission to intensive care?
- *PICO15*: In a patient with HSE with relapse, does autoantibody testing improve the chance of identifying the cause of relapse compared to not testing for autoantibodies?
- *PICO16*: In patients with infectious encephalitis, does follow-up care and rehabilitation reduce morbidity compared to no follow-up care and rehabilitation?

7. Search strategy

For each PICO question a systematic review of the literature using a predefined search strategy will be performed (see specific search strategies in **Appendix 2**). The literature search will be conducted querying the Medline database, via Pubmed interface. Studies listed from January 1, 2008 onwards will be included. Studies prior to this will not be searched for, as questions to be answered in these Guidelines were informed by the previous UK guidelines which systematically searched the literature up until 2008; however, relevant papers from the previous UK guidelines will be included [25]. The Cochrane Central Register of Controlled Trials (CENTRAL) will also be searched for relevant interventional trials. The bibliography of included papers will be reviewed for further relevant articles.

Cross-sectional and cohort; retrospective and prospective; observational studies, diagnostic accuracy studies and interventional trials; and reviews and meta-analyses will be evaluated (see **Appendix 1** for study types to be considered for each PICO). Both randomized and non-randomized studies will be considered for therapeutic questions, as very few randomized controlled trials exist and any useful information from other studies should thus be incorporated. Studies in a language other than English will be included only if an English abstract and reliable translation of the manuscript into English is available.

The titles/abstracts, and full text where necessary, of identified papers will be screened for eligibility by two independent reviewers (to reduce potential selection bias and to ensure consistency) according to the following inclusion/exclusion criteria:

- *Inclusion Criteria*:
    - Study design as defined by individual PICO (see Appendix 1; if case series are eligible, they must include ≥5 cases)
    - Studies available in English language
    - Studies relevant to question being addressed (each PICO will act as a checklist)
- *Exclusion Criteria*:
    - Single case reports
    - Case series including <5 cases
    - Editorials, letters, correspondence (which do not include primary data)
    - Studies including only paediatric populations
    - Studies in any language other than English for which reliable translation not available
    - Animal-only studies

Any disagreements in the inclusion/exclusion of articles will be discussed between the two reviewers to achieve consensus. If consensus cannot be achieved, the Chair or their delegate from the Task Force will make the final decision regarding study eligibility.

8. Methodology

8.1 Data collection and quality/risk of bias assessment

Selected data will be extracted from included studies into a standardized piloted template in Microsoft Excel, initially by one reviewer, then checked by another. In addition to relevant interventions and outcomes, the following will be recorded: 1) journal name and Vancouver-style reference, 2) study design (e.g., systematic review, cross-sectional study, etc.), 3) method of recruitment (e.g., prospective or retrospective), 4) study setting, and 5) characteristics of the patient population (e.g., age, gender, co-morbidities). Methodological quality will also be assessed for included studies, initially by one reviewer, then this will be checked by another. Standardized tools will be used to assess the quality of studies depending on the study type (i.e., Cochrane Collaboration's tool for assessing risk of bias in randomized trials; QUADAS-2 for diagnostic accuracy studies; Risk of Bias in Non-randomized Studies – of Interventions [ROBINS-I; Cochrane Bias Methods Group]; Joanna Briggs Institute & Newcastle-Ottawa Scale tools for non-interventional observational studies; and Assessing the Methodological Quality of Systematic Reviews [AMSTAR]). Any disagreements between reviewers on data collection and quality/risk of bias assessment will be resolved through consensus, and if there is ongoing disagreement, by the Task Force Chair or their delegate.

8.2 Evidence synthesis

Following extraction of the relevant literature for each PICO, the body of evidence available will be summarized in tables. Depending on the results of the literature search and review, a meta-analysis on available numerical data (e.g., clinical trial results) may be conducted,

subject to the comparability of studies, study design, and risk of bias/quality. Standard Cochrane methodology will be used for this. *Identified studies may be analyzed, if deemed appropriate, using a funnel plot (i.e., scatter plot of treatment effect versus study size) to address possible publication bias or overestimation of treatment effects in small studies of low quality.*

All factors that might decrease (e.g., study limitations/risk of bias, inconsistency of results, indirectness of evidence, imprecision, publication bias) or increase (e.g., large effect sizes, out-of-sample validation of results, and dose-response) quality of evidence will be considered. Where appropriate, certainty in the evidence will be evaluated using the Grading of Recommendations Assessment, Development, and Evaluation (GRADE) system and rated as high, moderate, low, or very low [35].

8.3 Developing recommendations

A GRADE approach will also be used to make recommendations based on the findings. Strength (strong, conditional, or weak) and direction (for or against) of recommendations will be determined based on balance between desirable and undesirable effects, quality of evidence, values, and preferences and costs. Summary of findings tables and evidence to decision tables will be prepared. Where a GRADE-based recommendation is not appropriate, a good practice statement will be made based on the available evidence.

Consensus on recommendations will be achieved within WGs through online consultation. A meeting of the wider Task Force will subsequently be held. Prior to this meeting, the output from each WG will be distributed to other Task Force members by the Task Force Chair and Senior coordinating advisor. At the meeting of the wider group, final recommendations can be endorsed by all members of the Task Force. A patient representative is part of the Task Force.

8.4 Manuscript preparation

The Senior coordinating advisor in close collaboration with the Chair, will prepare a first draft of the Guidelines manuscript. The draft will be circulated to Task Force members for comment and approval. The final manuscript will be submitted to the EAN *European Journal of Neurology* and ESCMID journal *Clinical Microbiology and Infection*. The final list of authors will include all Task Force members and appropriate others.

8.5 Timeline

Timelines for completion are within 12-18 months of this protocol being approved.

9 Consensus process

Disagreement when evaluating the literature and formulating the recommendations will be resolved by consensus within the respective WG specific to the PICO. If disagreement cannot be resolved within the WG, further discussion will involve all Task Force members and a final decision will be reached using the modified Delphi approach [36]. Task Force members with conflicts of interest in a particular recommendation can participate in the discussion but not vote for or against the recommendation.

10 References

1. Sköldenberg B, Alestig K, Burman L, et al. Acyclovir versus vidarabine in herpes simplex encephalitis. Lancet. 1984;324(8405):707-11.
2. Granerod J, Davies NWS, Ramanuj PP, et al. Increased rates of sequelae post-encephalitis in individuals attending primary care practices in the United Kingdom: a population-based retrospective cohort study. J Neurol. 2016;264(2):1-9.
3. Khandaker G, Jung J, Britton PN, et al. Long-term outcomes of infective encephalitis in children: a systematic review and meta-analysis. Dev Med Child Neurol. 2016 Nov;58(11):1108–15.
4. Whitley RJ. Herpes simplex encephalitis: adolescents and adults. Antiviral Res. 2006;71(2-3):141-8.
5. Mailles A, De Broucker T, Costanzo P, et al. Long-term outcome of patients presenting with acute infectious encephalitis of various causes in France. Clin Infect Dis. 2012;54(10):1455-64.
6. Vora NM, Holman RC, Mehal JM, et al. Burden of encephalitis-associated hospitalizations in the United States, 1998-2010. Neurology. 2014;82(5):443-51.
7. Whitley RJ, Cobbs CG, Alford CA Jr, et al. Diseases that mimic herpes simplex encephalitis. Diagnosis, presentation, and outcome. NIAD Collaborative Antiviral Study Group. JAMA 1989; 262: 234–39.
8. Granerod J, Ambrose HE, Davies NW, et al. Causes of encephalitis and differences in their clinical presentations in England: a multicentre, population-based prospective study. Lancet Infect Dis. 2010 Dec;10(12):835–44.
9. Mcgrath N, Anderson NE, Croxson MC, et al. Herpes simplex encephalitis treated with acyclovir: diagnosis and long term outcome. J Neurol Neurosurg Psychiatry. 1997;63:321-26.
10. Benson PC, Swadron SP. Empiric acyclovir is infrequently initiated in the emergency department to patients ultimately diagnosed with encephalitis. Ann Emerg Med. 2006;47(1):100-5.
11. Solomon T, Whitley R. In: Scheld M, Whitley RJ, Marra C, editors. Arthropod-borne viral encephalitides. Infections of the Central Nervous system. 2004.
12. Solomon T, Hart IJ, Beeching NJ, et al. Viral encephalitis : a clinician's guide. 2007;(October):288-305.
13. Boucher A, Herrmann JL, Morand P, et al. Epidemiology of infectious encephalitis causes in 2016. Med Mal Infect. 2017 May;47(3):221–35.
14. Arboviral Diseases, Neuroinvasive and Non-neuroinvasive | 2015 Case Definition. https://wwwn.cdc.gov/nndss/conditions/arboviral-diseases-neuroinvasive-and-non-neuroinvasive/case-definition/2015/. Accessed November 28, 2017.
15. Holzmann H. Diagnosis of tick-borne encephalitis. Vaccine. 2003;21(Suppl. 1):36-40
16. Cinque P, Cleator GM, Weber T, et al. The role of laboratory investigation in the diagnosis and management of patients with suspected herpes simplex encephalitis: a consensus report. The EU Concerted Action on Virus Meningitis and Encephalitis. J Neurol Neurosurg Psychiatry. 1996;61(4):339–45.
17. Kamei S. Evaluation of combination therapy using aciclovir and corticosteroid in adult patients with herpes simplex virus encephalitis. J Neurol Neurosurg Psychiatry. 2005;76(11):1544-49.

18. Martinez-Torres F, Menon S, Pritsch M, et al. Protocol for German trial of Acyclovir and corticosteroids in Herpes-simplex-virus-encephalitis (GACHE): a multicenter, multinational, randomized, double-blind, placebo-controlled German, Austrian and Dutch trial [ISRCTN45122933]. BMC Neurol. 2008;8:40.
19. Hart J, Tillman G, Kraut MA, et al. West Nile virus neuroinvasive disease: neurological manifestations and prospective longitudinal outcomes. BMC Infect Dis. 2014;14(1):248.
20. Chowers MY, Lang R, Nassar F, et al. Clinical characteristics of the West Nile fever outbreak, Israel, 2000. Emerg Infect Dis. 2001;7(4):675-78.
21. Kalil AC, Devetten MP, Singh S, et al. Use of interferon-alpha in patients with West Nile encephalitis: report of 2 cases. Clin Infect Dis. 2005;40(5):764-66.
22. Jackson AC, Warrell MJ, Rupprecht CE, et al. Management of rabies in humans. Clin Infect Dis. 2003;36(1):60-63.
23. Wengse C, Ericsson J, Hallberg S, et al. Rapid improvement of tick-borne encephalitis after treatment with corticosteroids. Lakartidningen. 2017;114:1-4.
24. Steiner I, Budka H, Chaudhuri A, et al. Viral meningoencephalitis: A review of diagnostic methods and guidelines for management. Eur J Neurol. 2010;17(8):999-1009.
25. Solomon T, Michael BD, Smith PE, et al. Management of suspected viral encephalitis in adults - Association of British Neurologists and British Infection Association National Guidelines. J Infect. 2012;64(4):347-73.
26. Unaid. Unaids Data 2017. 2017. doi:978-92-9173-945-5.
27. ECDC. Annual epidemiological report for 2015 – Syphilis [Internet]. 2017;(November). http://ecdc.europa.eu/en/publications/surveillance_reports/annual_epidemiological_report/Pages/epi_index.aspx#sthash.oeOLX1mU.dpuf.
28. ECDC. European Centre for Disease Prevention and Control. Annual Epidemiological Report for 2014- Rabies. 2016. doi:10.2903/j.efsa.2015.4329
29. Patel A, Small JE, Pradhan F, et al. Case Report: Zika virus meningoencephalitis and myelitis and associated magnetic resonance imaging findings. Am J Trop Med Hyg. 2017;97(2):340-43.
30. Ye M, Ren Y, Lv T. Encephalitis as a clinical manifestation of COVID-19. Brain Behav Immun. 2020;88:945-6.
31. Gossner CM, Marrama L, Carson M, et al. West nile virus surveillance in europe: Moving towards an integrated animal-human-vector approach. Eurosurveillance. 2017;22(18):1-10.
32. Radzišauskienė D, Žagminas K, Ašoklienė L, et al. Epidemiological patterns of tick-borne encephalitis in Lithuania and clinical features in adults in the light of the high incidence in recent years: a retrospective study. Eur J Neurol. 2017;12(10):3218-21.
33. Leone MA, Keindl M, Schapira AH, et al. Practical recommendations for the process of proposing, planning, and writing a neurological management guideline by EAN task forces. Eur J Neurol. 2015;22(12):1505-10.
34. ESCMID manual for clinical practice guidelines and other guidance documents. Second version, 24th June, 2020. https://www.escmid.org/fileadmin/src/media/PDFs/Guidelines/ESCMID_Guideline_Manual_Version_2.pdf. Accessed 16th November 2020.
35. Guyatt G, Oxman AD, Akl EA, et al. GRADE guidelines: 1. Introduction-GRADE evidence profiles and summary of findings tables. J Clin Epidemiol. 2011;64(4):383-39.
36. McMillan SS, King M, Tully MP. How to use the nominal group and Delphi techniques. Int J Clin Pharm. 2016;38:655–62.

Appendix 1. PICO questions

Question number	Question statement	PICO structure	Types of study to include
Step 1: When to "clinically" suspect infectious encephalitis			
Q1	In adults with suspected encephalitis, how accurate are presenting clinical features in predicting an infectious aetiology compared to PCR?	P: Adults with suspected encephalitis<br>I: Presenting clinical features<br>C: PCR testing<br>O: Diagnostic accuracy	Cross-sectional, cohort, diagnostic accuracy, interventional, meta-analyses, reviews, case series if ≥5 cases
Q2	*Background question: What are the typical presenting clinical features of infectious encephalitis (including possible variations due to altered immune status)?*<br>*PICO:* In adults with infectious encephalitis, does the presence of risk factors (i.e., immune status, travel history, infectious contacts, pets, water exposure, past medical history) compared to their absence result in a higher risk of specific infectious aetiologies (as confirmed by PCR)?	P: Adults with infectious encephalitis<br>I: Presence of risk factors<br>C: Absence of risk factors<br>O: Risk of specific infectious aetiologies (as confirmed by PCR)	Cross-sectional, cohort, diagnostic accuracy, interventional, meta-analyses, reviews, case series if ≥5 cases
Step 2: Microbiological investigations for infectious encephalitis			
Q3	In adults with suspected encephalitis, does lumbar puncture (LP) before imaging result in deterioration or worse clinical outcome compared to no LP before imaging?	P: Patients with suspected encephalitis<br>I: LP before imaging<br>C: No LP before imaging<br>O: Deterioration, outcome (as defined by Glasgow Outcome Score)	Cross-sectional, cohort, interventional, meta-analyses, reviews, case series if ≥5 cases
Q3a	What are the contraindications for an immediate LP?	Part of the above question	
Q4	In adults with suspected encephalitis and a first non-diagnostic LP, does a second LP 48 hours later result in improved aetiological diagnosis?	P: Patients with suspected encephalitis and first non-diagnostic LP<br>I: Second LP 48 hours after first LP<br>C: No second LP<br>O: Identification of aetiology by PCR	Cross-sectional, cohort, diagnostic accuracy, interventional, meta-analyses, reviews, case series if ≥5 cases
Q5	In suspected sporadic infectious encephalitis is individualized/specific CSF PCR better than standardized/multiplex CSF PCR panel for first-line detection of the causative pathogen?	P: Patients with suspected sporadic infectious encephalitis<br>I: Individualized/specific CSF PCR<br>C: Standardized/multiplex CSF PCR	Cross-sectional, cohort, diagnostic accuracy, interventional, meta-analyses, reviews, case series if ≥5 cases

		O: Detection of the causative pathogen with the first PCR test	
Q5a	Which CSF antibody testing is useful in the diagnosis of infectious encephalitis?	Part of the above question	
Q5b	Are there additional specific testing panels for immunocompromised patients?	Part of the above question	
Q6	In adults with suspected infectious encephalitis and no aetiological diagnosis despite extensive investigation, who are deteriorating, does brain biopsy change patient management compared to no brain biopsy?	P: Adults with suspected infectious encephalitis and no aetiological diagnosis despite extensive investigation, who are deteriorating<br>I: Brain biopsy<br>C. No brain biopsy<br>O: Change in patient management (e.g., stopping unnecessary drugs or initiating other treatment)	Cross-sectional, cohort, diagnostic accuracy, interventional, meta-analyses, reviews, case series if ≥5 cases
Step 3: Additional investigations for infectious encephalitis			
Q7	*Background question: What are the typical CT and MRI abnormalities in infectious encephalitis?*<br>*PICO*: In adults with suspected encephalitis, what is the diagnostic accuracy of specific features on CT and MRI to predict an infectious aetiology compared to PCR?	P: Patients with suspected encephalitis<br>I: Specific radiological features (e.g., bilateral asymmetrical involvement of the limbic system on MRI to predict HSE)<br>C: PCR testing<br>O: Diagnostic accuracy	Cross-sectional, cohort, diagnostic accuracy, interventional, meta-analyses, reviews, case series if ≥5 cases
Q8	In patients with suspected infectious encephalitis and psychiatric features, does EEG change the patient management compared to no EEG?	P: Patients with suspected infectious encephalitis and psychiatric features<br>I: EEG<br>C. No EEG<br>O: Change in patient management (e.g., stopping unnecessary drugs or initiating other treatment)	Cross-sectional, cohort, diagnostic accuracy, interventional, meta-analyses, reviews, case series if ≥5 cases
Step 4: How to treat infectious encephalitis			
Q9	In patients with suspected encephalitis, does treatment with aciclovir improve patient outcome compared to no aciclovir administration?	P: Patients with suspected encephalitis<br>I: Aciclovir<br>C: No aciclovir<br>O: Death, morbidity (as defined by Glasgow Outcome Score)	Cross-sectional, cohort, interventional, meta-analyses, reviews, case series if ≥5 cases
Q9a	If HSV is not confirmed when can aciclovir be stopped?	Part of the above question	
Q10	In patients with proven HSV encephalitis (positive CSF PCR or intrathecal antibody production), does administering aciclovir	P: Patients with proven HSV encephalitis<br>I: Aciclovir for longer than two weeks<br>C: Two-week aciclovir therapy	Cross-sectional, cohort, interventional, meta-

	for more than two weeks reduce morbidity compared to two-week aciclovir therapy?	O: Morbidity as defined by Glasgow Outcome Score	analyses, reviews, case series if ≥5 cases
Q10a	When should aciclovir resistance be suspected and what action should be taken?	Part of the above question	
Q11	In patients with proven HSV encephalitis, does the addition of corticosteroid therapy improve outcome compared to standard care alone?	P: Patients with proven HSV encephalitis<br>I: Addition of corticosteroid therapy<br>C: Standard care without corticosteroid therapy<br>O: Glasgow Outcome Score	Cross-sectional, cohort, interventional, meta-analyses, reviews, case series if ≥5 cases
Q11a	If yes, when should steroids be introduced?	Part of the above question	
Q12	In patients with proven VZV encephalitis (positive CSF PCR or intrathecal antibody production), does treatment with high dose aciclovir (usually 20 mg/kg three times per day) compared to 10 mg/kg aciclovir three times per day reduce mortality and morbidity?	P: Patients with VZV encephalitis<br>I: High dose aciclovir (i.e., 20 mg/kg three times per day)<br>C: Aciclovir 10 mg/kg three times per day<br>O: Death, morbidity (as defined by GOS)	Cross-sectional, cohort, interventional, meta-analyses, reviews, case series if ≥5 cases
Q13	What is the impact on morbidity and mortality of prophylactic antiseizure treatment versus no prophylactic antiseizure treatment in cases of infectious encephalitis?	P: Patients with infectious encephalitis<br>I: Prophylactic antiseizure treatment<br>C: No prophylactic antiseizure treatment<br>O: Death, morbidity (as defined by GOS)	Cross-sectional, cohort, interventional, meta-analyses, reviews, case series if ≥5 cases
Step 5: Ongoing care and rehabilitation			
Q14	In patients with infectious encephalitis and deep/worsening coma score or status epilepticus, does admission to intensive care improve outcome and reduce mortality compared to no admission to intensive care?	P: Patients with infectious encephalitis and deep/worsening coma score or status epilepticus<br>I: Admission to intensive care unit<br>C: No admission to intensive care unit<br>O: Death, morbidity (as defined by GOS)	Cross-sectional, cohort, interventional, meta-analyses, reviews, case series if ≥5 cases
Q15	In a patient with herpes simplex encephalitis (HSE) with relapse, does autoantibody testing improve the chance of identifying the cause of relapse compared to not testing for autoantibodies?	P: Adult with HSE and relapse<br>I: Testing for autoantibodies<br>C: No testing for autoantibodies<br>O: Identification of cause of relapse	Cross-sectional, cohort, diagnostic accuracy, interventional, meta-analyses, reviews, case series if ≥5 cases
Q16	In patients with infectious encephalitis, does follow-up care and rehabilitation reduce morbidity compared to no follow-up care and rehabilitation?	P: Patients with infectious encephalitis<br>I: Follow-up care and rehabilitation<br>C: No follow-up care or rehabilitation<br>O: Morbidity as defined by GOS	Cross-sectional, cohort, interventional, meta-analyses, reviews, case series if ≥5 cases

Appendix 2. Search strategies

<u>Step 1: When to suspect infectious encephalitis</u>

Combined search for PICO1, background question, and PICO2:

(("infectious encephalitis"[MeSH Terms] OR "encephalit*"[Title/Abstract]) AND ("symptom"[Title/Abstract] OR "sign"[Title/Abstract] OR "syndrom*"[Title/Abstract] OR "feature"[Title/Abstract] OR "presentation"[Title/Abstract] OR ("causality"[MeSH Terms] OR "aetiology"[Title/Abstract] OR "cause"[Title/Abstract] OR "causative agent"[Title/Abstract] OR "pathogen"[Title/Abstract] OR "organism"[Title/Abstract]))) AND (2008:2021[pdat]) = ***9,398 results***

For searches with >1,000 results, a hierarchical approach will be used:

a) Limit the search to systematic reviews. If a systematic review is found that answers the question comprehensively, the primary articles search will start from the date of that review's search.
b) If no relevant systematic review is found, all primary studies will be screened. For searches with >1,000 results, this will be conducted by one reviewer initially, with 20% quality control checked by another reviewer. After the first 1,000 results, we will stop and recalibrate if >10% of decisions are discrepant.

Meeting of core group 14 Jan 2022
No systematic reviews found
Also noticed lack of PCR search term which would make results much less specific for these PICOs.

- ➔ Revised search strategy including PCR:

(("polymerase chain reaction"[MeSH Terms]) OR (PCR[Title/Abstract])) AND ((("infectious encephalitis"[MeSH Terms] OR "encephalit*"[Title/Abstract]) AND ("symptom"[Title/Abstract] OR "sign"[Title/Abstract] OR "syndrom*"[Title/Abstract] OR "feature"[Title/Abstract] OR "presentation"[Title/Abstract] OR ("causality"[MeSH Terms] OR "aetiology"[Title/Abstract] OR "cause"[Title/Abstract] OR "causative agent"[Title/Abstract] OR "pathogen"[Title/Abstract] OR "organism"[Title/Abstract]))) AND (2008:2021[pdat]))
= 1,170 results on 14/1/22

<u>Step 2: Microbiological investigations for infectious encephalitis</u>

For PICO3 (including 3a) and PICO4:

(("infectious encephalitis"[MeSH Terms] OR "encephalit*"[Title/Abstract]) AND ("spinal puncture"[MeSH Terms] OR "spinal puncture"[MeSH Terms] OR "spinal puncture"[MeSH Terms] OR "lumbar puncture"[Title/Abstract] OR "spinal tap"[Title/Abstract] OR "spinal

puncture"[Title/Abstract] OR "centesis"[Title/Abstract])) AND (2008:2021[pdat]) = ***278 results***

For PICO5 (including 5b):

(("infectious encephalitis"[MeSH Terms] OR "encephalitis"[Title/Abstract]) AND ("cerebrospinal fluid"[MeSH Terms] OR "CSF"[Title/Abstract] OR "spinal fluid"[Title/Abstract]) AND ("pcr"[Title/Abstract] OR "polymerase chain reaction"[Title/Abstract] OR "multiplex polymerase chain reaction"[MeSH Terms] OR "multiplex pcr"[MeSH Terms])) AND (2008:2021[pdat]) = ***674 results***

For 5a and 5b:

(("encephalit*"[Title/Abstract] OR "infectious encephalitis"[MeSH Terms]) AND (("cerebrospinal fluid"[MeSH Terms] OR "CSF"[Title/Abstract] OR "spinal fluid"[Title/Abstract]) AND ("oligoclonal bands"[MeSH Terms] OR "oligoclonal band"[Title/Abstract] OR "antibody test*"[Title/Abstract] OR "intrathecal antibody"[Title/Abstract]))) AND (2008:2021[pdat]) = ***78 results***

For PICO6:

(("infectious encephalitis"[MeSH Terms] OR "encephalit*"[Title/Abstract]) AND ("brain biopsy"[Title/Abstract] OR "biopsy"[MeSH Terms])) AND (2008:2021[pdat]) = ***413 results***

<u>Step 3: Additional investigations for infectious encephalitis</u>

Combined search for background question, PICO7, and PICO8:

(("infectious encephalitis"[MeSH Terms] OR "encephalit*"[Title/Abstract]) AND ("electroencephalography"[MeSH Terms] OR "eeg"[Title/Abstract] OR "electroencephalogr*"[Title/Abstract] OR "electroencephalography"[MeSH Terms] OR "electroencephalography"[MeSH Terms] OR ("magnetic resonance imaging"[MeSH Terms] OR "neuroimaging"[MeSH Terms] OR "four dimensional computed tomography"[MeSH Terms] OR "CT"[Title/Abstract] OR "MRI"[Title/Abstract] OR "imaging"[Title/Abstract] OR "scan"[Title/Abstract] OR "radiolog*"[Title/Abstract]))) AND (2008:2021[pdat]) = ***5,172 results***

As >1,000 results, use a hierarchical approach as described above.

Meeting of core group 14 Jan 2022
No systematic reviews found
Noticed lack of PCR search term which would make results much less specific for PICO 7, which should then have a separate search strategy.
Also noticed lack of psychiatric symptom search terms which would make results much less specific for PICO 8.

- ➔ Revised search strategy for **<u>PICO 7</u>** including PCR:

(("polymerase chain reaction"[MeSH Terms]) OR (PCR[Title/Abstract])) AND ((("infectious encephalitis"[MeSH Terms] OR "encephalit*"[Title/Abstract]) AND ("magnetic resonance imaging"[MeSH Terms] OR "neuroimaging"[MeSH Terms] OR "CT"[Title/Abstract] OR "MRI"[Title/Abstract] OR "imaging"[Title/Abstract] OR "scan"[Title/Abstract] OR "radiolog*"[Title/Abstract])) AND (2008:2021[pdat]))
= 469 results on 14/1/22

➔ Revised search strategy for **PICO 8** including psychiatric features:
((((((delusion*[Title/Abstract]) OR (depress*[Title/Abstract])) OR (agitat*[Title/Abstract])) OR (hallucinat*[Title/Abstract])) OR (psych*[Title/Abstract])) OR (neuropsychiatr*[Title/Abstract])) AND (("infectious encephalitis"[MeSH Terms] OR "encephalit*"[Title/Abstract]) AND ("electroencephalography"[MeSH Terms] OR "eeg"[Title/Abstract] OR "electroencephalogr*"[Title/Abstract] OR "electroencephalography"[MeSH Terms] OR "electroencephalography"[MeSH Terms]) AND (2008:2021[pdat]))
= 343 results on 14/1/22

Step 4: How to treat infectious encephalitis

For PICO9 (including 9a) and PICO10 (including 10a):

(("infectious encephalitis"[MeSH Terms] OR "encephalit*"[Title/Abstract]) AND ("acyclovir"[Title/Abstract] OR "acyclovir"[MeSH Terms])) AND (2008:2021[pdat]) = ***702 results***

For PICO11 (including 11a):

(((("infectious encephalitis"[MeSH Terms] OR "encephalit*"[Title/Abstract]) AND ("hsv"[Title/Abstract] OR "herpes"[Title/Abstract] OR "herpes simplex"[MeSH Terms] OR "herpesvirus 1, human"[MeSH Terms])) OR "encephalitis, herpes simplex"[MeSH Terms]) AND ("hydrocortisone"[MeSH Terms] OR "prednisolone"[MeSH Terms] OR "methylprednisolone"[MeSH Terms] OR "dexamethasone"[MeSH Terms] OR "glucocorticoids"[MeSH Terms] OR "steroids/drug therapy"[MeSH Terms] OR "corticosteriod"[Title/Abstract] OR "hydrocortisone"[Title/Abstract] OR "prednisolone"[Title/Abstract] OR "methylprednisolone"[Title/Abstract] OR "dexamethasone"[Title/Abstract] OR "glucocorticoid"[Title/Abstract] OR "steroid"[Title/Abstract])) AND (2008:2021[pdat]) = ***98 results***

For PICO12:

((((infectious encephalitis[MeSH Terms]) OR (encephalit*[Title/Abstract])) OR (cerebellitis[Title/Abstract])) AND ((((varicella[Title/Abstract]) OR (zoster[Title/Abstract])) OR (vzv[Title/Abstract])) OR (encephalitis, varicella zoster[MeSH Terms]))) AND ((acyclovir[MeSH Terms]) OR (acyclovir[Title/Abstract])) = ***132 results***

For PICO13:

(("infectious encephalitis"[MeSH Terms] OR "encephalit*"[Title/Abstract]) AND ("valproic acid"[Title/Abstract] OR "clonazepam"[Title/Abstract] OR "zonisamide"[Title/Abstract] OR "topiramate"[Title/Abstract] OR "lamotrigine"[Title/Abstract] OR "vigabatrin"[Title/Abstract] OR "lacosamide"[Title/Abstract] OR "pregabalin"[Title/Abstract] OR "gabapentin"[Title/Abstract] OR "oxcarbazepine"[Title/Abstract] OR "carbamazepine"[Title/Abstract] OR "phenobarbital"[Title/Abstract] OR "phenytoin"[Title/Abstract] OR "anticonvulsants"[Title/Abstract] OR "antiseizure therapy"[Title/Abstract] OR "levetiracetam"[Title/Abstract] OR "rufinamide"[Title/Abstract] OR "topiramate"[MeSH Terms] OR "anticonvulsants"[MeSH Terms] OR "levetiracetam"[MeSH Terms] OR "phenytoin"[MeSH Terms] OR "phenobarbital"[MeSH Terms] OR "carbamazepine"[MeSH Terms] OR "oxcarbazepine"[MeSH Terms] OR "gabapentin"[MeSH Terms] OR "pregabalin"[MeSH Terms] OR "lacosamide"[MeSH Terms] OR "vigabatrin"[MeSH Terms] OR "lamotrigine"[MeSH Terms] OR "zonisamide"[MeSH Terms] OR "clonazepam"[MeSH Terms] OR "valproic acid"[MeSH Terms])) AND (2008:2021[pdat]) = ***382 results***

<u>Step 5: Ongoing care and rehabilitation</u>

For PICO14:

(("encephalit*"[Title/Abstract] OR "infectious encephalitis"[MeSH Terms]) AND ("critical care"[MeSH Terms] OR "critical care"[MeSH Terms] OR "critical care"[MeSH Terms] OR "intensive therapy unit"[Title/Abstract] OR "icu"[Title/Abstract] OR "itu"[Title/Abstract])) AND (2008:2021[pdat]) = ***209 results***

For PICO15:

(((("infectious encephalitis"[MeSH Terms] OR "encephalit*"[Title/Abstract]) AND ("hsv"[Title/Abstract] OR "herpes"[Title/Abstract] OR "herpesvirus 1, human"[MeSH Terms] OR "herpes simplex"[MeSH Terms])) OR "encephalitis, herpes simplex"[MeSH Terms]) AND ("recurrence"[MeSH Terms] OR "relaps*"[Title/Abstract] OR "recurr*"[Title/Abstract] OR "recurrence"[MeSH Terms])) AND (2008:2021[pdat]) = ***223 results***

For PICO16:

(("infectious encephalitis"[MeSH Terms] OR "encephalit*"[Title/Abstract]) AND ("rehabilitation"[MeSH Terms] OR "rehab*"[Title/Abstract] OR "follow-up"[Title/Abstract] OR "long-term"[Title/Abstract] OR "post-acute"[Title/Abstract] OR "postacute"[Title/Abstract] OR "management"[Title/Abstract] OR "length of stay"[Title/Abstract] OR "recovery of function"[Title/Abstract] OR "functional outcome"[Title/Abstract] OR "functional independence measure"[Title/Abstract] OR "functional recovery"[Title/Abstract] OR "discharge destination"[Title/Abstract] OR "discharge status"[Title/Abstract] OR "neuropsychology"[MeSH Terms])) AND (2008:2021[pdat]) ***= 3,190 results***

As >1,000 results, use a hierarchical approach as described above.

1. In adults with suspected encephalitis, how accurate are presenting clinical features in predicting an infectious aetiology compared to PCR?

Evidence summary

Of 1,170 studies identified by the search, six were included initially (1–6). See **Figure S1.1** for flow diagram and **Table S1.1** for characteristics of included studies. The studies included a total of 564 participants from 1997 to 2017. The Working Group (WG) focused on clinical features which are potentially discriminatory for aetiologies of suspected encephalitis, rather than generic features. These were altered behaviour, seizures, coma, fever, and focal neurological signs. Sensitivities, specificities, and predictive values of these features for predicting HSV and VZV from included studies were generally suboptimal (**Tables S1.2** and **S1.3**). No included study was deemed at high risk of bias or had applicability concerns through the quality assessments (**Table S1.4**). Heterogeneity in definitions used, comparator causes, and location resulted in limited the pooled data to only two studies (**Figures S1.2-S1.4**) (4,5). The TF concluded that there was insufficient direct evidence to inform a recommendation, and that a good practice statement should caution against clinical features to distinguish different infectious causes of encephalitis. They also proposed that **Table 2** (in the main manuscript) could prompt inclusion of some pathogens in the differential diagnosis for patients with specific clinical features.

Search string

(("polymerase chain reaction"[MeSH Terms]) OR (PCR[Title/Abstract])) AND ((("infectious encephalitis"[MeSH Terms] OR "encephalit*"[Title/Abstract]) AND ("symptom"[Title/Abstract] OR "sign"[Title/Abstract] OR "syndrom*"[Title/Abstract] OR "feature"[Title/Abstract] OR "presentation"[Title/Abstract] OR ("causality"[MeSH Terms] OR "aetiology"[Title/Abstract] OR "cause"[Title/Abstract] OR "causative agent"[Title/Abstract] OR "pathogen"[Title/Abstract] OR "organism"[Title/Abstract]))) AND (2008:2021[pdat]))

Flowchart

Figure S1.1. Flow chart for study inclusion for PICO 1

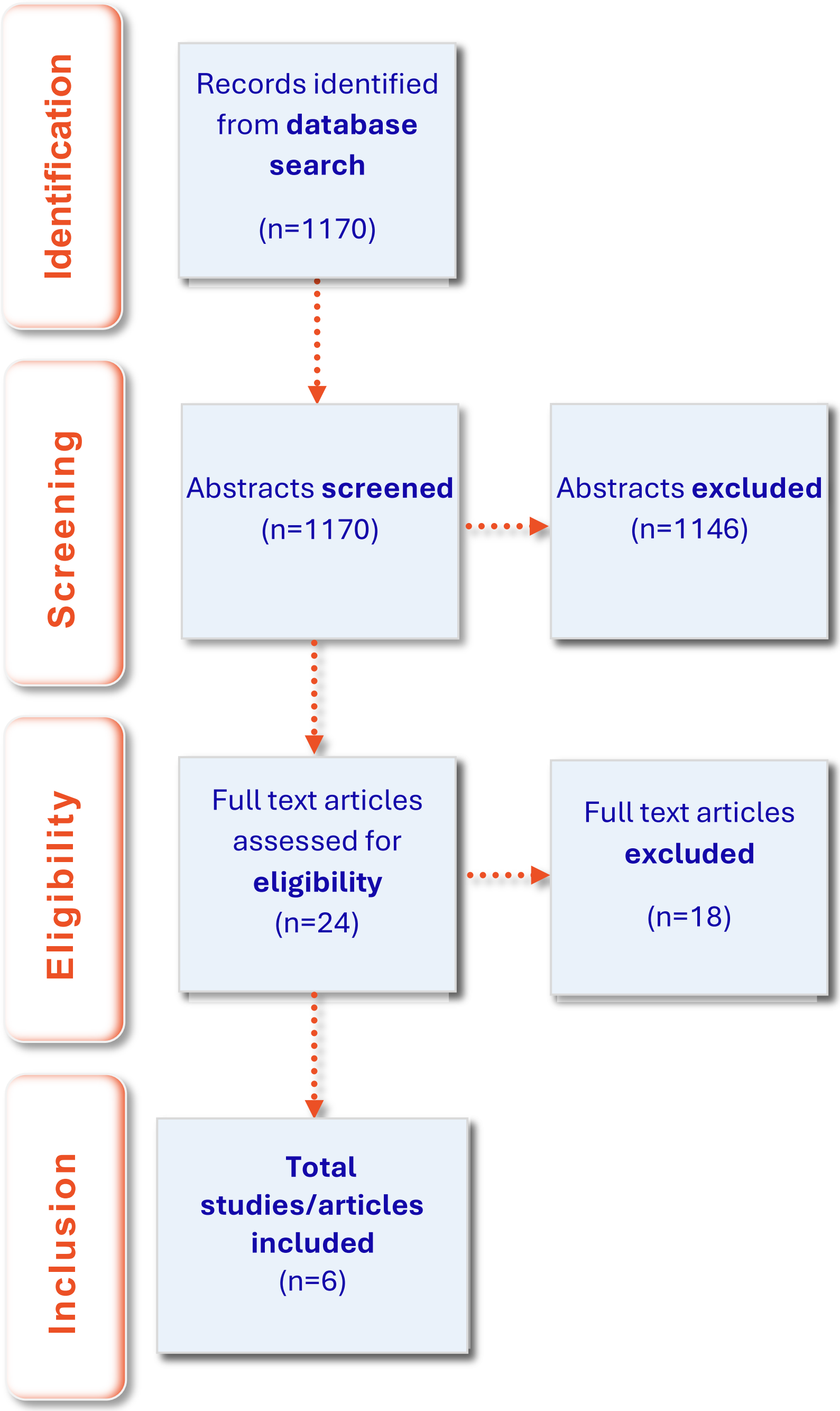

Characteristics of included studies

Table S1.1. Characteristics of included studies for PICO 1

First author	Country	Study period	Study design	Recruitment method	Number of participants
Basaran	Turkey	2011-2017	Cohort	Retrospective	Total: 67<br>HSV-1: 16; Non-HSV-1 viral encephalitis: 51
Jordan	Germany	2000-2014	Cohort	Retrospective	Total: 49<br>HSV: 15; VZV: 5; EBV: 2; Unknown: 27
Pleumpanupat	Thailand	1997-2006	Case-control	Retrospective	Total: 29<br>HSV: 7; Other viral encephalitis: 22 (1 CMV, 1 JC virus, I enterovirus, 1 JEV)
Ciftci	Turkey	2010-2013	Cohort	Retrospective	Total: 68<br>HSV: 7; PCR-negative viral encephalitis: 48; Alternative diagnosis (other confirmed pathogens, meningitis, or degenerative diseases): 13
Misra	India	2003-2006	Cohort	Prospective	Total: 148<br>JEV: 59; Dengue: 42; HSV: 8; Nonspecific: 35; Others: 4
Granerod	United Kingdom	2005-2008	Cohort	Prospective	Total: 268<br>ADEM: 23; Antibody: 16; Bacterial: 13; HSV: 38; MTB: 10; Non-encephalitis: 59; Other: 21; Unknown: 76; VZV: 12

ADEM: Acute disseminated encephalomyelitis; CMV: Cytomegalovirus; EBV: Epstein Barr virus; HSV: Herpes simplex virus; JEV: Japanese encephalitis virus; MTB: *Mycobacterium tuberculosis*; PCR: Polymerase chain reaction; VZV: Varicella zoster virus

Table S1.2. Presence of seizures as a presenting feature of HSV amongst included studies

Study	Comparator	TP	TN	FP	FN	Sensitivity	Specificity	NPV	PPV
**Basaran**	Other viral encephalitis	7	37	14	9	43.75%	72.55%	80.43%	33.33%
**Ciftci**	PCR-negative viral encephalitis and alternative diagnosis	0	58	3	7	0%	95.08%	89.23%	0%
**Granerod**	Other viral, bacterial, and autoimmune encephalitides and unknown	24	76	71	14	63.16%	51.70%	84.44%	25.26%
**Jordan**	Other viral encephalitis (known and unknown)	10	23	11	5	66.67%	67.65%	82.14%	47.62%
**Misra**	Other viral and also nonspecific encephalitis	6	83	57	2	75%	59.29%	97.65%	9.52%
**Pleumpanupat**	Other viral encephalitis (known and unknown)	3	16	6	4	42.86%	72.73%	80%	33.33%

FN: False negative; FP: False positive; NPV: Negative predictive value; PCR: Polymerase chain reaction; PPV: Positive predictive value; TN: True negative; TP: True positive

Table S1.3. Presence of seizures and fever as a presenting feature of VZV amongst included studies

Study	Comparator	TP	TN	FP	FN	Sensitivity	Specificity	NPV	PPV
Seizures									
**Granerod**	Other viral, bacterial, and autoimmune encephalitides and unknown	1	81	94	9	10%	46.29%	90%	1.05%
**Jordan**	Other viral encephalitis (known and unknown)	1	24	20	4	20%	54.54%	85.71%	4.76%
Fever									
**Granerod**		5	44	131	5	50%	25.14%	89.80%	3.68%
**Jordan**		0	22	22	5	0%	50%	81.48%	0%

FN: False negative; FP: False positive; NPV: Negative predictive value; PPV: Positive predictive value; TN: True negative; TP: True positive

Forest plots

Figure S1.2. Pooled sensitivity and specificity of seizures for the identification of HSV encephalitis

Sensitivity

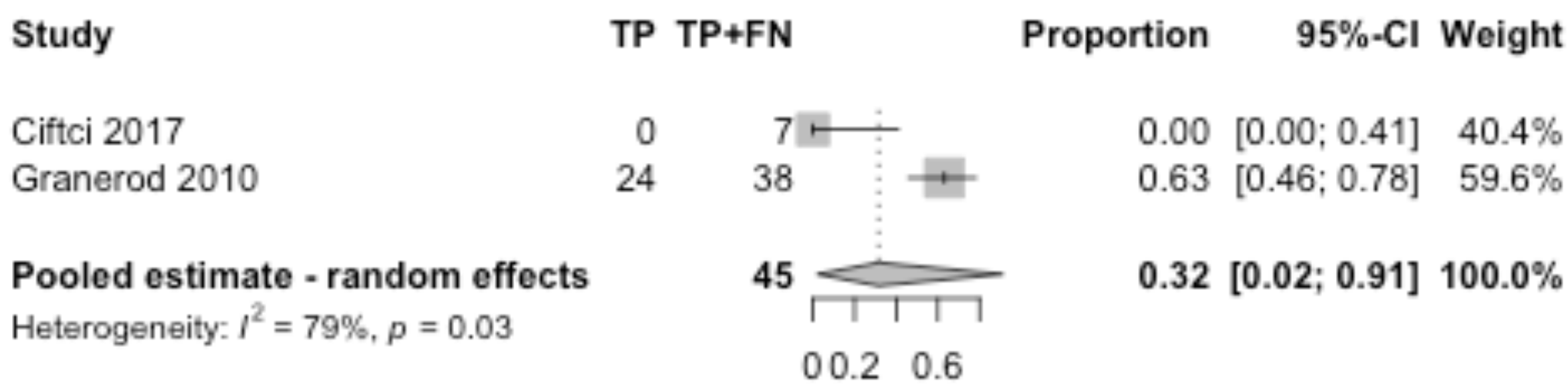

Specificity

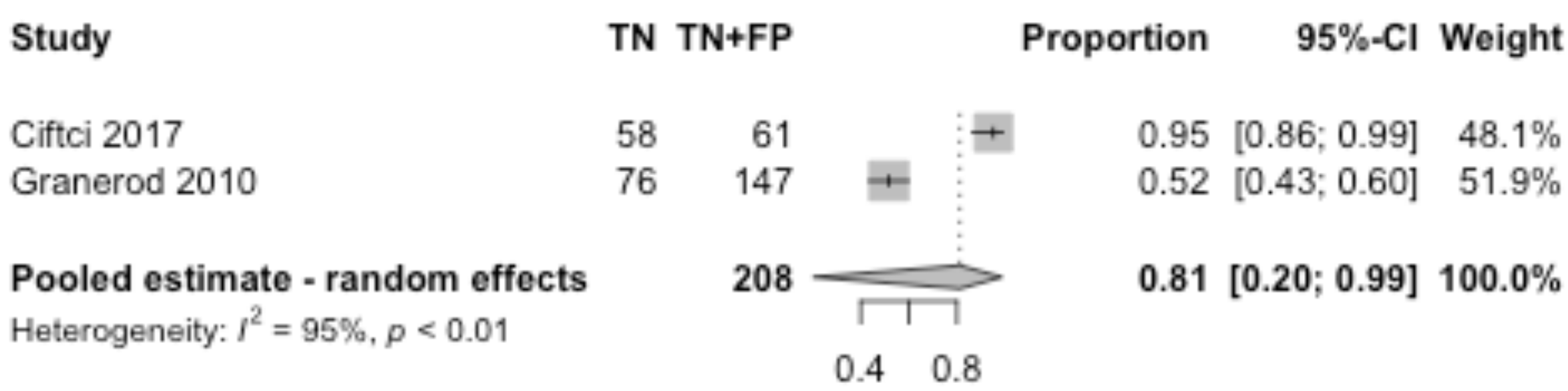

Figure S1.3. Pooled sensitivity and specificity of fever for the identification of HSV encephalitis

Sensitivity

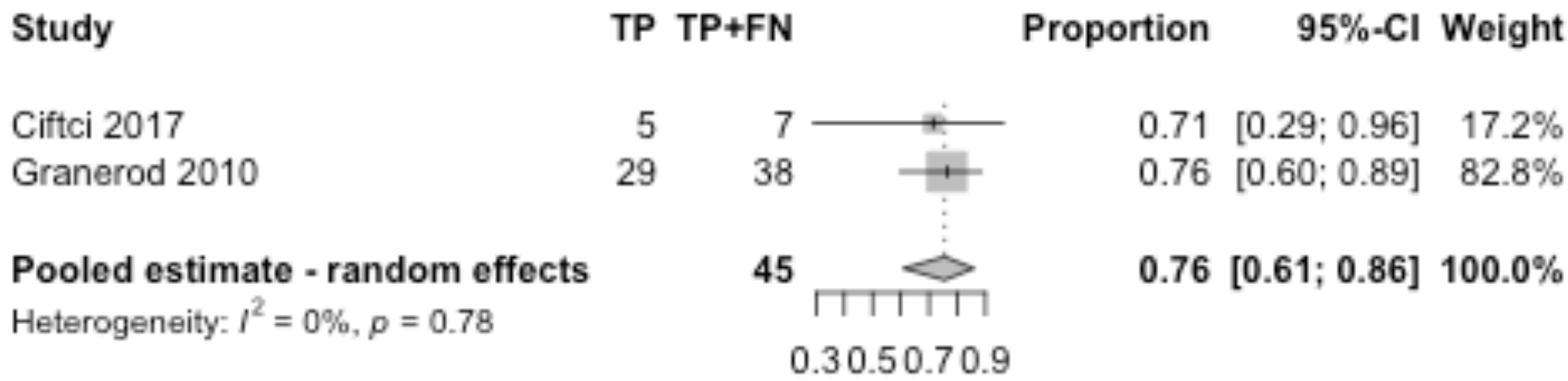

Specificity

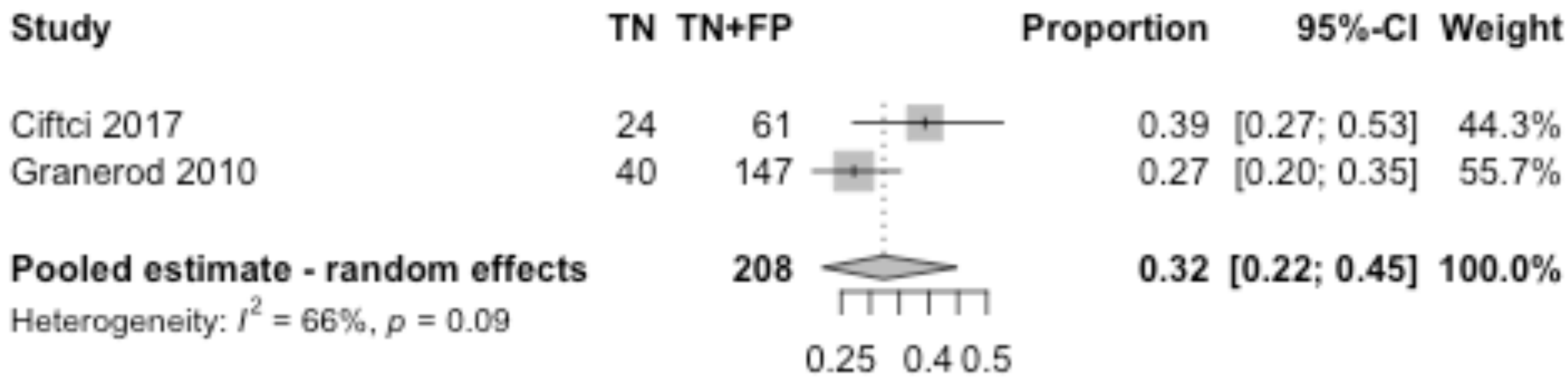

Figure S1.4. Sensitivity and specificity of altered behaviour for the identification of HSV encephalitis

Sensitivity

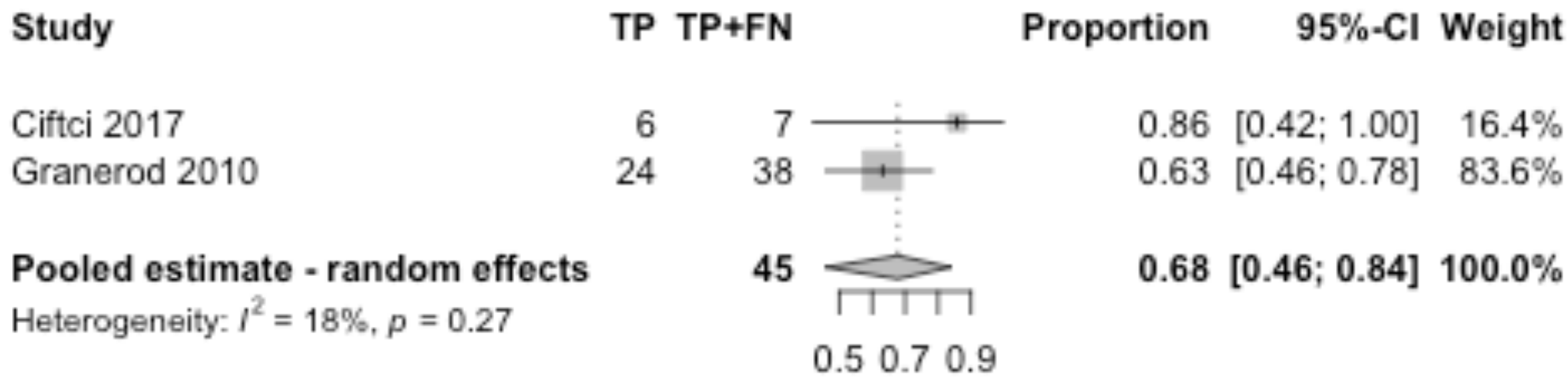

Specificity

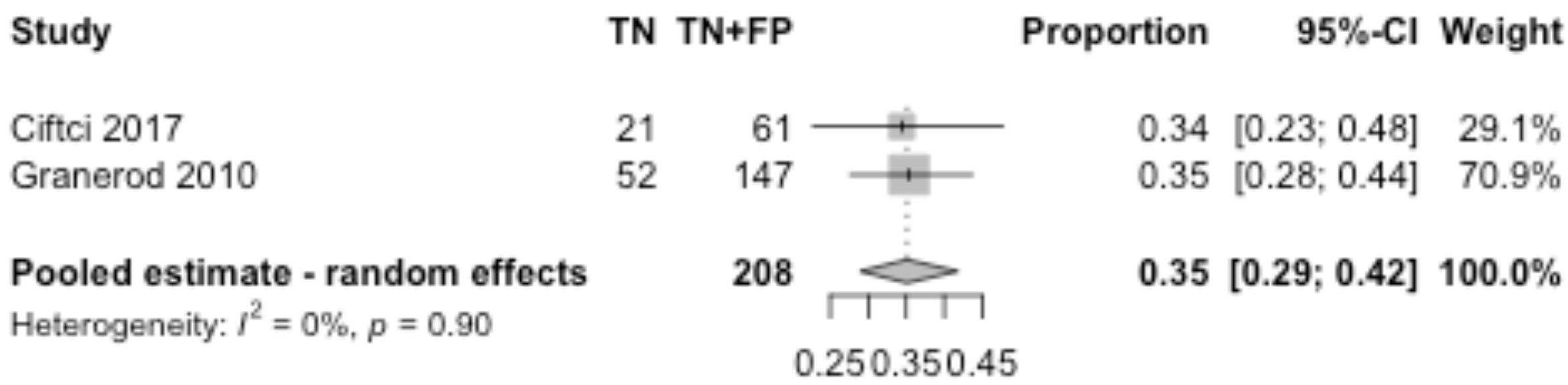

Quality assessment

Table S1.4. Quality assessment of included diagnostic accuracy studies using QUADAS-2

Study	Risk of bias				Applicability concerns		
	Patient selection	Index test	Reference standard	Flow & timing	Patient selection	Index test	Reference standard
Granerod	LR	UR	LR	LR	LR	LR	LR
Misra	UR	LR	LR	UR	LR	LR	LR
Jordan	LR	UR	UR	LR	LR	LR	LR
Pleumpanupat	UR	UR	UR	LR	LR	LR	LR
Basaran	LR	UR	UR	UR	LR	LR	LR
Ciftci	LR	UR	UR	LR	LR	LR	LR

LR = Low risk; UR = Unclear risk

2. In adults with infectious encephalitis, does the presence of risk factors (i.e., immune status, travel history, infectious contacts, pets, water exposure, past medical history) compared to their absence result in a higher risk of specific infectious aetiologies (as confirmed by PCR)?

Evidence summary

Four papers were included of 1,170 studies identified by the search (1,4,5,7). See **Figure S2.1** for flow diagram and **Table S2.1** for characteristics of included studies. The studies included a total of 678 participants with data from 2000 to 2017. Included studies reported on age, sex, and preceding upper respiratory tract infection (URTI) as potential risk factors for HSV and/or VZV encephalitis. Sensitivities, specificities, and predictive values of age, sex, and preceding URTI for predicting HSV and VZV encephalitis were generally suboptimal (**Tables S2.2** and **S2.3**). No included study was deemed at high risk of bias or had applicability concerns through the quality assessments (**Table S2.4**). Data could not be pooled through meta-analysis due to missing data, indirectness, and not primarily performing a risk factor analysis for causative pathogens. Two that aligned closely with the PICO, suggested older age may predict HSV as a causative pathogen, but the certainty in this was too low and overall the evidence was too indirect to support a recommendation (4,7).

Search string

(("polymerase chain reaction"[MeSH Terms]) OR (PCR[Title/Abstract])) AND ((("infectious encephalitis"[MeSH Terms] OR "encephalit*"[Title/Abstract]) AND ("symptom"[Title/Abstract] OR "sign"[Title/Abstract] OR "syndrom*"[Title/Abstract] OR "feature"[Title/Abstract] OR "presentation"[Title/Abstract] OR ("causality"[MeSH Terms] OR "aetiology"[Title/Abstract] OR "cause"[Title/Abstract] OR "causative agent"[Title/Abstract] OR "pathogen"[Title/Abstract] OR "organism"[Title/Abstract]))) AND (2008:2021[pdat]))

Flowchart

Figure S2.1. Flow chart for study inclusion for PICO 2

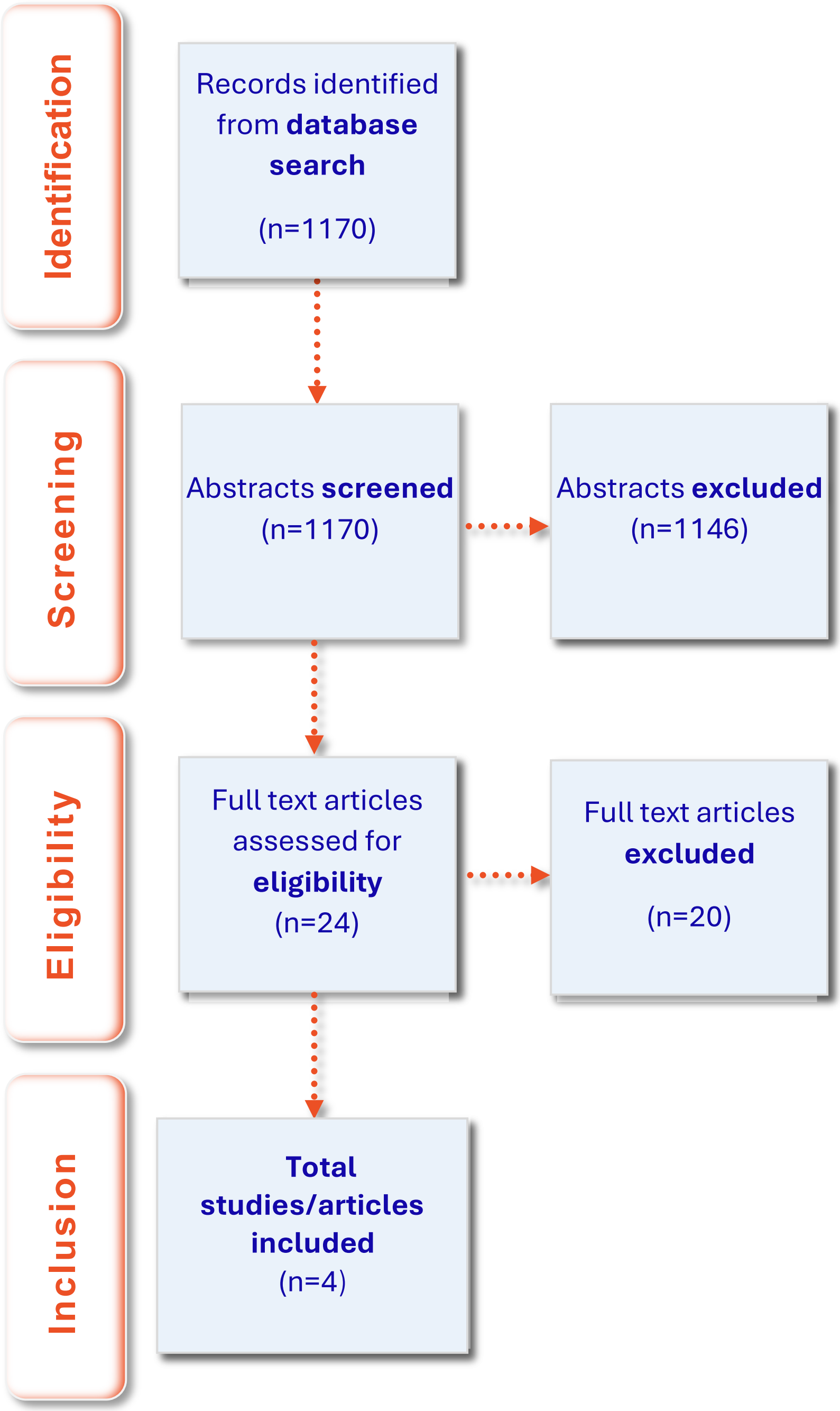

Characteristics of included studies

Table S2.1. Characteristics of included studies for PICO 2

First author	Country	Study period	Study design	Recruitment method	Number of participants
Basaran	Turkey	2011-2017	Cohort	Retrospective	Total: 67<br>HSV-1: 16; Non-HSV-1 viral encephalitis: 51
Ciftci	Turkey	2010-2013	Cohort	Retrospective	Total: 68<br>HSV: 7; PCR-negative viral encephalitis:48; Alternative diagnosis (other confirmed pathogens, meningitis, or degenerative diseases): 13
Granerod	United Kingdom	2005-2008	Cohort	Prospective	Total: 268<br>ADEM: 23; Antibody: 16; Bacterial: 13; HSV: 38; MTB: 10; Non-encephalitis: 59; Other: 21; Unknown: 76; VZV: 12
Hansen	2000-2017	US	Retrospective cohort	All encephalitis	Total: 340<br>Bacterial: 24; Viral: 52; Arbovirus: 34; Fungal: 11; Parasitic: 6; Autoimmune: 20; Unknown: 193

ADEM: Acute disseminated encephalomyelitis; HSV: Herpes simplex virus; MTB: *Mycobacterium tuberculosis*; PCR: Polymerase chain reaction; VZV: Varicella zoster virus

Table S2.2. Sensitivity, specificity, NPV, and PPV of age, sex, and preceding URTI as predictors of HSV encephalitis

Study	Comparator	TP	TN	FP	FN	Sensitivity	Specificity	NPV	PPV
**Age >65 years**									
**Granerod**	Other viral, bacterial, and autoimmune encephalitides and unknown	8	140	25	30	21.05%	84.85%	82.35%	24.24%
**Hansen**	Other bacterial, fungal, and viral encephalitis, NMDAR, and unknown	9	262	53	16	36%	83.17%	94.24%	14.52%
**Sex female**									
**Basaran**	Other viral encephalitis	10	34	17	6	62.5%	66.67%	85%	37.04%
**Ciftci**	PCR-neg viral encephalitis and alternative diagnosis	5	30	31	2	71.43%	49.18%	93.75%	13.89%
**Preceding URTI**									
**Basaran**		1	33	18	15	6.25%	64.71%	68.75%	5.26%
**Ciftci**		2	49	12	5	28.57%	80.33%	90.74%	14.28%

FN: False negative; FP: False positive; NMDAR: N-methyl-D-aspartate receptor; NPV: Negative predictive value; PCR: Polymerase chain reaction; PPV: Positive predictive value; TN: True negative; TP: True positive; URTI: Upper respiratory tract infection

Table S2.3. Sensitivity, specificity, NPV, and PPV of age as a predictor of VZV encephalitis

Study	Comparator	TP	TN	FP	FN	Sensitivity	Specificity	NPV	PPV
**Age >65 years**									
**Granerod**	Other viral, bacterial, and autoimmune encephalitides and unknown	2	162	31	8	20%	83.94%	95.29%	6.06%
**Hansen**	Other bacterial, fungal, and viral encephalitis, NMDAR, and unknown	2	269	60	9	18.18%	81.76%	96.76%	3.23%

FN: False negative; FP: False positive; NMDAR: N-methyl-D-aspartate receptor; NPV: Negative predictive value; PCR: Polymerase chain reaction; PPV: Positive predictive value; TN: True negative; TP: True positive; URTI: Upper respiratory tract infection

Quality assessment

Table S2.4. Quality assessment of included diagnostic accuracy studies using QUADAS-2

Study	Risk of bias				Applicability concerns		
	Patient selection	Index test	Reference standard	Flow & timing	Patient selection	Index test	Reference standard
Granerod	LR	UR	LR	LR	LR	LR	LR
Hansen	LR	UR	UR	UR	LR	LR	LR
Basaran	LR	UR	UR	UR	LR	LR	LR
Ciftci	LR	UR	UR	LR	LR	LR	LR

LR = Low risk; UR = Unclear risk

3. In adults with suspected encephalitis, does LP before imaging result in deterioration or worse clinical outcome compared to no LP before imaging?

3a. What are the contraindications for an immediate LP?

Evidence summary

Of 278 identified by the search, no article met the inclusion criteria for PICO 3 (**Figure S3.1**). There is evidence from studies that yield of pathogens from CSF declines with anti-infective therapy, so performing LP as early as possible is advisable to aid in making an accurate diagnosis (8). It is also well documented that not all patients with suspected central nervous system (CNS) infection require brain imaging to ensure safety of LP (9). Glimåker et al. stated that LP without prior CT scan is associated with significantly earlier treatment of acute bacterial meningitis and a favourable outcome (10).

Four studies were included for PICO 3a (11–14). See **Table S3.1** for characteristics of included studies. Two previous guidelines did not cite any primary evidence and based their recommendations on prevalent clinical guidance in the UK and USA (11,14–18). Gaieski et al. propose that CT should be performed before LP if there is altered mental status, focal neurological deficits, papilledema, new onset seizures, history of neurological disease, or immunosuppression (11). Solomon et al. propose that contraindications to immediate LP include focal neurological signs, presence of papilloedema, continuous or uncontrolled seizures, or GCS <12 (14). A UK case series on the management of suspected HSV encephalitis over a 12-month period reported that only one patient of 15 who underwent CT prior to LP had an adequate reason for this according to clinical guidelines (12). In an interventional study which evaluated the implementation of a LP pack to guide clinicians on appropriate investigation of suspected CNS infection, contraindications to LP included reduced GCS ≤13 or declining >2, focal neurological weakness, new-onset seizures, systemic shock, coagulation disorder, known severe immunocompromise, skin infection at LP site, or signs of brain shift (13). The WG also felt it relevant to incorporate suggestions from the ESCMID guideline on the diagnosis and treatment of bacterial meningitis (19).

Search string

(("infectious encephalitis"[MeSH Terms] OR "encephalit*"[Title/Abstract]) AND ("spinal puncture"[MeSH Terms] OR "spinal puncture"[MeSH Terms] OR "spinal puncture"[MeSH Terms] OR "lumbar puncture"[Title/Abstract] OR "spinal tap"[Title/Abstract] OR "spinal puncture"[Title/Abstract] OR "centesis"[Title/Abstract])) AND (2008:2021[pdat])

Flowchart

Figure S3.1. Flow chart for study inclusion for PICO 3/3a

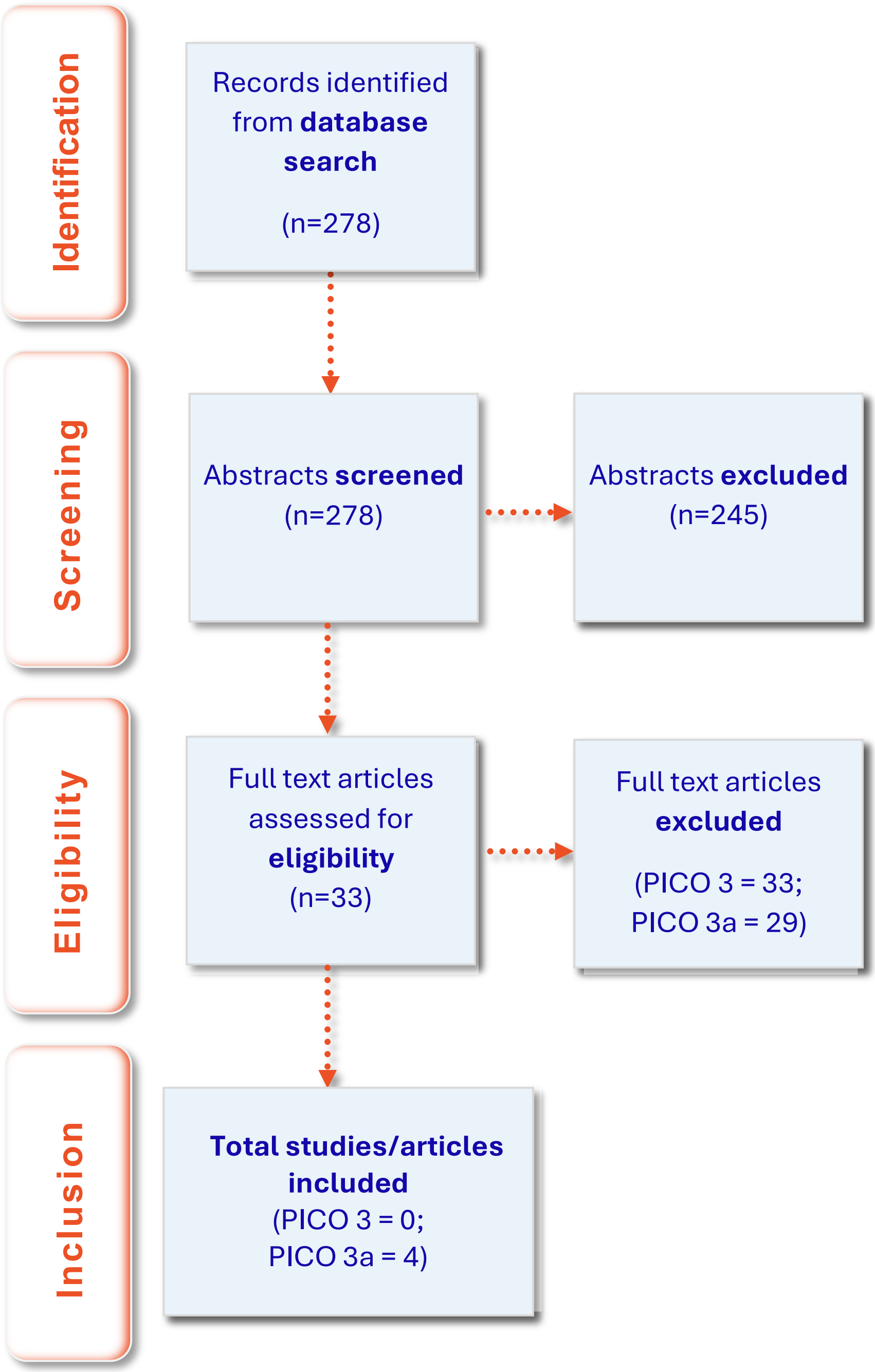

Characteristics of included studies

Table S3.1. Characteristics of included studies for PICO 3a

First author	Study period	Country	Study design	Recruitment method	Number of participants
Gaieski DF	2012	US	Review	NA	N
Michael B	December 2009–May 2010, and June–November 2010	UK	Interventional (but not RCT)	Retrospective	177 LPs performed on 168 patients (93 before and 84 after introduction of the LP pack)
Bell DJ	June 2003 to May 2004	UK	Case series	Retrospective	21 (LP performed in 17: 2 LP before imaging and 15 no LP before imaging)
Ellul M, Solomon T	2018	UK	Review	NA	NA

LP: Lumbar puncture; NA: Not applicable; RCT: Randomised controlled trial; UK: United Kingdom

4. In adults with suspected encephalitis and a first non-diagnostic LP, does a second LP 48 hours later result in improved aetiological diagnosis?

Evidence summary

No article met the inclusion criteria of 278 articles identified by the search (**Figure S4.1**). The evidence supporting this statement mostly relates to HSV encephalitis, in which a first PCR, usually within 3 days of onset of the neurological illness, may be negative, whereas a repeat PCR is positive (20,21). In addition, evolution of CSF parameters such as differential leukocyte count may be useful in diagnosis of other pathogens, e.g. enteroviruses and *M. tuberculosis* may cause an initial predominance of polymorphonuclear cells (polymorphs) which switches to mononuclear (lymphocyte) predominance later in the illness (22).

Search string

(("infectious encephalitis"[MeSH Terms] OR "encephalit*"[Title/Abstract]) AND ("spinal puncture"[MeSH Terms] OR "spinal puncture"[MeSH Terms] OR "spinal puncture"[MeSH Terms] OR "lumbar puncture"[Title/Abstract] OR "spinal tap"[Title/Abstract] OR "spinal puncture"[Title/Abstract] OR "centesis"[Title/Abstract])) AND (2008:2021[pdat])

Flowchart

Figure S4.1. Flow chart for study inclusion for PICO 4

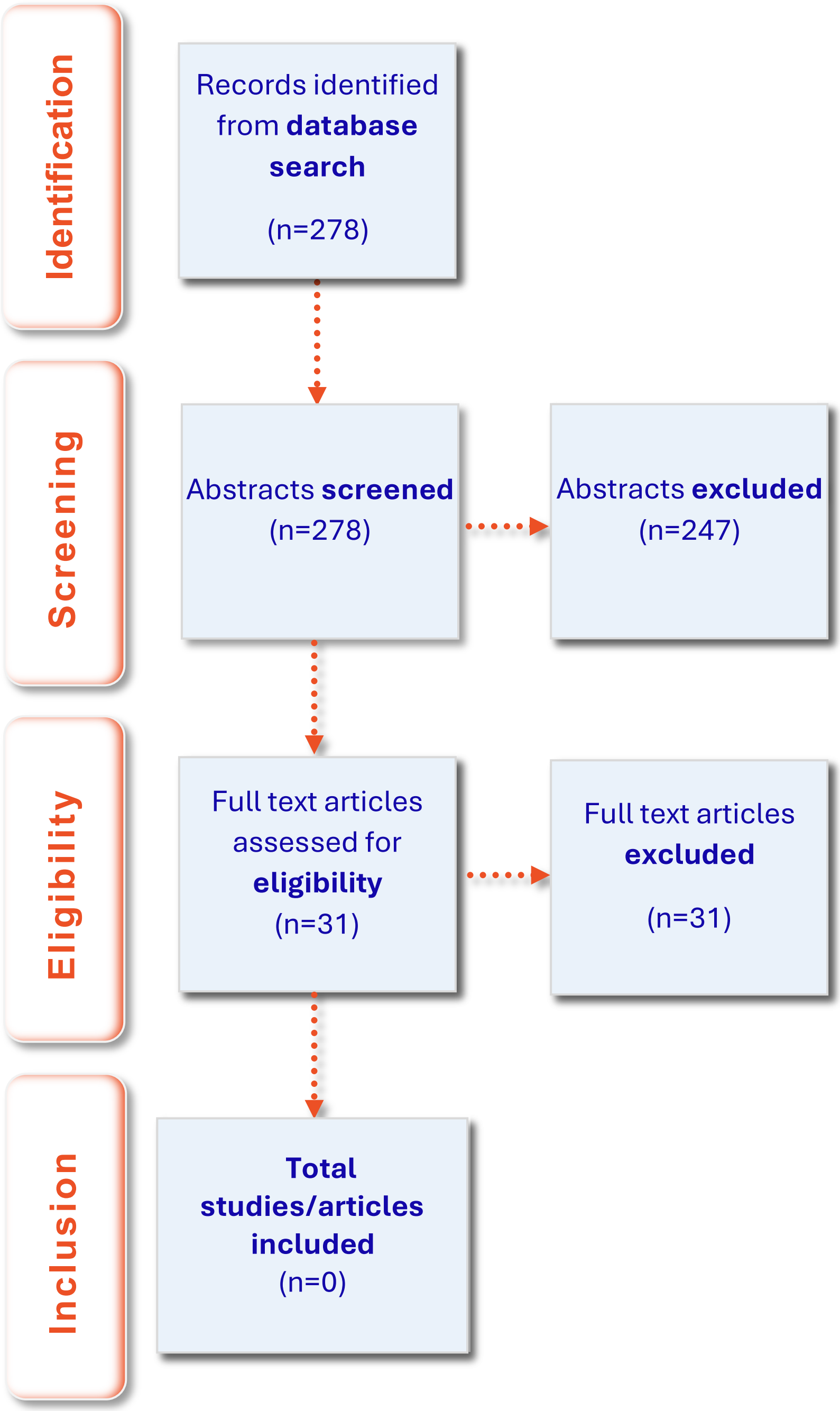

5/5b. In suspected sporadic infectious encephalitis is individualised/specific CSF PCR better than standardised/multiplex CSF PCR panel for first-line detection of the causative pathogen? Are there additional specific testing panels for immunocompromised patients?

5a. Which CSF antibody testing is useful in the diagnosis of infectious encephalitis?

Evidence summary

PICO 5/5b:

Of 674 studies identified by the original search, two cross-sectional studies met the inclusion criteria (23,24). A further literature review/meta-analysis was subsequently identified and included (**Figure S5.1**) (25). See **Table S5.1** for characteristics of included studies. Trujillo-Gomez et al. reported a diagnostic accuracy study of the FilmArray ME panel and has been discussed above (25). For summary of findings tables see PICO 5 supplementary, **Tables S5.2-S5.5**. Broadhurst et al. reported a single-centre's experience of using the same test, however the study did not fit the inclusion criteria for the meta-analysis as it restricted testing to patients with CSF pleocytosis (23). The per-patient yield of the FilmArray ME panel increased from an estimated 11.5% (53/459) positivity rate with no testing restriction to 18.6% (49/263) with criteria (i.e., CSF pleocytosis). The study was limited by a small sample size. Govekar et al. evaluated an in-house multiplex PCR assay, the Syndrome Evaluation System (SES) that detects 22 pathogens in CSF (24). The SES in this validation detected pathogens in 83/130 (63.8%) of acute encephalitis syndrome cases which were negative by the conventional assay. There was high risk of bias for some domains, particularly regarding flow and timing and patient selection in the Broadhurst et al. and Govekar et al. studies (**Table S5.6**) (23,24).

PICO 5a:

Four articles were identified that met the inclusion criteria for the PICO (**Figure S5.2** and **Table S5.1**) (26–29). Forsgren et al. demonstrated intrathecal HSV antibody synthesis in 47 of 53 HSV encephalitis patients at admission who were enrolled in the Swedish randomised controlled trial of aciclovir versus vidarabine. HSV antibody was detected in a further four patients 2-9 days following admission and one more patient >10 days after admission; in total 52 of 53 (98.1%) cases had detectable HSV antibody production (29,30). High risk of bias was identified with regards to flow and timing in this study (**Table S5.6**). A case series

from Belgium detected VZV antibodies in four out of five CSF samples from patients with confirmed VZV, including admission samples and subsequent CSF samples days 1-120 following admission (26). Reusken et al. detected anti-TBEV IgM and IgG antibodies in 100% (8/8) and 87.5% (7/8) of CSF samples, respectively, in patients infected in different areas of Europe who presented with neurological signs of tick-borne encephalitis (28). Encephalitis patients recruited as part of an English prospective study were retrospectively tested for intrathecal antibodies (27). An aetiological cause was identified for five cases through intrathecal antibody testing, including three HSV cases where the CSF PCR was negative, Mycoplasma-specific intrathecal IgG production in one patient with acute disseminated encephalomyelitis, and subacute sclerosing panencephalitis (SSPE) confirmed by demonstrating intrathecal production of measles-specific IgG.

Evidence for the value of anti-VZV IgG testing in addition to VZV CSF PCR is relatively lacking. As mentioned above, one small study reported concurrent positivity by PCR and IgG antibody testing in all patients tested (26), but another found that none of seven patients with a positive VZV PCR had intrathecal production of anti-VZV IgG (27). CSF and serum testing for HSV-specific IgG antibodies can increase overall diagnosis of HSV encephalitis, including when PCR for HSV is negative (27). Diagnosis can be made long after the onset of HSV encephalitis since intrathecal antibodies can persist for many years (31). For SSPE, serological detection of IgG antibodies in CSF has long been the diagnostic gold standard. While for SSPE an absolute threshold for CSF IgG titres is established, for lower titres, and for other viruses, confirmation of intrathecal antibody production by comparing titres in serum versus in CSF is important, since small amounts of IgG can transfer across the blood-CSF barrier (32). In flavivirus encephalitis, while CSF IgM detection can be diagnostic, the interpretation can be challenging due to cross-reactivity between different flaviviruses (33). For TBEV, CSF IgM and IgG testing is also of value for diagnosing breakthrough infection in previously vaccinated individuals, if interpretation of serum antibody results is difficult (e.g., equivocal results).

Search string

For PICO5/5b:

(("infectious encephalitis"[MeSH Terms] OR "encephalitis"[Title/Abstract]) AND ("cerebrospinal fluid"[MeSH Terms] OR "CSF"[Title/Abstract] OR "spinal fluid"[Title/Abstract]) AND ("pcr"[Title/Abstract] OR "polymerase chain reaction"[Title/Abstract] OR "multiplex polymerase chain reaction"[MeSH Terms] OR "multiplex pcr"[MeSH Terms])) AND (2008:2021[pdat])

For 5a:

(("encephalit*"[Title/Abstract] OR "infectious encephalitis"[MeSH Terms]) AND (("cerebrospinal fluid"[MeSH Terms] OR "CSF"[Title/Abstract] OR "spinal fluid"[Title/Abstract]) AND ("oligoclonal bands"[MeSH Terms] OR "oligoclonal

band"[Title/Abstract] OR "antibody test*"[Title/Abstract] OR "intrathecal antibody"[Title/Abstract]))) AND (2008:2021[pdat])

Flowchart

Figure S5.1. Flow chart for study inclusion for PICO 5/5b

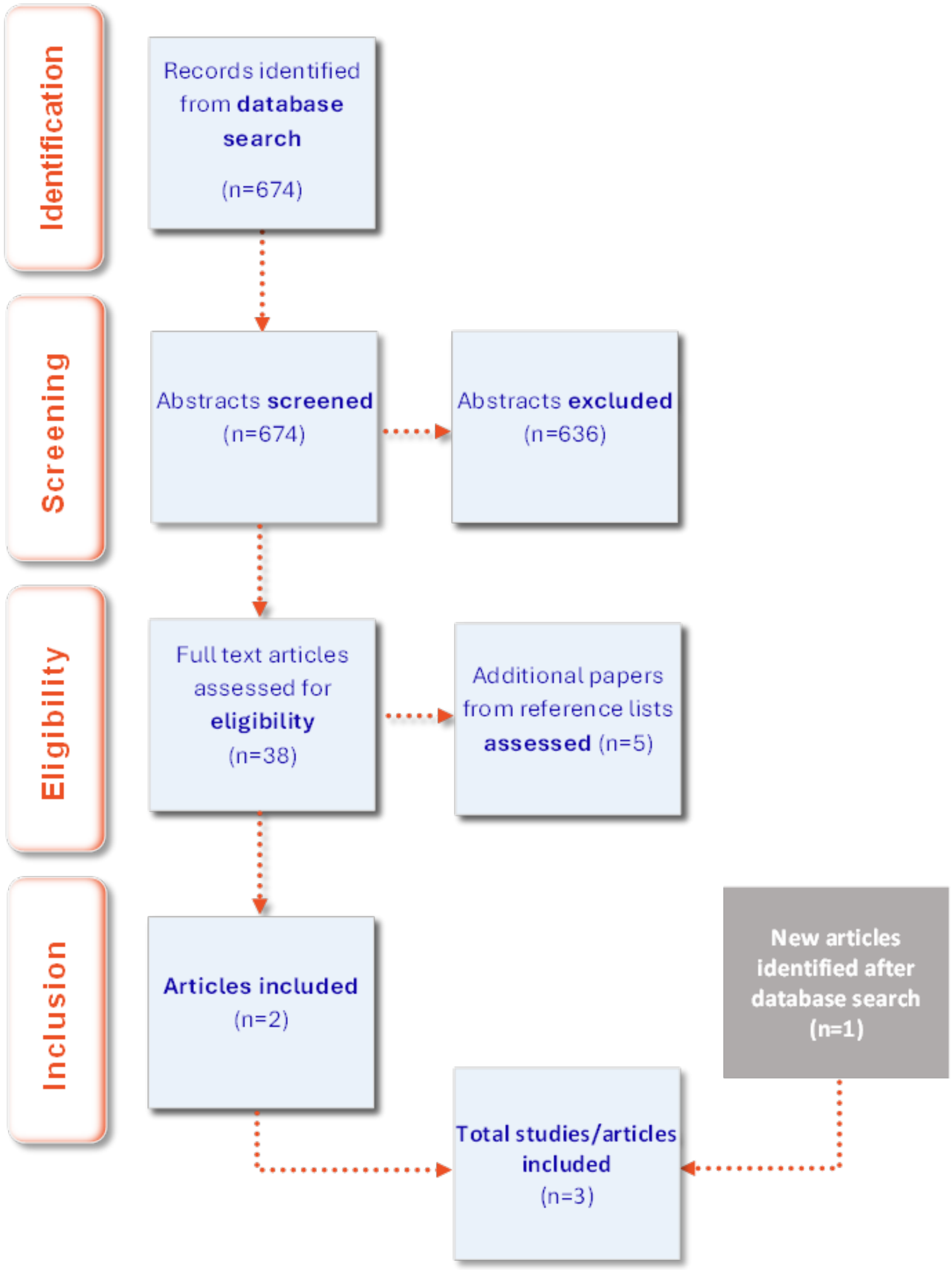

Figure S5.2. Flow chart for study inclusion for PICO 5a

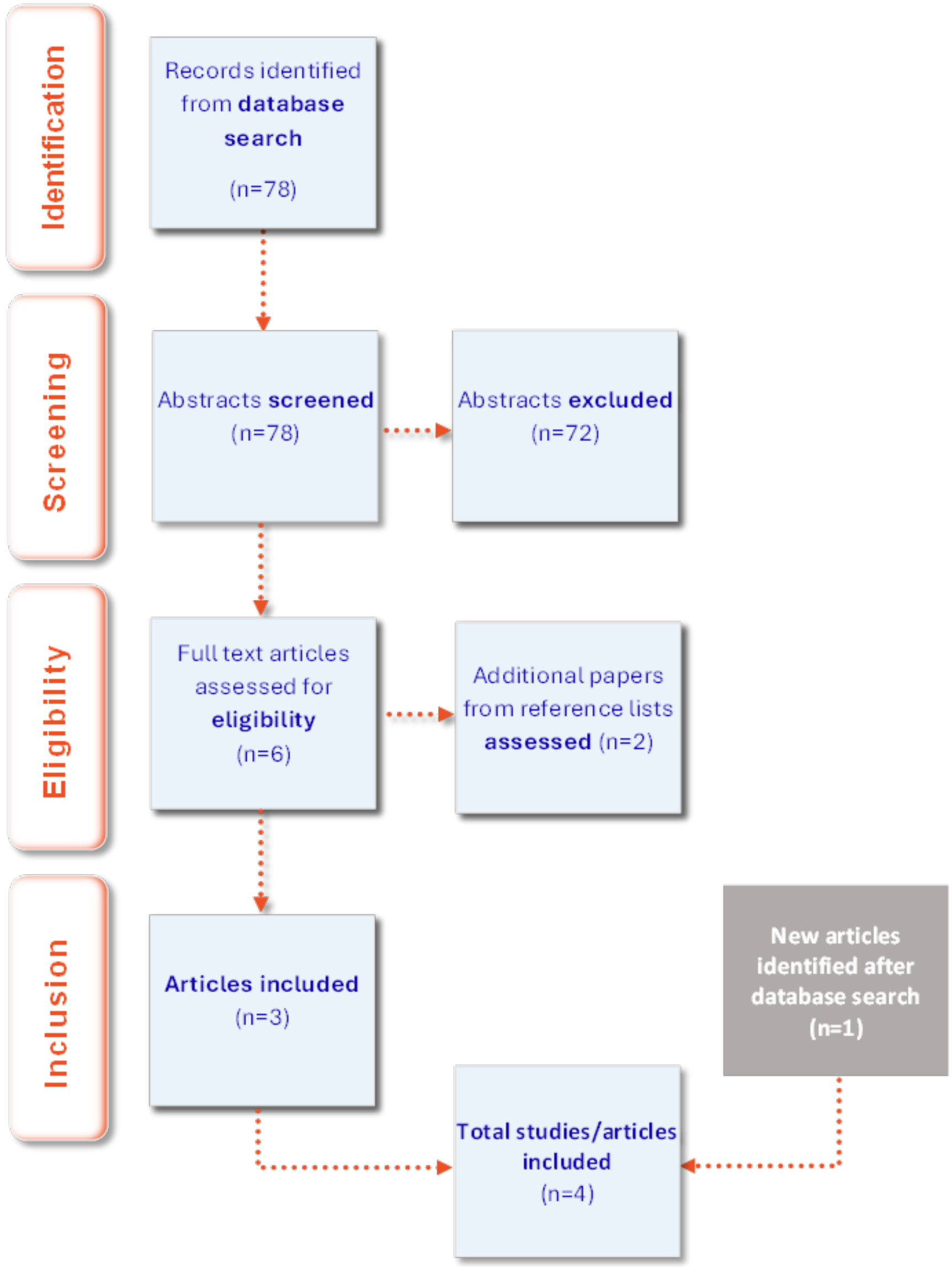

Characteristics of included studies

Table S5.1. Characteristics of included studies for PICOs 5/5b and 5a

First author	Study period	Country	Study design	Recruitment method
**PICO 5/5b**				
Trujillo-Gomez	2021	Spain	Systematic review and meta-analysis - Diagnostic accuracy study	Retrospective & prospective
Broadhurst	2017- 2019	USA	Cross-sectional	Retrospective
Govekar	2019	India	Cross-sectional	Retrospective
**PICO 5a**				
Forsgren	1989	Sweden	RCT	Prospective
Gregorie	1993-2004	Belgium	Case series	Retrospective
Reusken	2010-2017	Netherlands, Finland, Slovenia	Cohort/ cross-sectional	Retrospective
Ambrose	2005-2006	UK	Cohort	Retrospective analysis of prospectively recruited patients

RCT: Randomised controlled trial; UK: United Kingdom; USA: United States of America

Summary of findings tables

Table S5.2. Question: Should BioFire FilmArray ME be used to diagnose HSV-1 infection in patients with suspected encephalitis?

Sensitivity	0.76 (95% CI: 0.51 to 0.90)
Specificity	1.00 (95% CI: 0.95 to 1.00)

Prevalences	19%	10%	30%

Outcome	№ of studies (№ of patients)	Study design	Factors that may decrease certainty of evidence					Effect per 1,000 patients tested			Test accuracy CoE
			Risk of bias	Indirectness	Inconsistency	Imprecision	Publication bias	pre-test probability of19%	pre-test probability of10%	pre-test probability of30%	
**True positives** (patients with HSV-1 infection)	3 studies 6883 patients	cross-sectional (cohort type accuracy study)	serious[a]	serious[b]	not serious	serious[c]	none	143 (97 to 171)	76 (51 to 90)	226 (154 to 270)	⊕◯◯◯ Very low
**False negatives** (patients incorrectly classified as not having HSV-1 infection)								47 (19 to 93)	24 (10 to 49)	74 (30 to 146)	
**True negatives** (patients	3 studies	cross-sectional	serious[a]	serious[b]	not serious	not serious	none	809 (767 to 810)	899 (852 to 900)	699 (663 to 700)	

Outcome	№ of studies (№ of patients)	Study design	Factors that may decrease certainty of evidence					Effect per 1,000 patients tested			Test accuracy CoE
			Risk of bias	Indirectness	Inconsistency	Imprecision	Publication bias	pre-test probability of19%	pre-test probability of10%	pre-test probability of30%	
without HSV-1 infection)	6883 patients	(cohort type accuracy study)									⨁⨁◯◯ Low
**False positives** (patients incorrectly classified as having HSV-1 infection)								1 (0 to 43)	1 (0 to 48)	1 (0 to 37)	

Explanations

a. Downgraded by one level for serious risk of bias, due to Lindstrom (contributing >60% of patients) having a high risk of bias for the index test domain, and Leber having unclear risk of bias for patient selection.

b. Downgraded by one level for serious indirectness, due to inclusion of patients with meningitis and of children

c. Downgraded by one level for serious imprecision, due to wide CIs (individual studies and pooled), and very disparate point estimates from the 3 studies.

Table S5.3. Question: Should BioFire FilmArray ME be used to diagnose HSV-2 infection in patients with suspected encephalitis?

Sensitivity	0.94 (95% CI: 0.84 to 0.98)
Specificity	1.00 (95% CI: 1.00 to 1.00)

Prevalences	19%	10%	30%

Outcome	№ of studies (№ of patients)	Study design	Factors that may decrease certainty of evidence					Effect per 1,000 patients tested			Test accuracy CoE
			Risk of bias	Indirectness	Inconsistency	Imprecision	Publication bias	pre-test probability of19%	pre-test probability of10%	pre-test probability of30%	
**True positives** (patients with HSV-2 infection)	3 studies 6883 patients	cross-sectional (cohort type accuracy study)	serious [a]	serious[b]	not serious	not serious	none	179 (159 to 187)	94 (84 to 98)	283 (252 to 295)	⨁⨁◯◯ Low
**False negatives** (patients incorrectly classified as not having HSV-2 infection)								11 (3 to 31)	6 (2 to 16)	17 (5 to 48)	
**True negatives** (patients without	3 studies 6883 patients	cross-sectional (cohort	serious [a]	serious[b]	not serious	not serious	none	809 (808 to 810)	899 (897 to 900)	699 (698 to 700)	⨁⨁◯◯ Low

Outcome	№ of studies (№ of patients)	Study design	Factors that may decrease certainty of evidence					Effect per 1,000 patients tested			Test accuracy CoE
			Risk of bias	Indirectness	Inconsistency	Imprecision	Publication bias	pre-test probability of19%	pre-test probability of10%	pre-test probability of30%	
HSV-2 infection)		type accuracy study)									
**False positives** (patients incorrectly classified as having HSV-2 infection)								1 (0 to 2)	1 (0 to 3)	1 (0 to 2)	

Explanations

a. Downgraded by one level for serious risk of bias, due to Lindstrom (contributing >60% of patients) having a high risk of bias for the index test domain, and Leber having unclear risk of bias for patient selection.

b. Downgraded by one level for serious indirectness, due to inclusion of patients with meningitis and of children

Table S5.4. Question: Should BioFire FilmArray ME be used to diagnose VZV infection in patients with suspected encephalitis?

Sensitivity	0.91 (95% CI: 0.79 to 0.97)
Specificity	1.00 (95% CI: 0.99 to 1.00)

Prevalences	19%	10%	30%

Outcome	№ of studies (№ of patients)	Study design	Factors that may decrease certainty of evidence					Effect per 1,000 patients tested			Test accuracy CoE
			Risk of bias	Indirectness	Inconsistency	Imprecision	Publication bias	pre-test probability of19%	pre-test probability of10%	pre-test probability of30%	
**True positives** (patients with VZV infection)	4 studies 6897 patients	cross-sectional (cohort type accuracy study)	serious [a]	serious [b]	not serious	serious [c]	none	174 (150 to 184)	91 (79 to 97)	274 (237 to 291)	⨁◯◯◯ Very low
**False negatives** (patients incorrectly classified as not having VZV infection)								16 (6 to 40)	9 (3 to 21)	26 (9 to 63)	
**True negatives** (patients without VZV infection)	4 studies 6897 patients	cross-sectional (cohort type	serious [a]	serious [b]	not serious	not serious	none	808 (799 to 810)	898 (888 to 900)	699 (691 to 700)	⨁⨁◯◯ Low

Outcome	№ of studies (№ of patients)	Study design	Factors that may decrease certainty of evidence					Effect per 1,000 patients tested			Test accuracy CoE
			Risk of bias	Indirectness	Inconsistency	Imprecision	Publication bias	pre-test probability of19%	pre-test probability of10%	pre-test probability of30%	
**False positives** (patients incorrectly classified as having VZV infection)		accuracy study)						2 (0 to 11)	2 (0 to 12)	1 (0 to 9)	

Explanations

a. Downgraded by one level for serious risk of bias, due to Lindstrom (contributing >60% of patients) having a high risk of bias for the index test domain, and Leber having unclear risk of bias for patient selection.

b. Downgraded by one level for serious indirectness, due to inclusion of patients with meningitis and of children

c. Downgraded by one level for serious imprecision, due to CI crossing below 80% for sensitivity

Table S5.5. Question: Should BioFire FilmArray ME be used to diagnose enterovirus infection in patients with suspected encephalitis?

Sensitivity	0.94 (95% CI: 0.87 to 0.97)
Specificity	0.99 (95% CI: 0.99 to 1.00)

Prevalences	19%	10%	30%

Outcome	№ of studies (№ of patients)	Study design	Factors that may decrease certainty of evidence					Effect per 1,000 patients tested			Test accuracy CoE
			Risk of bias	Indirectness	Inconsistency	Imprecision	Publication bias	pre-test probability of19%	pre-test probability of10%	pre-test probability of30%	
**True positives** (patients with enterovirus infection)	3 studies 6883 patients	cross-sectional (cohort type accuracy study)	serious [a]	serious [b]	not serious	not serious	none	178 (165 to 185)	94 (87 to 97)	281 (261 to 292)	⊕⊕◯◯ Low
**False negatives** (patients incorrectly classified as not having enterovirus infection)								12 (5 to 25)	6 (3 to 13)	19 (8 to 39)	
**True negatives** (patients	3 studies	cross-sectional (cohort	serious [a]	serious [b]	not serious	not serious	none	804 (799 to 808)	894 (888 to 897)	695 (691 to 698)	

Outcome	№ of studies (№ of patients)	Study design	Factors that may decrease certainty of evidence					Effect per 1,000 patients tested			Test accuracy CoE
			Risk of bias	Indirectness	Inconsistency	Imprecision	Publication bias	pre-test probability of19%	pre-test probability of10%	pre-test probability of30%	
without enterovirus infection)	6883 patients	type accuracy study)									⨁⨁◯◯ Low
**False positives** (patients incorrectly classified as having enterovirus infection)								6 (2 to 11)	6 (3 to 12)	5 (2 to 9)	

Explanations

a. Downgraded by one level for serious risk of bias, due to Lindstrom (contributing >60% of patients) having a high risk of bias for the index test domain, and Leber having unclear risk of bias for patient selection.

b. Downgraded by one level for serious indirectness, due to inclusion of patients with meningitis and of children

Quality assessment

Table S5.6. Quality assessment of included diagnostic accuracy studies using QUADAS-2

Study	Risk of bias				Applicability concerns		
	Patient selection	Index test	Reference standard	Flow & timing	Patient selection	Index test	Reference standard
**PICO 5/5b**							
Braodhurst	LR	LR	LR	HR	UR	LR	UR
Govekar	HR	UR	UR	HR	LR	UR	LR
**PICO 5a**							
Reusken	UR	LR	LR	UR	LR	LR	LR
Gregorie	LR	UR	LR	LR	LR	UR	LR
Forsgren	LR	LR	UR	HR	LR	LR	UR
Ambrose	UR	UR	LR	LR	LR	LR	LR

HR = High risk; LR = Low risk; UR = Unclear risk

Table S5.7. Quality assessment of Trujillo-Gomez et al. using PRISMA checklist

Section/topic	Number	PRISMA-DTA checklist item	Reported on page number
**TITLE / ABSTRACT**			
Title	1	Identify the report as a systematic review (+/- meta-analysis) of diagnostic test accuracy (DTA) studies.	1
Abstract	2	Abstract: See PRISMA-DTA for abstracts.	1
**INTRODUCTION**			
Rationale	3	Describe the rationale for the review in the context of what is already known.	2
Clinical role of index test	D1	State the scientific and clinical background, including the intended use and clinical role of the index test, and if applicable, the rationale for minimally acceptable test accuracy (or minimum difference in accuracy for comparative design).	2
Objectives	4	Provide an explicit statement of question(s) being addressed in terms of participants, index test(s), and target condition(s).	2
**METHODS**			
Protocol and registration	5	Indicate if a review protocol exists, if and where it can be accessed (e.g., Web address), and, if available, provide registration information including registration number.	2
Eligibility criteria	6	Specify study characteristics (participants, setting, index test(s), reference standard(s), target condition(s), and study design) and report characteristics (e.g., years considered, language, publication status) used as criteria for eligibility, giving rationale.	2-3; no rationale given in manuscript text
Information sources	7	Describe all information sources (e.g., databases with dates of coverage, contact with study authors to identify additional studies) in the search and date last searched.	2
Search	8	Present full search strategies for all electronic databases and other sources searched, including any limits used, such that they could be repeated.	Supplementary Material Appendix 1
Study selection	9	State the process for selecting studies (i.e., screening, eligibility, included in systematic review, and, if applicable, included in the meta-analysis).	3
Data collection process	10	Describe method of data extraction from reports (e.g., piloted forms, independently, in duplicate) and any processes for obtaining and confirming data from investigators.	3
Definitions for data extraction	11	Provide definitions used in data extraction and classifications of target condition(s), index test(s), reference standard(s) and other characteristics (e.g. study design, clinical setting).	3-4
Risk of bias and	12	Describe methods used for assessing risk of bias in individual studies and concerns regarding the applicability to	3

applicability		the review question.	
Diagnostic accuracy measures	13	State the principal diagnostic accuracy measure(s) reported (e.g. sensitivity, specificity) and state the unit of assessment (e.g. per-patient, per-lesion).	3
Synthesis of results	14	Describe methods of handling data, combining results of studies and describing variability between studies. This could include but is not limited to: a) handling of multiple definitions of target condition. b) handling of multiple thresholds of test positivity, c) handling multiple index test readers, d) handling of indeterminate test results, e) grouping and comparing tests, f) handling of different reference standards	3-4
Meta-analysis	D2	Report the statistical methods used for meta-analyses, if performed.	3
Additional analyses	16	Describe methods of additional analyses (e.g., sensitivity or subgroup analyses, meta-regression), if done, indicating which were pre-specified.	3,5
**RESULTS**			
Study selection	17	Provide numbers of studies screened, assessed for eligibility, included in the review (and included in meta-analysis, if applicable) with reasons for exclusions at each stage, ideally with a flow diagram.	5
Study characteristics	18	For each included study provide citations and present key characteristics including: a) participant characteristics (presentation, prior testing), b) clinical setting, c) study design, d) target condition definition, e) index test, f) reference standard, g) sample size, h) funding sources	Supplementary Material Appendix 4
Risk of bias and applicability	19	Present evaluation of risk of bias and concerns regarding applicability for each study.	6
Results of individual studies	20	For each analysis in each study (e.g. unique combination of index test, reference standard, and positivity threshold) report 2x2 data (TP, FP, FN, TN) with estimates of diagnostic accuracy and confidence intervals, ideally with a forest or receiver operator characteristic (ROC) plot.	Supplementary Material Appendix 6 & 7
Synthesis of results	21	Describe test accuracy, including variability; if meta-analysis was done, include results and confidence intervals.	6-9, Supplementary Material Appendix 6
Additional analysis	23	Give results of additional analyses, if done (e.g., sensitivity or subgroup analyses, meta-regression; analysis of index test: failure rates, proportion of inconclusive results, adverse events).	8,15
**DISCUSSION**			
Summary of evidence	24	Summarize the main findings including the strength of evidence.	From 9
Limitations	25	Discuss limitations from included studies (e.g. risk of bias and concerns regarding applicability) and from the review process (e.g. incomplete retrieval of identified research).	10,13

Conclusions	26	Provide a general interpretation of the results in the context of other evidence. Discuss implications for future research and clinical practice (e.g. the intended use and clinical role of the index test).	12-13
**FUNDING**			
Funding	27	For the systematic review, describe the sources of funding and other support and the role of the funders.	14

FN = False negative; FP = False positive; TN = True negative; TP = True positive

6. In adults with suspected infectious encephalitis and no aetiological diagnosis despite extensive investigation, who are deteriorating, does brain biopsy change patient management compared to no brain biopsy?

Evidence summary

The initial search identified 413 studies, of which two case series met the inclusion criteria (**Figure S6.1**) (34,35). In 14 US patients with brainstem encephalitis and undefined aetiology who underwent biopsy, the underlying pathology was revealed in seven patients, all of whom were diagnosed with inflammatory/ autoimmune conditions (34). The other seven patients had brain biopsies that did not point to a specific diagnosis; six of these biopsies revealed non-specific inflammatory changes with a lack of evidence of an infectious process. Following brain biopsy, immunotherapy was either initiated or escalated in 9 of the 14 patients. Abdullah et al. retrospectively identified 59 US patients who underwent open craniotomy for undiagnosed encephalitis (35). Biopsy confirmed the preoperative diagnosis in 46% of cases, refuted the preoperative leading diagnosis in 25% of cases, and remained inconclusive in 29% of cases. The major complication rate was 23%. Characteristics of the included studies and quality assessment are provided in **Tables S6.1** and **S6.2**, respectively.

In summary, brain biopsy has been proposed to improve diagnosis of encephalitis in smaller case series or cohort studies, some of which focus on specific types of encephalitis, e.g. that of the brainstem. Not all studies report on actual (versus potential) change in management, and a comparison with those not having biopsy is not made in most studies. This means the overall evidence is indirect, so the WG felt unable to make a recommendation, instead making a good practice statement. While it carries the risk of potential serious harm, and some diagnoses may not be better made following biopsy, the WG decided that in patients who are deteriorating without a diagnosis despite extensive investigations for causes of encephalitis, some would benefit from brain biopsy.

Search string

(("infectious encephalitis"[MeSH Terms] OR "encephalit*"[Title/Abstract]) AND ("brain biopsy"[Title/Abstract] OR "biopsy"[MeSH Terms])) AND (2008:2021[pdat])

Flowchart

Figure S6.1. Flow chart for study inclusion for PICO 6

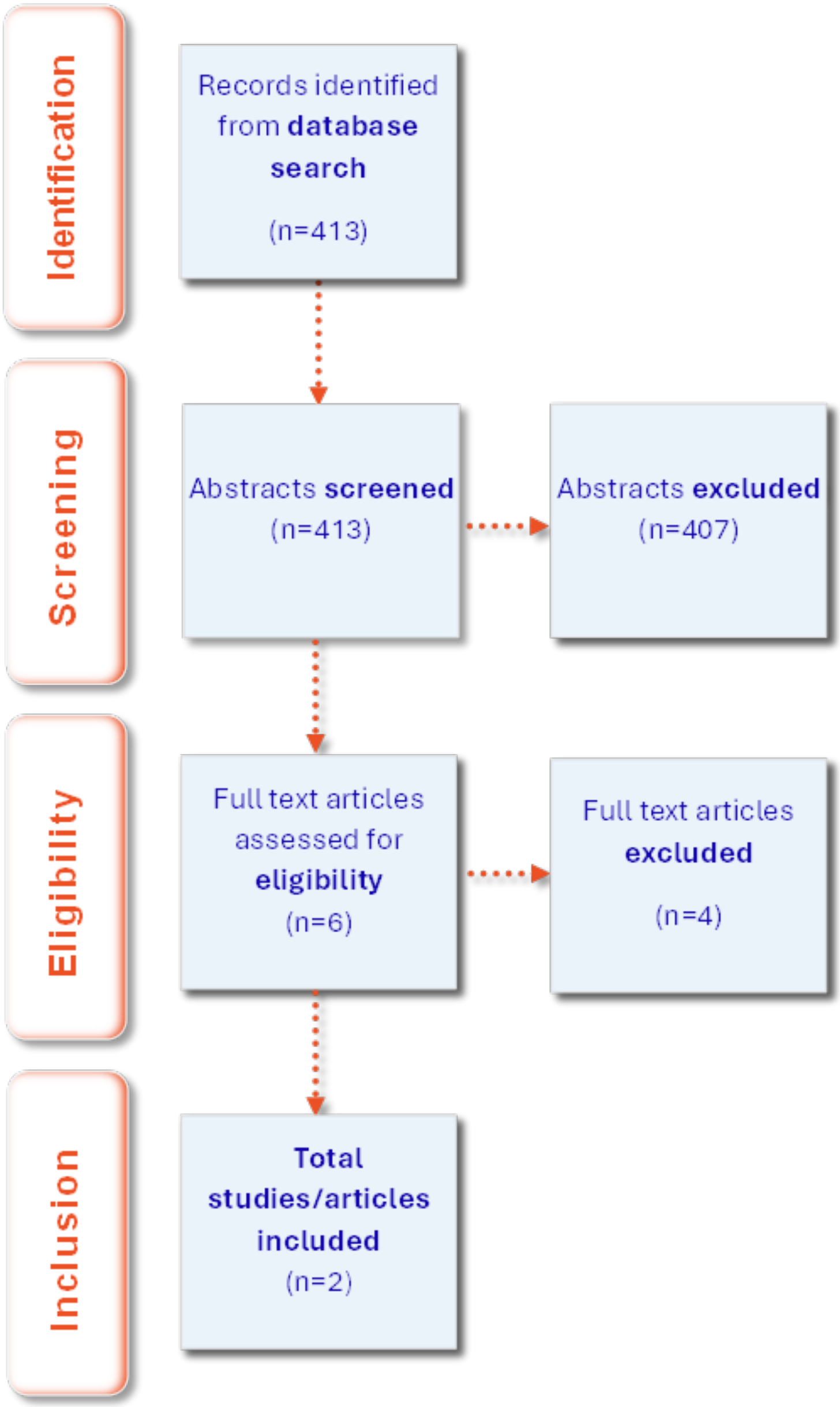

Characteristics of included studies

Table S6.1. Characteristics of included studies for PICO 6

First author	Study period	Country	Study design	Recruitment method
Abdullah	Jan 2009- Dec 2013	USA	Cohort	Retrospective
Tan	Jan 1997- Apr 2010	USA	Cohort	Retrospective

USA: United States of America

Quality assessment

Table S6.2. Quality assessment of included diagnostic accuracy studies using JBI case series

	Q1	Q2	Q3	Q4	Q5	Q6	Q7	Q8	Q9	Q10	Total yes
Tan	Y	Y	Y	NA	U	Y	Y	Y	N	Y	7
Abdullah	Y	Y	U	NA	U	Y	N	Y	N	NA	4

JBI: Joanna Briggs Institute; NA = Not applicable; N = No; U = Unclear; Y = Yes

7. In adults with suspected encephalitis, what is the diagnostic accuracy of specific features on CT and MRI to predict an infectious aetiology compared to PCR?

Evidence summary

Of 469 articles identified by the search, six met the inclusion criteria for this PICO, and an additional article was identified after the database search (**Figure S7.1**) (1–3,5,36–38). The included studies came from six countries and comprised a total of 730 individuals (see **Table S7.1** for characteristics and **Table S7.2** for quality assessments of the studies). A retrospective Turkish cohort study (n=67) showed temporal lobe involvement on MRI in 86.7% (13/15) patients with PCR-proven HSV compared to 26.5% (13/49) of patients with no evidence of HSV infection (p<0.001) (1). Involvement of the insular and limbic systems was also more common in HSV encephalitis (53.3% [8/15] versus 10.2% [5/49]; p=0.001). A German study confirmed the presence of typical temporal MRI pathology in 86.7% (13/15) of HSV encephalitis cases (2). It also suggested superiority of DWI to detect very early changes compared to standard T2-weighted images. Temporal abnormalities were also noted in four of five (80%) and six of seven (85.7%) HSV encephalitis cases in a Thai and Turkish study, respectively (3,5). In a French cohort of 349 individuals with infectious encephalitis, the presence of haemorrhage (14.2% versus 2.3%), inflammation (33.1% versus 18%), and temporal lobe signals (42.5% versus 5%) significantly differentiated HSV/VZV encephalitis (n=127) from other infectious causes (n= 222) (38). Individuals with HSV or VZV were also less likely to have MRI brain scans with no brain lesions reported (42.5% versus 78.8%; p<0.001). A British study reported frontal and temporal changes on T2-weighted and FLAIR sequences in nearly all 15 HSV cases; basal ganglia and thalamic abnormalities were rarer (20-38%, depending on the imaging sequence) (37). The sensitivity and specificity of CT imaging for HSV was 83% and 100%, respectively. MRI had a sensitivity of 85% and specificity of 100% for HSV. An Indian study reported the commonest feature in JEV on MRI was thalamic T2 hyperintensity (19/22, 86.4%), brainstem involvement (13/22, 59.1%), and basal ganglia involvement (12/22, 54.5%) (36). All eight patients with HSV had temporal and basi-frontal involvement.

The studies included in this PICO comprised a small number of participants, with substantial heterogeneity in terms of population and comparator diagnoses, so their results were not pooled using meta-analysis. While direct comparisons were not identified, MRI scans are shown to evaluate the brain parenchyma in more detail than CT scans, and therefore are less likely to miss features of encephalitis (39). However, the WG acknowledged that some patients are not clinically stable enough for MRI scanning, especially soon after presenting to hospital. Thus, while some features of encephalitis may be missed, in this situation, a CT scan would be preferable to no imaging, as it can identify alternative diagnoses and, in some cases, confirm the safety of an LP when considered in conjunction with clinical features.

Search string

(("polymerase chain reaction"[MeSH Terms]) OR (PCR[Title/Abstract])) AND ((("infectious encephalitis"[MeSH Terms] OR "encephalit*"[Title/Abstract]) AND ("magnetic resonance imaging"[MeSH Terms] OR "neuroimaging"[MeSH Terms] OR "CT"[Title/Abstract] OR "MRI"[Title/Abstract] OR "imaging"[Title/Abstract] OR "scan"[Title/Abstract] OR "radiolog*"[Title/Abstract])) AND (2008:2021[pdat]))

Flowchart

Figure S7.1. Flow chart for study inclusion for PICO 7

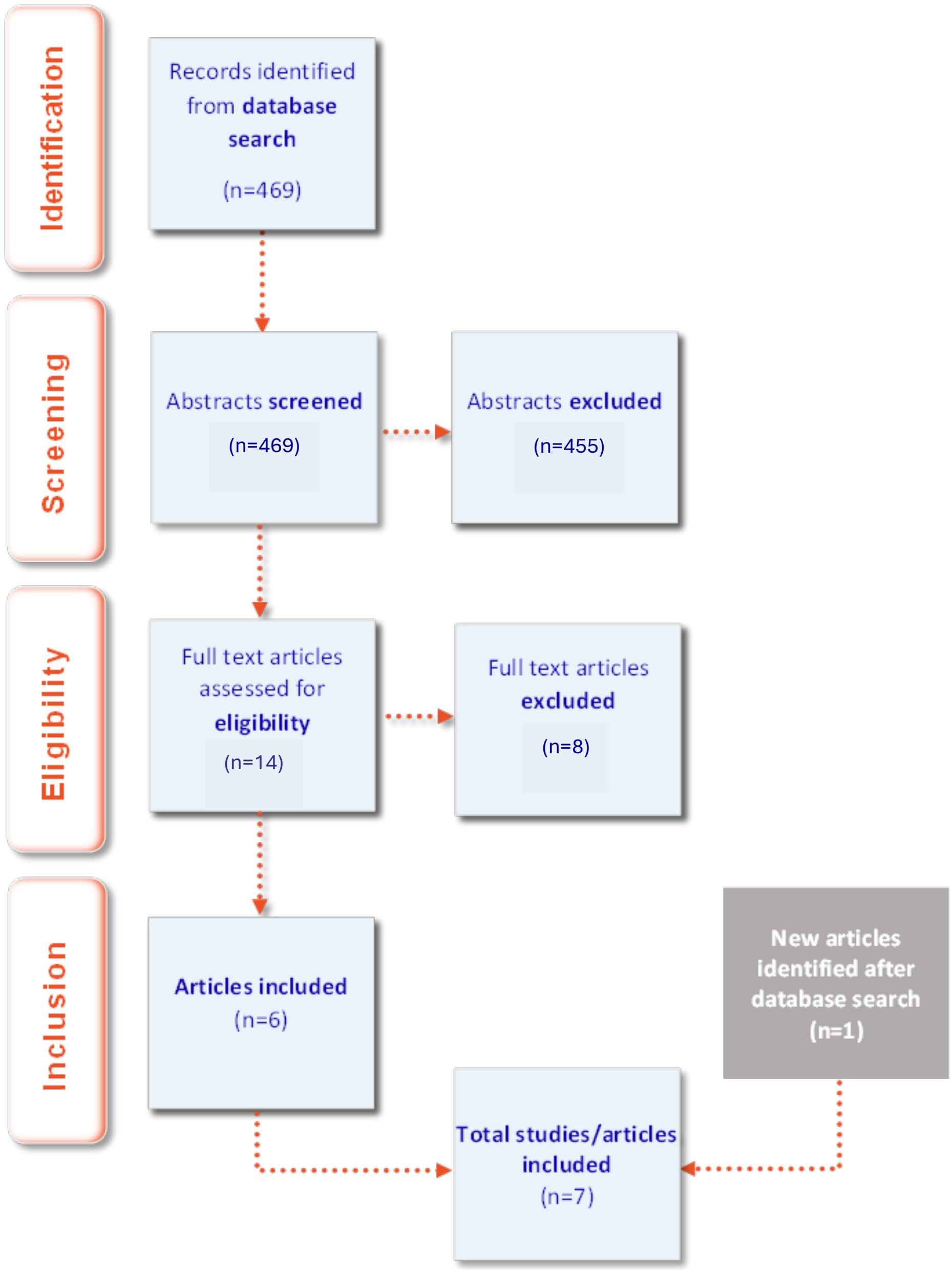

Characteristics of included studies

Table S7.1. Characteristics of included studies for PICO 7

First author	Country	Study period	Study design	Recruitment method	Number of participants
Basaran	Turkey	2011-2017	Cohort	Retrospective	67, including 16 with PCR diagnosed HSV-1 and 51 non-HSV-1
Jordan	Germany	2000-2014	Cohort	Retrospective	49, including 22 with confirmed viral aetiology (15 HSV; 5 VZV; 2 EBV).
Pleumpanupat	Thailand	1997-2006	Case-control	Retrospective	7 HSE cases and 22 controls
Ciftci	Turkey	2010-2013	Cohort	Retrospective	68:<br>7 HSV<br>48 PCR-negative viral encephalitis<br>13 alternative diagnoses
Misra 2010	India	2004-2009	Cohort	Prospective	88 (Note: 25 [28%] aged <15 years):<br>22 JE<br>9 dengue<br>8 herpes<br>2 EBV<br>47 no specific viral diagnosis
Granerod	UK	2005-2008	Cohort	Prospective	85 CT examinations from 68 patients and 101 MRI examinations from 80 patients.<br>11 HSV patients with CT and 11 HSV patients with MRI.
Le Marechal (identified after database search)	France	2016-2018	Cohort	Prospective	349<br>(88 HSV; 39 VZV; 22 TBE; 19 Listeria; 11 flu; 117 unknown)

CT: Computer tomography; EBV: Epstein Barr virus; HSE: Herpes simplex encephalitis; HSV: Herpes simplex virus; JE: Japanese encephalitis; MRI: Magnetic resonance imaging; TBE: Tick-borne encephalitis; UK: United Kingdom; VZV: Varicella zoster virus

Quality assessment

Table S7.2. Quality assessment of included diagnostic accuracy studies using QUADAS-2

Study	Risk of bias				Applicability concerns		
	Patient selection	Index test	Reference standard	Flow & timing	Patient selection	Index test	Reference standard
Granerod	LR	LR	LR	UR	LR	LR	LR
Misra	LR	UR	UR	LR	LR	LR	LR
Jordan	UR	LR	UR	LR	LR	LR	LR
Pleumpanupat	UR	UR	UR	LR	LR	LR	LR
Basaran	LR	UR	UR	UR	LR	LR	LR
Le Marechal	UR	UR	UR	UR	LR	LR	LR
Ciftci	LR	UR	UR	LR	LR	LR	LR

LR = Low risk; UR = Unclear risk

8. In patients with suspected encephalitis and psychiatric features, does EEG change the patient management compared to no EEG?

Evidence summary

343 studies were identified by the search; only one met the inclusion criteria (see **Figure S8.1** for flow diagram, **Table S8.1** for characteristics of study, and **Table S8.2** for quality assessment) (40). This was a prospective cohort of 22 patients with probable limbic encephalitis of unknown aetiology, selected from 78 individuals referred from psychiatry for neurological assessment. Patients with definitive psychiatric illness or confirmed diagnosis of HSV encephalitis were excluded. As a part of neurological assessment, all selected patients underwent EEG, alongside a panel of laboratory tests. EEG was abnormal in 21 of the 22 patients, with features of periodic lateralized epileptiform discharges, diffuse epileptiform discharges, midpositive triphasic waves and non-specific abnormalities (e.g. excessive Theta waves). As a result of the assessment, patients were diagnosed with organic rather than psychiatric disease. This study was small, reported from a single centre outside of Europe, and did not delineate what change in management was due to the results of the EEG versus other aspects of the evaluation by a neurologist (40). However, the WG noted that various diagnoses were made more likely by the EEG results, EEG is known to be a useful test to discriminate features of an organic encephalopathy from a psychiatric diagnosis (15,16), and in suspected cases of non-convulsive status epilepticus, encephalitis may be one of the most common causes (41).

Search string

((((((delusion*[Title/Abstract]) OR (depress*[Title/Abstract])) OR (agitat*[Title/Abstract])) OR (hallucinat*[Title/Abstract])) OR (psych*[Title/Abstract])) OR (neuropsychiatr*[Title/Abstract])) AND (("infectious encephalitis"[MeSH Terms] OR "encephalit*"[Title/Abstract]) AND ("electroencephalography"[MeSH Terms] OR "eeg"[Title/Abstract] OR "electroencephalogr*"[Title/Abstract] OR "electroencephalography"[MeSH Terms] OR "electroencephalography"[MeSH Terms]) AND (2008:2021[pdat]))

Flowchart

Figure S8.1. Flow chart for study inclusion for PICO 8

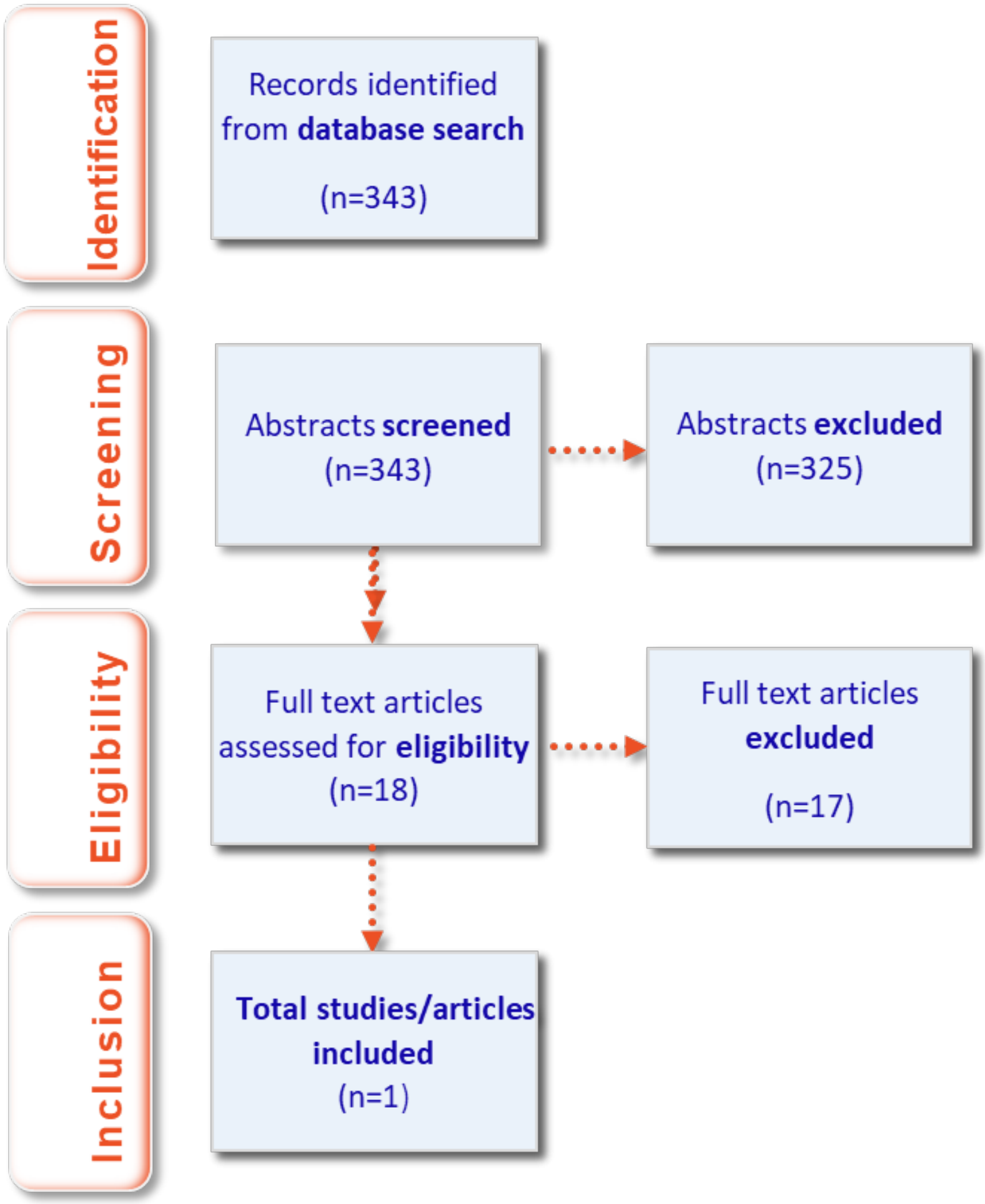

Characteristics of included studies

Table S8.1. Characteristics of included studies for PICO 8

First author	Study period	Country	Study design	Recruitment method	Number of participants
Chandra 2014	Jan 2010-Jan 2011	India	Cohort	Prospective	22 patients with uncertain aetiology of probable limbic encephalitis referred from psychiatry for specialist neurological assessment

Quality assessment

Table S8.2. Quality assessment of Chandra et al. using Newcastle Ottawa scale for cross-sectional studies

	**Total score**	**Selection**				**Comparability**	**Outcome**	
		Sample representativeness	Sample size	Non-respondents	Ascertainment of exposure	Based on design and analysis	Assessment of outcome	Statistical test
Chandra et al.	4	*		*	**			

9. In patients with suspected encephalitis, does treatment with acyclovir improve patient outcome compared to no acyclovir administration?

9a. If HSV is not confirmed when can acyclovir be stopped?

Evidence summary

PICO 9:
One paper met the PICO inclusion criteria of 702 papers retrieved by the search (see **Figure S9.1** for flow diagram, and **Table S9.1** for characteristics and **Table S9.2** for quality assessment of the study) (42). This paper included data from an early randomised trial showing the superiority of aciclovir compared to vidarabine in terms of morbidity and mortality for the treatment of HSE. Mortality was 19% in the aciclovir-treated group compared to 50% in the vidarabine group (p=0.04) (30,42). Some bias concerns were identified due to participants probably being aware of their assigned intervention during the trial; carers and people delivering the interventions being aware of participants' assigned intervention during the trial; and failures in implementing the intervention that could have affected the outcome.

PICO 9a:
No study met the PICO inclusion criteria (**Figure S9.1**). As HSV is the a common cause of infectious encephalitis in adults in Europe (diagnosed in 10-20% of those with suspected encephalitis) (4,43–45), and HSV encephalitis can present with initial normal CSF leukocyte count, negative CSF HSV PCR and normal MRI brain appearances, it was decided that empirical treatment should only be stopped if all of these are present, when the clinical probability is felt to be low (46–48).

Search string

(("infectious encephalitis"[MeSH Terms] OR "encephalit*"[Title/Abstract]) AND ("acyclovir"[Title/Abstract] OR "acyclovir"[MeSH Terms])) AND (2008:2021[pdat])

Flowchart

Figure S9.1. Flow chart for study inclusion for PICO 9 and 9a

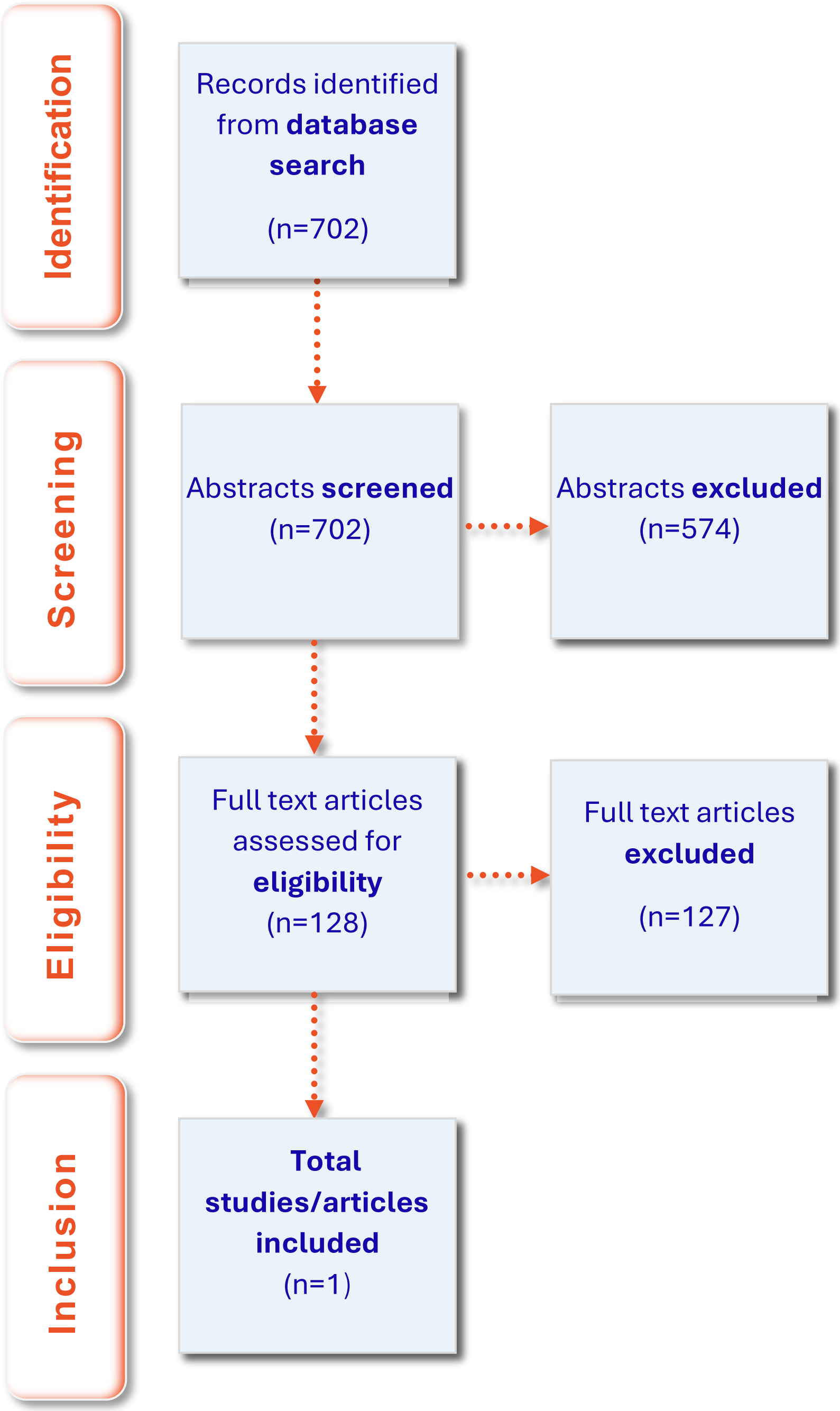

Characteristics of included studies

Table S9.1. Characteristics of included studies for PICO 9

First author	Study period	Country	Study design	Recruitment method	Inclusion criteria	Number of participants	Outcomes
Hjalmarsson	1980s	Sweden	Case series	Retrospective	127 patients with suspected HSE, with CSF data in the Swedish Trial of Aciclovir Database. 53 with virologically confirmed HSE. 5 subsequently excluded.	48 total: 25 treated with aciclovir, 23 treated with vidarabine	Death:<br>Aciclovir group: 3 (12%)<br>Vidarabine group: 11 (48%)<br><br>Expressed as odds ratio per log-unit CSF antibody titre, the reduction of an adverse outcome was 1.09 (95% CI: 0.70–1.71) in the ACV-treated patients and 0.49 (95% CI: 0.19–0.98) in the VIRA-A treated patients. This suggests that the prognosis after ACV treatment is relatively unaffected by the CSF-IgG levels, while the prognosis after VIRA-A treatment improves with increasing antibody levels, approaching the prognosis for ACV-treated patients. These results remained when including potential confounders into the regression model (i.e., days between symptom onset and start of treatment, and level of consciousness on admission).

Quality assessment

Table S9.2. Quality assessment of Hjalmarsson/Skoldenberg using revised Cochrance risk-of-bias tool for randomised trials (RoB 2)

Domain	Risk-of-bias judgment
Risk of bias arising from randomization process	Low
Risk of bias due to deviations from the intended interventions (effect of adhering to intervention)	Some concerns<br>Direction of bias: Favours comparator
Missing outcome data	Low
Risk of bias in measurement of the outcome	Low
Risk of bias in selection of the reported result	Some concerns<br>Direction of bias: Unpredictable
Overall risk of bias	Some concerns<br>Direction of bias: Unpredictable

10. In patients with proven HSV encephalitis (positive CSF PCR or intrathecal antibody production), does administering aciclovir for more than two weeks reduce morbidity compared to two-week aciclovir therapy?

10a. When should aciclovir resistance be suspected and what action should be taken?

Evidence summary

PICO 10:
Of 702 articles screened, one was included for this PICO (**Figure S10.1**) (49). Patients with HSV encephalitis (n=55) were recruited to a prospective study of infectious encephalitis in France and their clinical profiles along with the management of aciclovir therapy was reported (**Table S10.1** for characteristics of the study). Evidence from this observational study suggests longer durations of aciclovir (beyond 14 days) are not associated with improved patient outcomes. In terms of bias, the intervention was not blinded or randomised, and the patients may well have received a longer duration of aciclovir if they had more severe disease. Also, some centres gave a longer duration of treatment than other centres, which could lead to confounding. This is an observational cohort study and conclusions regarding the effect of duration of treatment are limited (**Table S10.2**).

No randomised trials have been conducted to compare durations of aciclovir therapy for HSV encephalitis. As in other guidelines, this good practice statement has been made due to limited and indirect non-randomised evidence (15,16,50,51). In the original randomised trials of aciclovir for HSV encephalitis, duration of treatment was 10 days (30,52). There have, however, been reports of clinical relapse after 10 days treatment (53,54). Although an ongoing immune-mediated and inflammatory reaction to the infection is thought to be the major pathogenic process of relapse, there is evidence for continuing viral replication in some cases (55).

PICO 10a:
No paper met the inclusion criteria for the PICO (**Figure S10.1**). Aciclovir resistance in encephalitis is described very rarely in the literature. Among non-neurological isolates from immunocompetent patients who are not taking long-term suppressive aciclovir therapy, resistance is also very rare (56–58). Resistance, evidence for which comes mostly from non-encephalitis patients, is mostly described in individuals who are immunosuppressed and/or taking long-term suppressive or preventative aciclovir therapy (59).

Search string

(("infectious encephalitis"[MeSH Terms] OR "encephalit*"[Title/Abstract]) AND ("acyclovir"[Title/Abstract] OR "acyclovir"[MeSH Terms])) AND (2008:2021[pdat])

Flowchart

Figure S10.1. Flow chart for study inclusion for PICO 10 and 10a

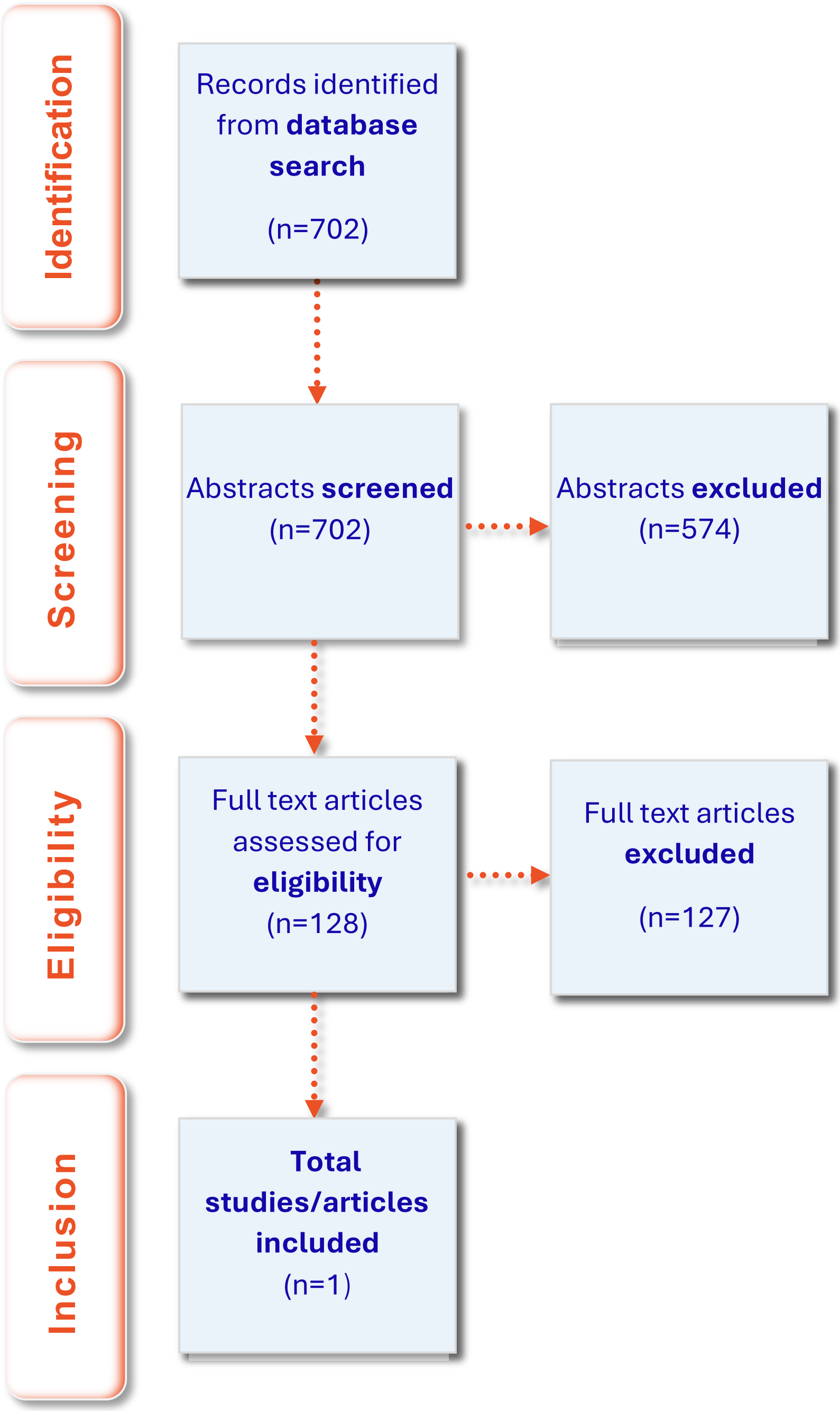

Characteristics of included studies

Table S10.1. Characteristics of included studies for PICO 10

First author	Study period	Country	Study design	Recruitment method	Inclusion criteria	Number of participants	Outcomes
Stahl	2007	France	Cohort	Prospective	Patients with HSE	55	Outcome was not significantly associated with dosage or duration of aciclovir treatment with or without adjustment for confounders.<br><br>Duration of aciclovir was not a significant predictor of outcome (dichotomised as poor and favourable) on univariate (p=0.42) or multivariate analysis (OR 1.01, 95%CI 0.86-1.20, p= 0.88)

HSE: Herpes simplex encephalitis; OR: Odds ratio

Quality assessment

Table S10.2. Quality assessment of study using ROBINS-I assessment tool

Bias	Detail
Bias due to confounding	*Aciclovir dose not controlled for in multivariate analysis*
Bias in selection of participants into the study	*Low*
Bias in classification of interventions	*Severity of illness will have affected judgement of length of treatment*
Bias due to deviations from intended interventions	*Unknown whether aciclovir dose was reasonably split between those with different aciclovir durations*
Bias due to missing data	*Low*
Bias in measurement of outcomes	*Low*
Overall	*Main issue is that the intervention was not blinded or randomised, and the patient may well have received a longer duration of aciclovir if they had more severe disease. Secondly, it may be that there was a practice of giving a longer duration of treatment in some centres, and this could lead to confounding. This is an observational cohort study and conclusions regarding the effect of duration of treatment are limited.*

11. In patients with proven HSV encephalitis, does the addition of corticosteroid therapy improve outcome compared to standard care alone?

11a. If yes, when should steroids be introduced?

Evidence summary

Three reports were eligible for inclusion (**Figure S11.1** and **Table S11.1**) (60–62). The review by Ramos-Estebanes et al. included case reports and case series on the use of corticosteroids in human HSV encephalitis (61). The results support the use of corticosteroids, either isolated or combined with acyclovir, however, the quality assessment indicated a high risk of bias (**Table S11.3**). The German trial on Aciclovir and Corticosteroids in Herpes-simplex-virus Encephalitis (GACHE) trial randomized 21 adult patients with HSV encephalitis to receive dexamethasone in addition to aciclovir and 20 to receive placebo (60). There was no difference in modified Rankin scale, mortality, functional outcome (Glasgow outcome score), quality of life (EQ-5D), and neuropsychological tests between groups. The study was terminated prematurely due to slow recruitment thus the small sample size does not enable firm conclusions. The recent UK DexEnceph trial randomised 47 patients to adjunctive dexamethasone and 47 to placebo (62). Again, there was no difference in primary outcome (i.e., verbal memory score as determined by the Wechsler Memory Scale [WMS-IV] Auditory Memory Index at 26 weeks) or secondary outcomes (i.e., other neuropsychology, imaging, and functional outcomes) between groups.

Search string

(((("infectious encephalitis"[MeSH Terms] OR "encephalit*"[Title/Abstract]) AND ("hsv"[Title/Abstract] OR "herpes"[Title/Abstract] OR "herpes simplex"[MeSH Terms] OR "herpesvirus 1, human"[MeSH Terms])) OR "encephalitis, herpes simplex"[MeSH Terms]) AND ("hydrocortisone"[MeSH Terms] OR "prednisolone"[MeSH Terms] OR "methylprednisolone"[MeSH Terms] OR "dexamethasone"[MeSH Terms] OR "glucocorticoids"[MeSH Terms] OR "steroids/drug therapy"[MeSH Terms] OR "corticosteriod"[Title/Abstract] OR "hydrocortisone"[Title/Abstract] OR "prednisolone"[Title/Abstract] OR "methylprednisolone"[Title/Abstract] OR "dexamethasone"[Title/Abstract] OR "glucocorticoid"[Title/Abstract] OR "steroid"[Title/Abstract])) AND (2008:2021[pdat])

Flowchart

Figure S11.1. Flow chart for study inclusion for PICO 11 and 11a

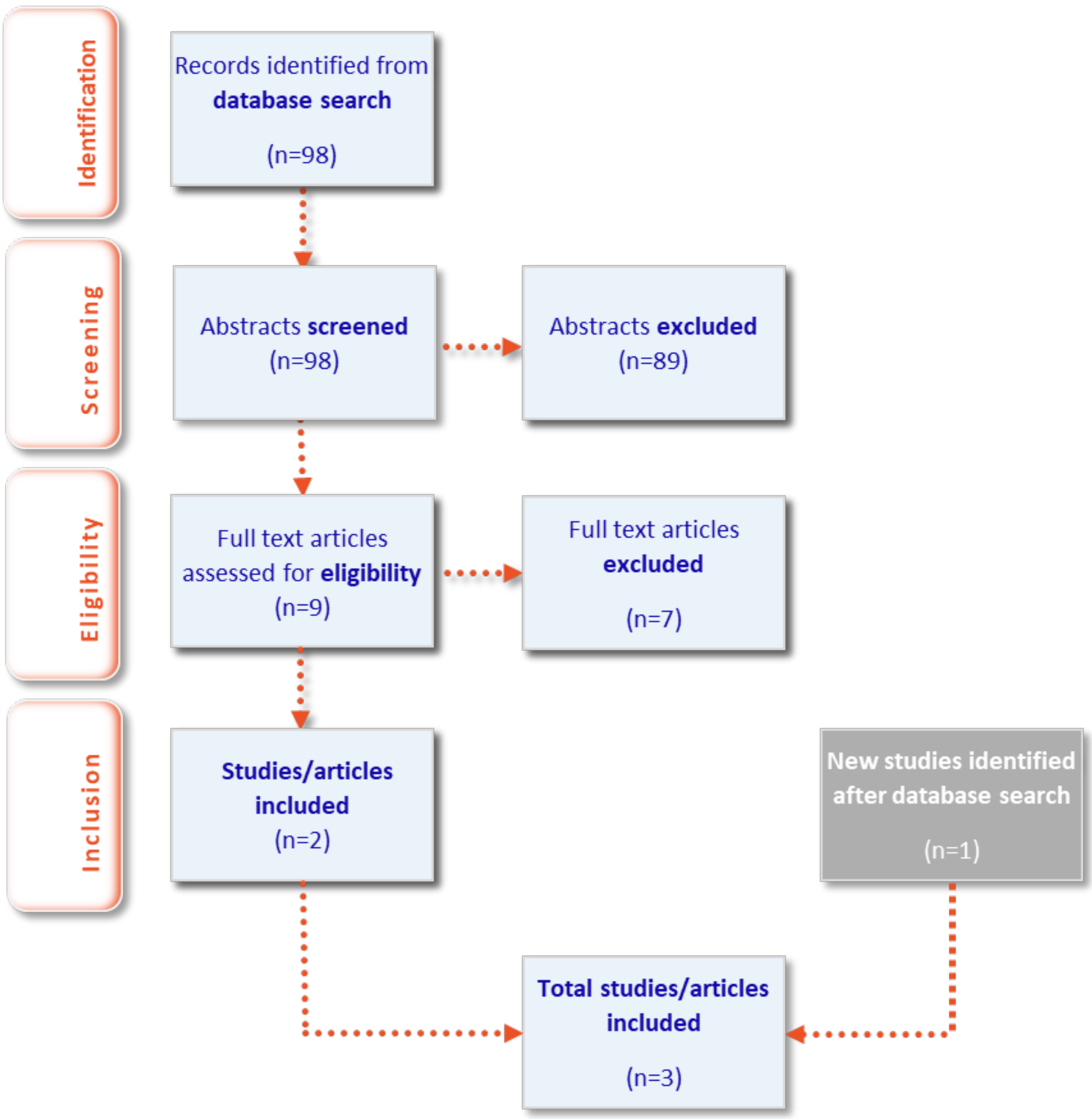

Characteristics of included studies

Table S11.1. Characteristics of included studies for PICO 11

First author	Study period	Country	Study design	Recruitment method	Inclusion criteria	Number of participants	Outcomes
Meyding-Lamade[1]	Nov 2007-Dec 2012	Germany	RCT	Prospective	Aged 18-85 years, lab-proven HSVE, focal neurological signs for ≤5 days prior to admission, negative pregnancy test for women of childbearing potential	19 corticosteroid therapy; 19 no corticosteroid therapy	No difference was observed in the primary endpoint (i.e., binary functional outcome after 6 months assessed using mRS [0–2 vs. 3–6]) between groups.<br><br>There were no significant differences in the secondary endpoints (GOS, mRS, quality of life, neuropsychological testing).
Ramos-Estebanes	1970- Mar 2013	Multi-country	Systematic review	Retrospective	Original articles published between 1970 and Mar 2013	55 patients from 5 case reports and 2 case series	In HSVE, animal models and human reports support the use of corticosteroids, either isolated or combined with acyclovir.
Solomon	Sep 2016-Feb 2022	UK	RCT	Prospective	Aged ≥16 years, met criteria for suspected encephalitis, positive CSF PCR for HSV DNA reported no longer than seven days prior to randomisation and receiving intravenous aciclovir 10mg/kg three times daily, or reduced dose if clinically indicated	47 corticosteroid therapy; 47 no corticosteroid therapy	No difference was observed in the primary endpoint (i.e., verbal memory score at 26 weeks; 1.8, 95% CI: -9.6-13.1, p=0.76) between groups.<br><br>There were no significant differences in the secondary endpoints (other neuropsychology, imaging and functional outcomes).

[1]The initially planned sample size was 372 patients. The trial was stopped prematurely due to slow recruitment after 41 patients had been randomized, therefore the study is massively underpowered.

CSF: Cerebrospinal fluid; GOS: Glasgow outcome scale; HSVE: Herpes simplex virus encephalitis; mRS: Modified Rankin scale; PCR: Polymerase chain reaction; RCT: Randomised controlled trial; UK: United Kingdom

Summary of findings tables

Table S11.2. Corticosteroid therapy compared to no corticosteroid therapy for adults with proven HSV encephalitis

Certainty assessment							Summary of findings				
							Study event rates (%)			Anticipated absolute effects	
Participants (studies) Follow-up	Risk of bias	Inconsistency	Indirectness	Imprecision	Publication bias	Overall certainty of evidence	With no corticosteroid therapy	With corticosteroid therapy	Relative effect (95% CI)	Risk with no corticosteroid therapy	Risk difference with corticosteroid therapy
**Memory score - Wechsler (follow-up: 26 weeks)** [DexEnceph trial 2025]											
81 (1 RCT)	not serious	not serious	serious[a]	not serious	none	⊕⊕⊕◯ Moderate[a]	42	39	-	42	mean **1.77 higher** (9.57 lower to 13.12 higher)
**Favourable functional outcome (follow-up: 6 months; assessed with: Modified Rankin scale score 0-2) [GACHE trial 2019]**											
38 (1 RCT)	not serious	not serious	not serious	serious[b]	none	⊕⊕⊕◯ Moderate[b]	12/19 (63.2%)	12/19 (63.2%)	**RR 1.00** (0.62 to 1.63)	12/19 (63.2%)	**0 fewer per 1,000** (from 240 fewer to 398 more)

CI: Confidence interval; RR: Risk ratio

Explanations

a. The average time of 7 days from presentation to receipt of corticosteroid therapy is much longer than the ideal timing of the intervention.

b. The target sample size was not met.

Quality assessment

Table S11.3. Methodological quality of Ramos-Estebanes et al systematic review using AMSTAR 2 assessment tool

AMSTAR 2 items	Yes	Partial yes	No
1. Did the research questions and inclusion criteria for the review include the components of PICO	√		
2. Did the report of the review contain an explicit statement that the review methods were established prior to the conduct of the review and did the report justify any significant deviations from the protocol?			√
3. Did the review authors explain their selection of the study designs for inclusion in the review?			√
4. Did the review authors use a comprehensive literature search strategy?[1]			√
5. Did the review authors perform study selection in duplicate?			√
6. Did the review authors perform data extraction in duplicate?			√
7. Did the review authors provide a list of excluded studies and justify the exclusions?[1]			√
8. Did the review authors describe the included studies in adequate detail?		√	
9. Did the review authors use a satisfactory technique for assessing the RoB in individual studies that were included in the review?[1]			√
10. Did the review authors report on the sources of funding for the studies included in the review?			√
11. If meta-analysis was performed did the review authors use appropriate methods for statistical combination of results?		NA	
12. If meta-analysis was performed, did the review authors assess the potential impact of RoB in individual studies on the results of the meta-analysis or other evidence synthesis?		NA	
13. Did the review authors account for RoB in individual studies when interpreting/discussing the results of the review?*			√
14. Did the review authors provide a satisfactory explanation for, and discussion of, any heterogeneity observed in the results of the review?			√
15. If they performed quantitative synthesis did the review authors carry out an adequate investigation of publication bias (small study bias) and discuss its likely impact on the results of the review?[1]		NA	
16. Did the review authors report any potential sources of conflict of interest, including any funding they received for conducting the review?			√

[1]Critical domain items; NA: Not applicable; RoB: Risk of bias

Table S11.4. Risk of bias assessment of Meyding-Lamade et al. and Solomon et al. study using ROB 2 assessment tool

Study	RoB arising from randomisation process	RoB due to deviations from the intended interventions	Missing outcome data	RoB in measurement of the outcome	RoB in selection of the reported result	Overall RoB
Meyding-Lamade et al.	Low	Low	Low	Low	Low	Low
Solomon et al.	Low	Low	Low	Low	Low	Low

RoB: Risk of bias

12. In patients with proven VZV encephalitis (positive CSF PCR or intrathecal antibody production), does treatment with high dose aciclovir (usually 20 mg/kg three times per day) compared to 10 mg/kg aciclovir three times per day reduce mortality and morbidity?

Evidence summary

No comparative studies investigating the use of 20 mg/kg per dose of aciclovir was identified (**Figure S12.1**). Only one non-randomised study performed a head-to-head comparison of outcomes with 10mg/kg versus 15 mg/kg dosing, which showed no difference in efficacy (63). The WG noted mixed practice with regards to aciclovir dosing for VZV; however, 20 mg/kg is not commonly used. A dose of 15 mg/kg is reportedly in use by some clinicians, based on the fact that aciclovir demonstrates lower in vitro activity against VZV compared to HSV (64). Aciclovir has several potential adverse effects, including renal failure, which may be more common with higher doses (65,66).

The commonality of reported vasculitis in encephalitis varies. A nationwide Danish prospective cohort study showed the occurrence of cerebral vasculitis in 16% (14/92) of adults with VZV encephalitis (67), while a literature review of 34 cases of cerebrovascular complications after adult-onset VZV encephalitis reported 11 cases (32%) with evidence of vasculitis (68). Early studies show a benefit of steroids in VZV encephalitis prior to the use of aciclovir.

Search string

((((infectious encephalitis[MeSH Terms]) OR (encephalit*[Title/Abstract])) OR (cerebellitis[Title/Abstract])) AND ((((varicella[Title/Abstract]) OR (zoster[Title/Abstract])) OR (vzv[Title/Abstract])) OR (encephalitis, varicella zoster[MeSH Terms]))) AND ((acyclovir[MeSH Terms]) OR (acyclovir[Title/Abstract]))

Flowchart

Figure S12.1. Flow chart for study inclusion for PICO 12

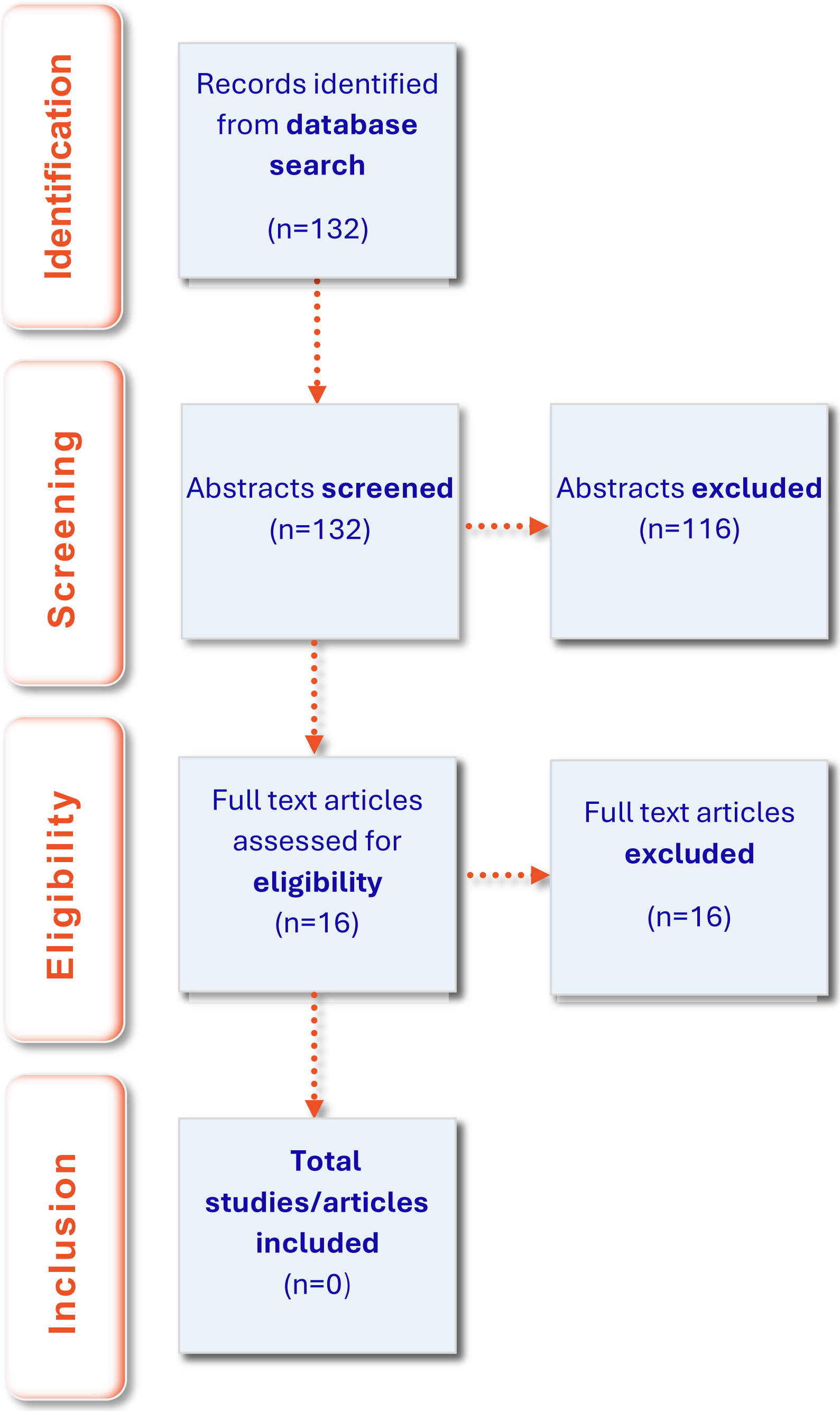

13. What is the impact on morbidity and mortality of prophylactic antiseizure treatment versus no prophylactic antiseizure treatment in cases of infectious encephalitis?

Evidence summary

Of 382 screened, no paper met the PICO inclusion criteria (**Figure S13.1**). Thus, no evidence was identified for the benefit of primary prophylaxis with antiseizure medication in patients with infectious encephalitis; however, there are known potential harms (69). Indirect evidence was provided by two studies. A cohort study to investigate the long-term seizure outcome in patients with convulsive status epilepticus due to encephalitis showed that the number of antiseizure drugs was not significantly associated with terminal seizure freedom after the acute phase (70). A further cohort study showed that the risk of seizure relapse did not differ between patients on antiseizure drugs and those without (71).

Acute management is very important as seizures in encephalitis are associated with increased morbidity and mortality (6,72). Non-convulsive status epilepticus must be considered as a cause of ongoing abnormal mental status in the absence of overt motor seizure activity, for which EEG may be required to confirm a diagnosis.

While studies specific to encephalitis do not demonstrate worse longer-term outcomes from not continuing antiseizure medication after the initial phase, studies in epilepsy show that multiple seizures and structural abnormalities can increase risk of harm if not treated. This is consistent with the increased seizure relapse in patients with multiple seizures and structural abnormalities reported by Peng et al. (71). Seizures are more likely if MRI brain abnormalities persist beyond the initial scan; however, studies are lacking. A secondary analysis of an observational study in encephalitis patients suggested an increased volume of cerebral oedema is associated with risk of acute seizure activity and adverse neurological outcomes (73).

Epileptic seizures are common, in the acute stage as well as later on in encephalitis (74,75). A Swedish national register study has shown that one fifth of HSV encephalitis patients are readmitted to hospital because of epileptic seizures, often late after the diagnosis of HSV encephalitis (median 9 months) (76). The duration of antiseizure treatment is unknown (77).

Search string

(("infectious encephalitis"[MeSH Terms] OR "encephalit*"[Title/Abstract]) AND ("valproic acid"[Title/Abstract] OR "clonazepam"[Title/Abstract] OR "zonisamide"[Title/Abstract] OR "topiramate"[Title/Abstract] OR "lamotrigine"[Title/Abstract] OR "vigabatrin"[Title/Abstract]

OR "lacosamide"[Title/Abstract] OR "pregabalin"[Title/Abstract] OR "gabapentin"[Title/Abstract] OR "oxcarbazepine"[Title/Abstract] OR "carbamazepine"[Title/Abstract] OR "phenobarbital"[Title/Abstract] OR "phenytoin"[Title/Abstract] OR "anticonvulsants"[Title/Abstract] OR "antiseizure therapy"[Title/Abstract] OR "levetiracetam"[Title/Abstract] OR "rufinamide"[Title/Abstract] OR "topiramate"[MeSH Terms] OR "anticonvulsants"[MeSH Terms] OR "levetiracetam"[MeSH Terms] OR "phenytoin"[MeSH Terms] OR "phenobarbital"[MeSH Terms] OR "carbamazepine"[MeSH Terms] OR "oxcarbazepine"[MeSH Terms] OR "gabapentin"[MeSH Terms] OR "pregabalin"[MeSH Terms] OR "lacosamide"[MeSH Terms] OR "vigabatrin"[MeSH Terms] OR "lamotrigine"[MeSH Terms] OR "zonisamide"[MeSH Terms] OR "clonazepam"[MeSH Terms] OR "valproic acid"[MeSH Terms])) AND (2008:2021[pdat])

Flowchart

Figure S13.1. Flow chart for study inclusion for PICO 13

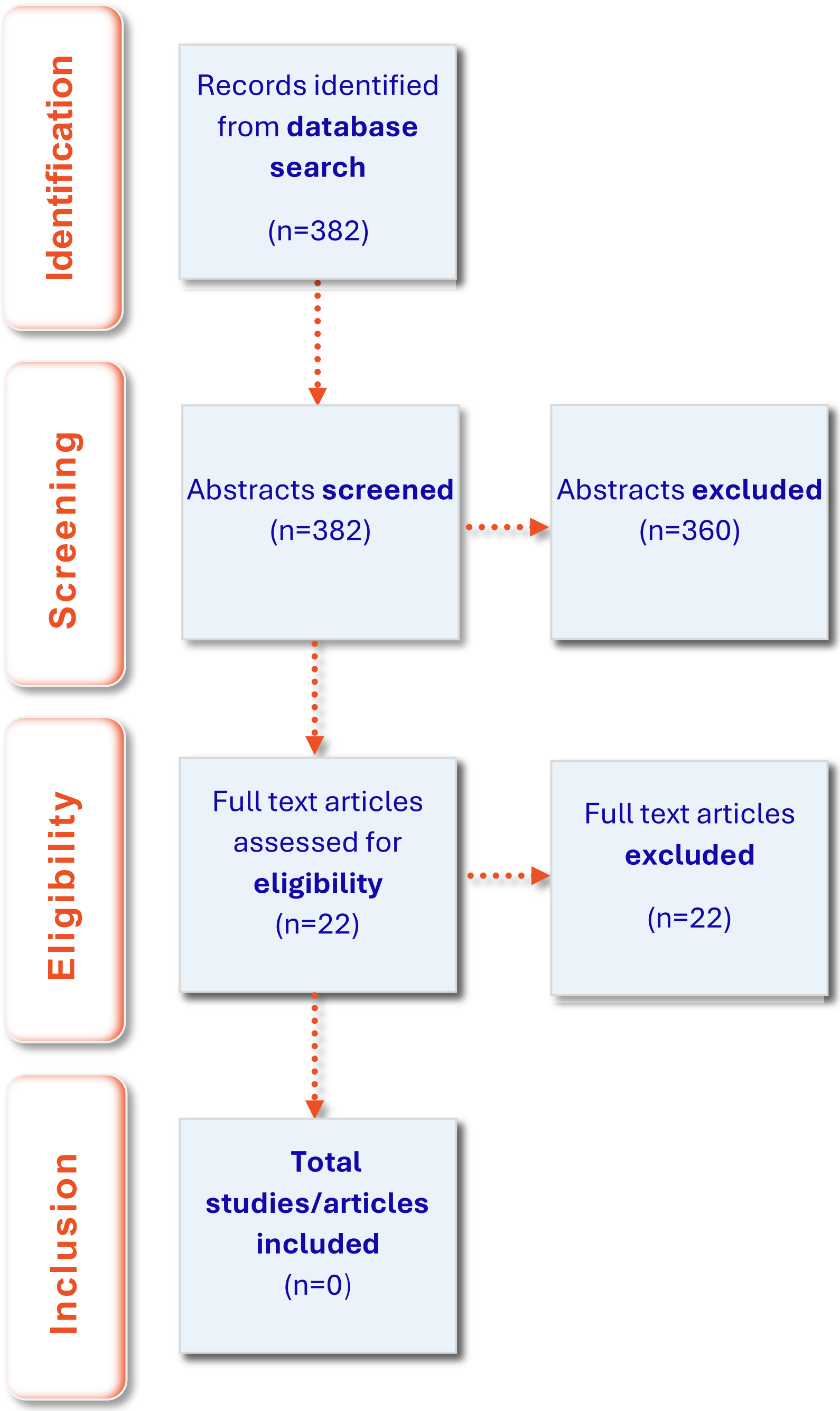

14. In patients with infectious encephalitis and/or deep worsening coma score or status epilepticus, does admission to intensive care improve outcome and reduce mortality compared to no admission to intensive care?

Evidence summary

Of 209 screened, only one relevant study was identified for this PICO (see **Figure S14.1** for flow diagram, and **Tables S14.1** for characteristics and **S14.2** for quality assessment of the study) (78). Singh et al. conducted a retrospective review of consecutive adult patients with HSV encephalitis admitted to the Mayo Clinic and showed that ICU admission was associated with an unfavourable outcome at discharge in univariate but not multivariate analysis. The factors associated with poor outcome on multivariate analysis were older age, coma, restricted diffusion on MRI brain scan, and aciclovir started after the first day of admission. The need for ICU in a setting where it is freely available was likely reflective of how sick the patients were, in this non-randomised retrospective study, with no matching for other risk factors for poor outcome. When other poor prognostic indicators were included in the multivariable model, the apparent effect of ICU admission was no longer significant.

There may not be many studies in encephalitis, but relevant evidence exists from other causes of coma and other neurological conditions (e.g., bacterial meningitis, stroke) which supports the use of critical care. Chalfin et al. reported a higher mortality in hospital if the transfer of patients with critical illness to ICU was delayed; when patients with intracerebral haemorrhage and other neurological diseases were combined, this became the largest subgroup within the overall cohort (79). Rincon et al. described a similar association between delay in transfer to a neuro-ICU and poor outcome in critically ill patients with stroke (80). Helbok et al. reviewed the literature on intracranial pressure (ICP) monitoring in conditions other than traumatic brain injury (TBI) and provided recommendations on how the technique may be used in patient management (81). Increased ICP can adversely affect outcome, particularly when it fails to respond to treatment. However, patients with elevated ICP can still have favourable outcomes. Although the influence of ICP-based care on outcome in non-TBI conditions appears less robust than in TBI, monitoring ICP and cerebral perfusion pressure can play a role in guiding therapy in selected patients.

Search string

(("encephalit*"[Title/Abstract] OR "infectious encephalitis"[MeSH Terms]) AND ("critical care"[MeSH Terms] OR "critical care"[MeSH Terms] OR "critical care"[MeSH Terms] OR

"intensive therapy unit"[Title/Abstract] OR "icu"[Title/Abstract] OR "itu"[Title/Abstract]))
AND (2008:2021[pdat])

Flowchart

Figure S14.1. Flow chart for study inclusion for PICO 14

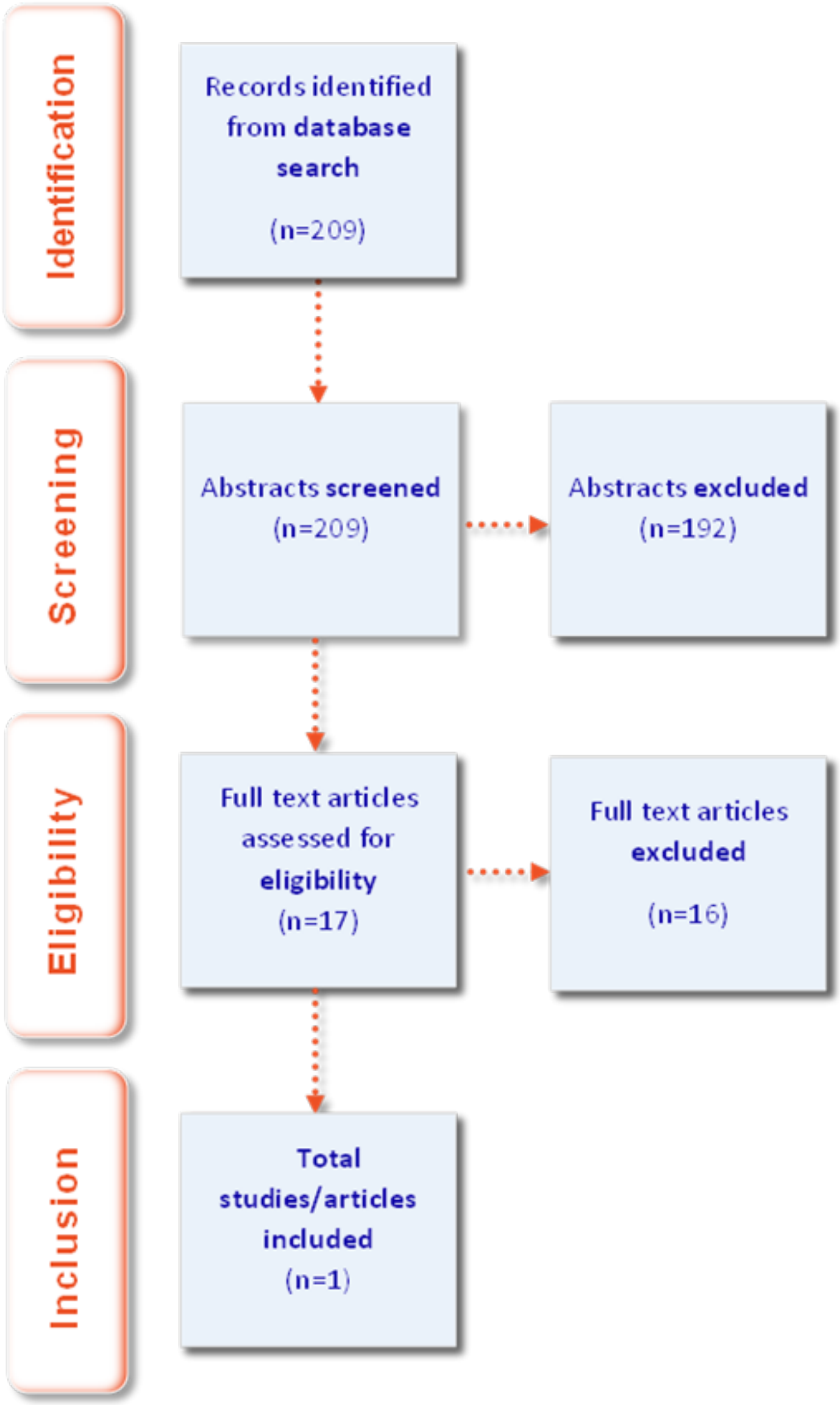

Characteristics of included studies

Table S14.1. Characteristics of included studies for PICO 14

First author	Study period	Country	Study design	Recruitment method	Inclusion criteria	Number of participants	Outcomes (admission to ICU vs no admission to ICU)
Singh	1995-2013	USA	Retrospective review of consecutive patients (database)	Electronic data search system	(1) Satisfied criteria for HSE encephalitis, (2) were admitted and treated at Mayo Clinic Hospital, and (3) were 18 years or older	45 PCR-positive HSE cases	ICU admission was associated with poor outcome (mRS 3-6) at discharge in the univariate (Table x) but not the multivariate analysis. On multivariate analyses, older age, coma, restricted diffusion on MRI brain scan, and acyclovir started after the first day of admission were associated with poor outcome.

HSE: Herpes simplex encephalitis; ICU: Intensive care unit; MRI: Magnetic resonance imaging; mRS: Modified Ranskin Score; PCR: Polymerase chain reaction; USA: United States of America

Quality assessment

Table S14.2. Quality assessment of Singh et al. using Newcastle Ottawa scale for cross-sectional studies

	Total score	Selection				Comparability	Outcome	
		Representativeness of sample	Sample size	Non-respondents	Ascertainment of exposure	Based on design and analysis	Assessment of outcome	Statistical test
Singh et al., 2016	7				**	**	**	*

15. In a patient with HSE with relapse, does autoantibody testing improve the chance of identifying the cause of relapse compared to not testing for autoantibodies

Evidence summary

Of the 223 studies identified by the search, no study met the inclusion criteria (**Figure S15.1**). Three background studies however on autoantibody testing in HSV encephalitis provided relevant information to help develop a good practice statement. Armangue et al. included 14 patients (8 adults) with non-viral relapsing symptoms post-HSV encephalitis in a prospective cohort study in Spain (82). All patients were examined for autoantibodies, and five patients had IgG antibodies against glutamate ionotropic receptor N-methyl-D-aspartate (NMDA) type subunit 1 (GluN1) and three patients had antibodies against unknown neuronal antigens. All eight patients with HSV encephalitis had recurrent symptoms secondary to auto-immune causes. A larger prospective observational study conducted by Armangue et al. in 19 Spanish centres demonstrated that autoimmune encephalitis occurs in 27% of patients with HSV encephalitis (83). Nosadini et al. conducted a systematic literature review on 43 patients (12 adults) with biphasic disease with HSV encephalitis followed by anti-NMDAR encephalitis (84). All patients were tested for autoantibodies and all 12 were negative for HSV DNA by PCR of CSF, and positive for anti-NMDAR antibodies in either serum or CSF.

Search string

(((("infectious encephalitis"[MeSH Terms] OR "encephalit*"[Title/Abstract]) AND ("hsv"[Title/Abstract] OR "herpes"[Title/Abstract] OR "herpesvirus 1, human"[MeSH Terms] OR "herpes simplex"[MeSH Terms])) OR "encephalitis, herpes simplex"[MeSH Terms]) AND ("recurrence"[MeSH Terms] OR "relaps*"[Title/Abstract] OR "recurr*"[Title/Abstract] OR "recurrence"[MeSH Terms])) AND (2008:2021[pdat])

Flowchart

Figure S15.1. Flow chart for study inclusion for PICO 15

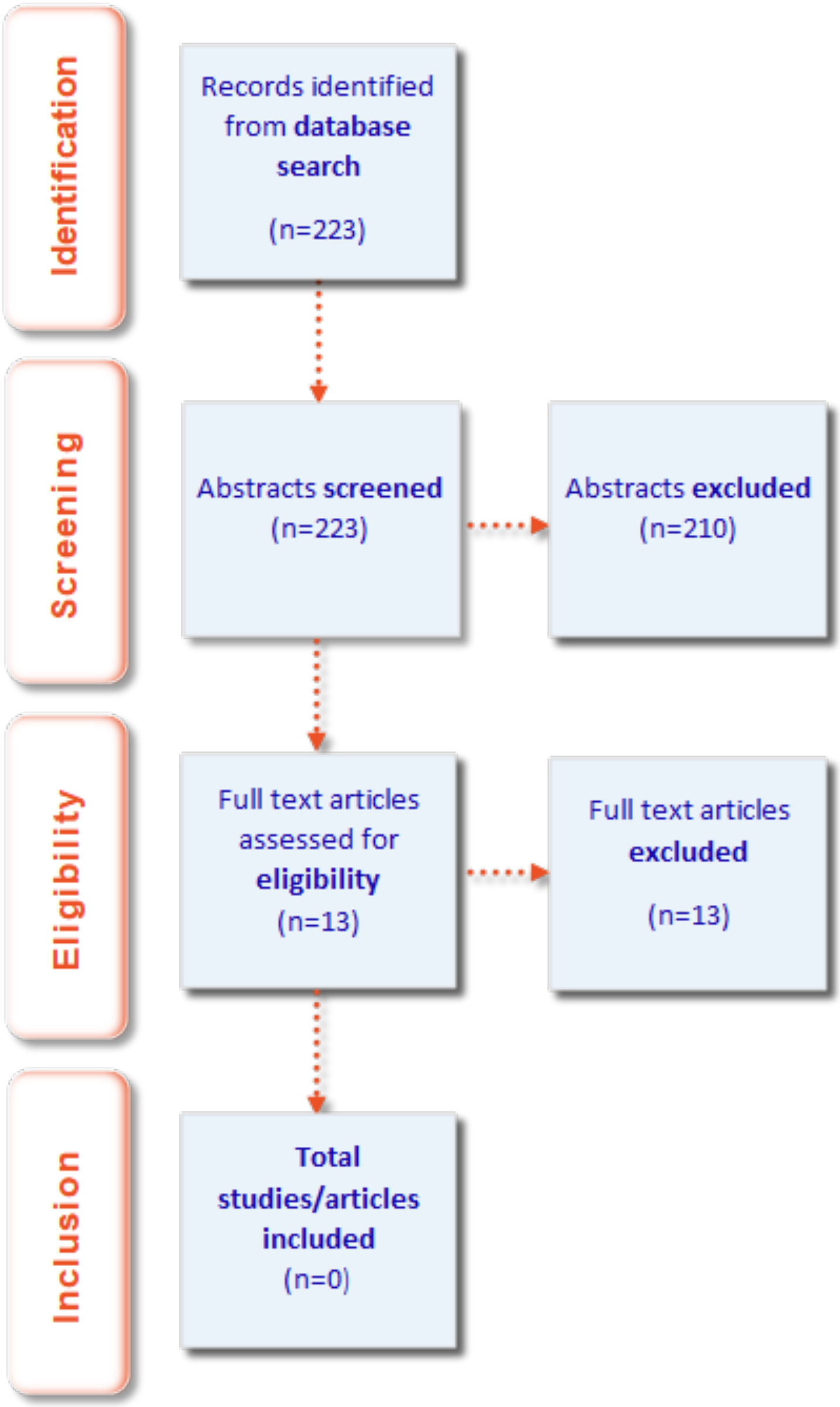

16. In patients with infectious encephalitis, does follow-up care and rehabilitation reduce morbidity compared to no follow-up care and rehabilitation?

Evidence summary

Of 1,727 screened, two papers met the inclusion criteria for this PICO (**see Figure S16.1** for flow diagram, and **Tables S16.1** for characteristics and **S16.2** for quality assessment of the studies). Christie et al. conducted a systematic review on outcomes following rehabilitative interventions among children and adults with infectious encephalitis (85). Of the 20 studies (37 participants in total), 19 included adult patients. Most studies evaluated the effectiveness of interventions targeting sequelae of HSV encephalitis, in 15 out of 37 adults. A range of neuropsychological assessments were used to measure the severity of functional impairment at baseline, including level of cognitive ability or severity of amnesia due to encephalitis and the follow-up assessment. Ten studies did not provide information on the assessment tool used to evaluate the severity of deficits experienced by the patient at baseline. All studies reported a beneficial effect of rehabilitation interventions in a variety of settings among paediatric and adult patients with infectious encephalitis, with several studies highlighting incomplete recovery and persistence of residual impairments on completion of rehabilitation intervention. Cooper et al. conducted 30 narrative interviews with 45 people affected by HSV encephalitis and their caregivers about their post-discharge experience (86). For the small number of people in the study [6/29 (21%)] who received structured rehabilitation, their narratives often reported a different story of 'recovery' in two ways: firstly, rehabilitation was highlighted as important for aiding peoples' ability to understand the problems they experience after leaving hospital. Being given explanations for why they struggle with routine tasks, like washing, meant that people were better able to make sense of their situation. This contrasted with the experience of others, who usually returned home unable to understand the nature of their problems. Secondly, the care which was received during rehabilitation, such as help to re-learn activities like cooking, enabled people to re-orient themselves to their everyday lives upon their return home. Focused rehabilitation therefore facilitated a more supported transition between the hospital and the home.

Search string

(("infectious encephalitis"[MeSH Terms] OR "encephalit*"[Title/Abstract]) AND ("rehabilitation"[MeSH Terms] OR "rehab*"[Title/Abstract] OR "follow-up"[Title/Abstract] OR "long-term"[Title/Abstract] OR "post-acute"[Title/Abstract] OR "postacute"[Title/Abstract] OR "management"[Title/Abstract] OR "length of stay"[Title/Abstract] OR "recovery of function"[Title/Abstract] OR "functional outcome"[Title/Abstract] OR "functional independence measure"[Title/Abstract] OR "functional recovery"[Title/Abstract] OR

"discharge destination"[Title/Abstract] OR "discharge status"[Title/Abstract] OR "neuropsychology"[MeSH Terms])) AND (2008:2021[pdat])

For searches with >1,000 results, a hierarchical approach was used:

a) Limit the search to systematic reviews. If a systematic review was found that answered the question comprehensively, the primary articles search started from the date of that review's search.
b) If no relevant systematic review was found, all primary studies were screened. For searches with >1,000 results, this was conducted by one reviewer initially, with 20% quality control checked by another reviewer. After the first 1,000 results, we stopped to recalibrate if >10% of decisions were discrepant.

Flowchart

Figure S16.1. Flow chart for study inclusion for PICO 16

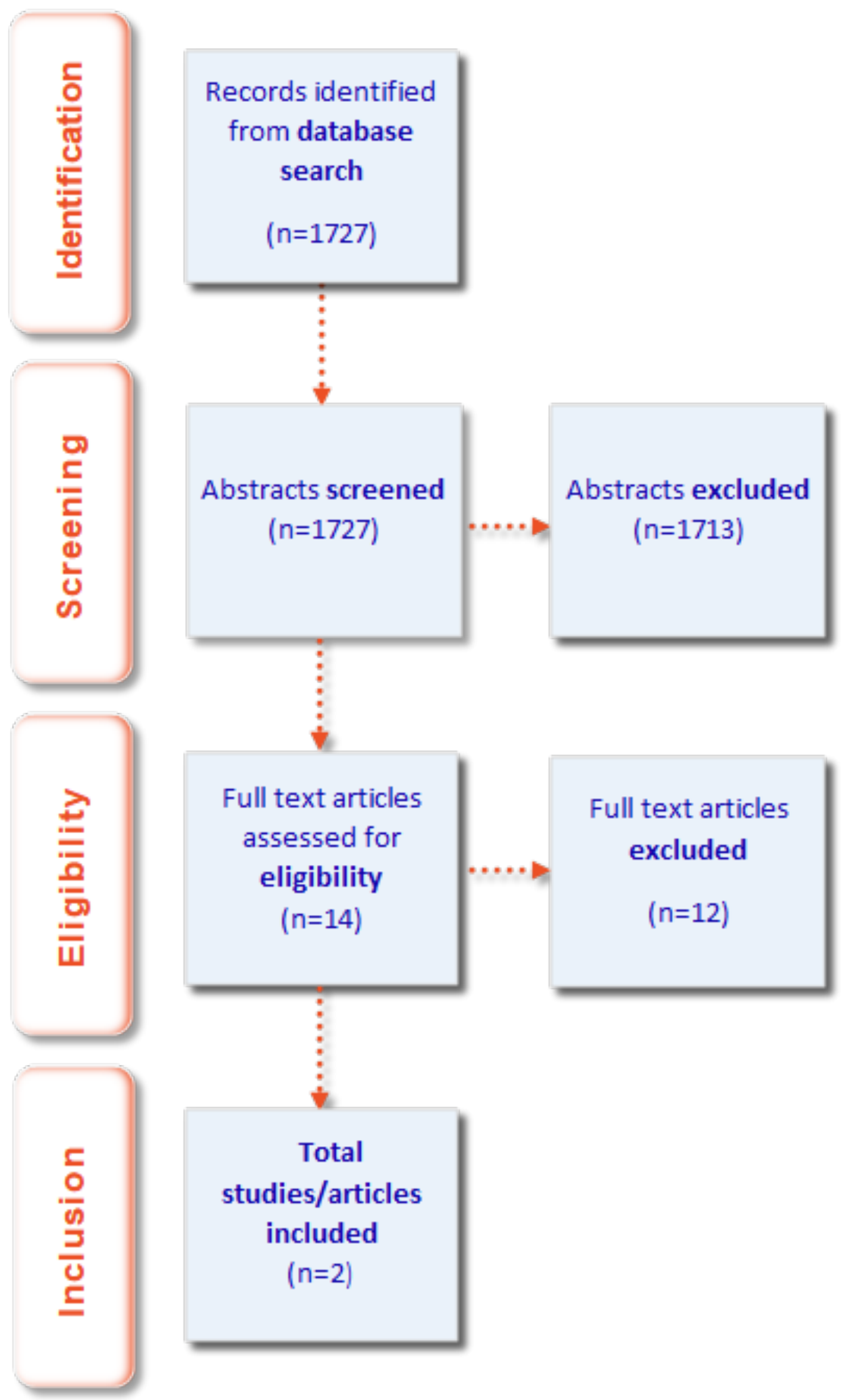

Characteristics of included studies

Table S16.1. Characteristics of included studies for PICO 16

First author	Study period	Country	Study design	Recruitment method	Inclusion criteria	Number of participants	Outcomes
Christie	Up to December 2017	Global	Systematic review		All studies that reported outcome data of rehabilitation interventions delivered to patients with a primary diagnosis of infectious encephalitis in an inpatient, outpatient or community rehabilitation setting were considered eligible in this review.	19 studies included adult patients (37 participants in total). A variety of agents produced encephalitis, with the majority of studies looking at the effectiveness on interventions targeting sequelae of HSV encephalitis in 15 out of 37 adults.	Task success (%) of messages and target behaviours<br><br>Clinical evaluation, FIM, MMSE, CCT and MEAMS<br><br>Nine-hole peg test, all ADLs, homemaking, ambulation, transfers, bowel and bladder, safety, behaviour, speech, comprehension, reading, math and writing.<br><br>FIM score at admission and discharge.<br><br>WAIS–Revised, AVLT
Cooper			Narrative interviews	Retrospective and prospective	People affected by HSV encephalitis and their significant others	6/29 (21%) received structured rehabilitation	For the small number of people in the study [6/29 (21%)] who received structured rehabilitation, their narratives tell a story of 'recovery' in two ways: i) rehabilitation is highlighted as important for aiding peoples' ability to understand the problems they experience after leaving hospital.; ii) The care which is received during rehabilitation, such as having help to re-learn activities like cooking, enables people to re-orient themselves to their everyday lives upon their return home.

Quality assessment

Table S16.2. Quality assessment of Christie et al. study using AMSTAR 2 assessment tool

AMSTAR 2 items	Yes	Partial yes	No
1. Did the research questions and inclusion criteria for the review include the components of PICO	√		
2. Did the report of the review contain an explicit statement that the review methods were established prior to the conduct of the review and did the report justify any significant deviations from the protocol?			√
3. Did the review authors explain their selection of the study designs for inclusion in the review?	√		
4. Did the review authors use a comprehensive literature search strategy?[1]	√		
5. Did the review authors perform study selection in duplicate?	√		
6. Did the review authors perform data extraction in duplicate?	√		
7. Did the review authors provide a list of excluded studies and justify the exclusions?[1]			√
8. Did the review authors describe the included studies in adequate detail?		√	
9. Did the review authors use a satisfactory technique for assessing the RoB in individual studies that were included in the review?[1]	√		
10. Did the review authors report on the sources of funding for the studies included in the review?			√
11. If meta-analysis was performed did the review authors use appropriate methods for statistical combination of results?		NA	
12. If meta-analysis was performed, did the review authors assess the potential impact of RoB in individual studies on the results of the meta-analysis or other evidence synthesis?		NA	
13. Did the review authors account for RoB in individual studies when interpreting/discussing the results of the review?[1]	√		
14. Did the review authors provide a satisfactory explanation for, and discussion of, any heterogeneity observed in the results of the review?	√		
15. If they performed quantitative synthesis did the review authors carry out an adequate investigation of publication bias (small study bias) and discuss its likely impact on the results of the review?[1]		NA	
16. Did the review authors report any potential sources of conflict of interest, including any funding they received for conducting the review?	√		

[1]Critical domain items; NA: Not applicable; RoB = Risk of bias

Table S16.3. Quality assessment of Cooper at al. using JBI critical appraisal checklist for qualitative research

Question	Response
Is there congruity between the stated philosophical perspective and the research methodology?	Yes
Is there congruity between the research methodology and the research question or objectives?	Yes
Is there congruity between the research methodology and the methods used to collect data?	Yes
Is there congruity between the research methodology and the representation and analysis of data?	No
Is there congruity between the research methodology and the interpretation of results?	Yes
Is there a statement locating the researcher culturally or theoretically?	No
Is the influence of the researcher on the research, and vice- versa, addressed?	No
Are participants, and their voices, adequately represented?	No
Is the research ethical according to current criteria or, for recent studies, and is there evidence of ethical approval by an appropriate body?	Yes
Do the conclusions drawn in the research report flow from the analysis, or interpretation, of the data?	Yes

References

1. Basaran S, Yavuz SS, Bali EA, Cagatay A, Oncul O, Ozsut H, et al. Hyponatremia Is Predictive of HSV-1 Encephalitis among Patients with Viral Encephalitis. Tohoku J Exp Med. 2019 Mar;247(3):189–95.

2. Jordan B, Kösling S, Emmer A, Koch A, Müller T, Kornhuber M. A study on viral CNS inflammation beyond herpes encephalitis. J Neurovirol. 2016 Dec;22(6):763–73.

3. Pleumpanupat P, Danchaivijitr N, Danchaivijitr C. Retrospective study of patients with herpes simplex encephalitis and positive CSF PCR. J Med Assoc Thail Chotmaihet Thangphaet. 2009 Mar;92 Suppl 2:S95-100.

4. Granerod J, Ambrose HE, Davies NW, Clewley JP, Walsh AL, Morgan D, et al. Causes of encephalitis and differences in their clinical presentations in England: a multicentre, population-based prospective study. Lancet Infect Dis. 2010 Dec;10(12):835–44.

5. Çiftçi Kavaklioğlu B, Çoban E, Şen A, Söylemezoğlu E, Aldan MA, Atakli D, et al. Review of Viral Encephalitis Cases Seen at a Tertiary Care Center in Turkey: Focus on Herpes Simplex Type 1. Noro Psikiyatri Arsivi. 2017 Sep;54(3):209–15.

6. Misra UK, Kalita J. Seizures in encephalitis: predictors and outcome. Seizure. 2009 Oct;18(8):583–7.

7. Hansen MA, Samannodi MS, Castelblanco RL, Hasbun R. Clinical Epidemiology, Risk Factors, and Outcomes of Encephalitis in Older Adults. Clin Infect Dis Off Publ Infect Dis Soc Am. 2020 May 23;70(11):2377–85.

8. Michael BD, Sidhu M, Stoeter D, Roberts M, Beeching NJ, Bonington A, et al. Acute central nervous system infections in adults--a retrospective cohort study in the NHS North West region. QJM Mon J Assoc Physicians. 2010 Oct;103(10):749–58.

9. Salazar L, Hasbun R. Cranial Imaging Before Lumbar Puncture in Adults With Community-Acquired Meningitis: Clinical Utility and Adherence to the Infectious Diseases Society of America Guidelines. Clin Infect Dis Off Publ Infect Dis Soc Am. 2017 Jun 15;64(12):1657–62.

10. Glimåker M, Johansson B, Bell M, Ericsson M, Bläckberg J, Brink M, et al. Early lumbar puncture in adult bacterial meningitis--rationale for revised guidelines. Scand J Infect Dis. 2013 Sep;45(9):657–63.

11. Gaieski DF, Nathan BR, Weingart SD, Smith WS. Emergency neurologic life support: meningitis and encephalitis. Neurocrit Care. 2012 Sep;17 Suppl 1:S66-72.

12. Bell DJ, Suckling R, Rothburn MM, Blanchard T, Stoeter D, Michael B, et al. Management of suspected herpes simplex virus encephalitis in adults in a U.K. teaching hospital. Clin Med Lond Engl. 2009 Jun;9(3):231–5.

13. Michael BD, Powell G, Curtis S, Bailey L, Almond S, McGill F, et al. Improving the diagnosis of central nervous system infections in adults through introduction of a simple lumbar puncture pack. Emerg Med J EMJ. 2013 May;30(5):402–5.

14. Ellul M, Solomon T. Acute encephalitis - diagnosis and management. Clin Med Lond Engl. 2018 Mar;18(2):155–9.

15. Solomon T, Michael BD, Smith PE, Sanderson F, Davies NWS, Hart IJ, et al. Management of suspected viral encephalitis in adults--Association of British Neurologists and British Infection Association National Guidelines. J Infect. 2012 Apr;64(4):347–73.

16. Tunkel AR, Glaser CA, Bloch KC, Sejvar JJ, Marra CM, Roos KL, et al. The management of encephalitis: clinical practice guidelines by the Infectious Diseases Society of America. Clin Infect Dis Off Publ Infect Dis Soc Am. 2008 Aug 1;47(3):303–27.

17. Tunkel AR, Hartman BJ, Kaplan SL, Kaufman BA, Roos KL, Scheld WM, et al. Practice guidelines for the management of bacterial meningitis. Clin Infect Dis Off Publ Infect Dis Soc Am. 2004 Nov 1;39(9):1267–84.

18. McGill F, Heyderman RS, Michael BD, Defres S, Beeching NJ, Borrow R, et al. The UK joint specialist societies guideline on the diagnosis and management of acute meningitis and meningococcal sepsis in immunocompetent adults. J Infect. 2016 Apr;72(4):405–38.

19. van de Beek D, Cabellos C, Dzupova O, Esposito S, Klein M, Kloek AT, et al. ESCMID guideline: diagnosis and treatment of acute bacterial meningitis. Clin Microbiol Infect Off Publ Eur Soc Clin Microbiol Infect Dis. 2016 May;22 Suppl 3:S37-62.

20. Aurelius E, Johansson B, Sköldenberg B, Forsgren M. Encephalitis in immunocompetent patients due to herpes simplex virus type 1 or 2 as determined by type-specific polymerase chain reaction and antibody assays of cerebrospinal fluid. J Med Virol. 1993 Mar;39(3):179–86.

21. Studahl M, Bergström T, Hagberg L. Acute viral encephalitis in adults--a prospective study. Scand J Infect Dis. 1998;30(3):215–20.

22. Jeren T, Beus I. Characteristics of cerebrospinal fluid in tuberculous meningitis. Acta Cytol. 1982 Oct;26(5):678–80.

23. Broadhurst MJ, Dujari S, Budvytiene I, Pinsky BA, Gold CA, Banaei N. Utilization, Yield, and Accuracy of the FilmArray Meningitis/Encephalitis Panel with Diagnostic Stewardship and Testing Algorithm. J Clin Microbiol. 2020 Aug 24;58(9).

24. Govekar SR, Lakshman LP, Reddy V, Mani RS, Mahadevan A, Susarla SK, et al. Syndrome Evaluation System for Simultaneous Detection of Pathogens Causing Acute Encephalitic Syndrome in India, Part-2: Validation Using Well Characterized Clinical Samples. Front Neurol. 2018;9:1193.

25. Trujillo-Gómez J, Tsokani S, Arango-Ferreira C, Atehortúa-Muñoz S, Jimenez-Villegas MJ, Serrano-Tabares C, et al. Biofire FilmArray Meningitis/Encephalitis panel for the aetiological diagnosis of central nervous system infections: A systematic review and diagnostic test accuracy meta-analysis. EClinicalMedicine. 2022 Feb;44:101275.

26. Gregoire SM, van Pesch V, Goffette S, Peeters A, Sindic CJM. Polymerase chain reaction analysis and oligoclonal antibody in the cerebrospinal fluid from 34 patients with varicella-zoster virus infection of the nervous system. J Neurol Neurosurg Psychiatry. 2006 Aug;77(8):938–42.

27. Ambrose HE, Granerod J, Clewley JP, Davies NWS, Keir G, Cunningham R, et al. Diagnostic strategy used to establish etiologies of encephalitis in a prospective cohort of patients in England. J Clin Microbiol. 2011 Oct;49(10):3576–83.

28. Reusken C, Boonstra M, Rugebregt S, Scherbeijn S, Chandler F, Avšič-Županc T, et al. An evaluation of serological methods to diagnose tick-borne encephalitis from serum and cerebrospinal fluid. J Clin Virol Off Publ Pan Am Soc Clin Virol. 2019 Nov;120:78–83.

29. Forsgren M, Skoldenberg B, Jeansson S, Grandien M, Blomberg J, Juto P, et al. Serodiagnosis of herpes encephalitis by indirect enzyme-linked immunosorbent assay, experience from a Swedish antiviral trial. Serodiagn Immunother Infect Dis. 1989;3:259–71.

30. Sköldenberg B, Forsgren M, Alestig K, Bergström T, Burman L, Dahlqvist E, et al. Acyclovir versus vidarabine in herpes simplex encephalitis. Randomised multicentre study in consecutive Swedish patients. Lancet Lond Engl. 1984 Sep 29;2(8405):707–11.

31. Vandvik B, Sköldenberg B, Forsgren M, Stiernstedt G, Jeansson S, Norrby E. Long-term persistence of intrathecal virus-specific antibody responses after herpes simplex virus encephalitis. J Neurol. 1985;231(6):307–12.

32. Winchester SA, Brown KE. A woman with suspected subacute sclerosing panencephalitis (SSPE). J Clin Virol Off Publ Pan Am Soc Clin Virol. 2011 Feb;50(2):93–5.

33. Solomon T. Flavivirus encephalitis. N Engl J Med. 2004 Jul 22;351(4):370–8.

34. Tan IL, Mowry EM, Steele SU, Pardo CA, McArthur JC, Nath A, et al. Brainstem encephalitis: etiologies, treatment, and predictors of outcome. J Neurol. 2013 Sep;260(9):2312–9.

35. Abdullah KG, Li Y, Agarwal P, Nayak NR, Thawani JP, Balu R, et al. Long-term utility and complication profile of open craniotomy for biopsy in patients with idiopathic encephalitis. J Clin Neurosci Off J Neurosurg Soc Australas. 2017 Mar;37:69–72.

36. Misra UK, Kalita J, Phadke RV, Wadwekar V, Boruah DK, Srivastava A, et al. Usefulness of various MRI sequences in the diagnosis of viral encephalitis. Acta Trop. 2010 Dec;116(3):206–11.

37. Granerod J, Davies NWS, Mukonoweshuro W, Mehta A, Das K, Lim M, et al. Neuroimaging in encephalitis: analysis of imaging findings and interobserver agreement. Clin Radiol. 2016 Oct;71(10):1050–8.

38. Le Maréchal M, Mailles A, Seigneurin A, Tattevin P, Stahl JP, Épaulard O. A Prospective Cohort Study to Identify Clinical, Biological, and Imaging Features That Predict the Etiology of Acute Encephalitis. Clin Infect Dis Off Publ Infect Dis Soc Am. 2021 Jul 15;73(2):264–70.

39. Vitali P, Savoldi F, Segati F, Melazzini L, Zanardo M, Fedeli MP, et al. MRI versus CT in the detection of brain lesions in patients with infective endocarditis before or after cardiac surgery. Neuroradiology. 2022 May;64(5):905–13.

40. Chandra SR, Seshadri R, Chikabasaviah Y, Issac TG. Progressive limbic encephalopathy: Problems and prospects. Ann Indian Acad Neurol. 2014 Apr;17(2):166–70.

41. Mitchell JW, Valdoleiros SR, Jefferson S, Hywel B, Solomon T, Marson AG, et al. Autoimmune encephalitis as an increasingly recognised cause of non-convulsive status epilepticus: A

retrospective, multicentre evaluation of patient characteristics and electroencephalography (EEG) results. Seizure. 2020 Aug;80:153–6.

42. Hjalmarsson A, Granath F, Forsgren M, Brytting M, Blomqvist P, Sköldenberg B. Prognostic value of intrathecal antibody production and DNA viral load in cerebrospinal fluid of patients with herpes simplex encephalitis. J Neurol. 2009 Aug;256(8):1243–51.

43. Mailles A, Stahl JP. Infectious encephalitis in france in 2007: a national prospective study. Clin Infect Dis Off Publ Infect Dis Soc Am. 2009 Dec 15;49(12):1838–47.

44. Rohani H, Arjmand R, Mozhgani SH, Shafiee A, Amini MJ, Forghani-Ramandi MM. The Worldwide Prevalence of Herpes Simplex Virus Encephalitis and Meningitis: A Systematic Review and Meta-Analysis. Turk Arch Pediatr. 2023 Nov;58(6):580–7.

45. de Ory F, Avellón A, Echevarría JE, Sánchez-Seco MP, Trallero G, Cabrerizo M, et al. Viral infections of the central nervous system in Spain: a prospective study. J Med Virol. 2013 Mar;85(3):554–62.

46. de Montmollin E, Dupuis C, Jaquet P, Sarton B, Sazio C, Susset V, et al. Herpes Simplex Virus Encephalitis With Initial Negative Polymerase Chain Reaction in the Cerebrospinal Fluid: Prevalence, Associated Factors, and Clinical Impact. Crit Care Med. 2022 Jul 1;50(7):e643–8.

47. Saraya AW, Wacharapluesadee S, Petcharat S, Sittidetboripat N, Ghai S, Wilde H, et al. Normocellular CSF in herpes simplex encephalitis. BMC Res Notes. 2016 Feb 15;9:95.

48. Sarton B, Jaquet P, Belkacemi D, de Montmollin E, Bonneville F, Sazio C, et al. Assessment of Magnetic Resonance Imaging Changes and Functional Outcomes Among Adults With Severe Herpes Simplex Encephalitis. JAMA Netw Open. 2021 Jul 1;4(7):e2114328.

49. Stahl JP, Mailles A, De Broucker T. Herpes simplex encephalitis and management of acyclovir in encephalitis patients in France. Epidemiol Infect. 2012 Feb;140(2):372–81.

50. Bloch KC, Glaser C, Gaston D, Venkatesan A. State of the Art: Acute Encephalitis. Clin Infect Dis Off Publ Infect Dis Soc Am. 2023 Sep 11;77(5):e14–33.

51. Stahl JP, Azouvi P, Bruneel F, De Broucker T, Duval X, Fantin B, et al. Guidelines on the management of infectious encephalitis in adults. Med Mal Infect. 2017 May;47(3):179–94.

52. Whitley RJ, Alford CA, Hirsch MS, Schooley RT, Luby JP, Aoki FY, et al. Vidarabine versus acyclovir therapy in herpes simplex encephalitis. N Engl J Med. 1986 Jan 16;314(3):144–9.

53. VanLandingham KE, Marsteller HB, Ross GW, Hayden FG. Relapse of herpes simplex encephalitis after conventional acyclovir therapy. JAMA. 1988 Feb 19;259(7):1051–3.

54. Dennett C, Klapper PE, Cleator GM. Polymerase chain reaction in the investigation of 'relapse' following herpes simplex encephalitis. J Med Virol. 1996 Feb;48(2):129–32.

55. Yamada S, Kameyama T, Nagaya S, Hashizume Y, Yoshida M. Relapsing herpes simplex encephalitis: pathological confirmation of viral reactivation. J Neurol Neurosurg Psychiatry. 2003 Feb;74(2):262–4.

56. Schulte EC, Sauerbrei A, Hoffmann D, Zimmer C, Hemmer B, Mühlau M. Acyclovir resistance in herpes simplex encephalitis. Ann Neurol. 2010 Jun;67(6):830–3.

57. Fujii H, Harada S, Yoshikawa T, Yamada S, Omura N, Shibamura M, et al. Differences in the Likelihood of Acyclovir Resistance-Associated Mutations in the Thymidine Kinase Genes of Herpes Simplex Virus 1 and Varicella-Zoster Virus. Antimicrob Agents Chemother. 2019 May;63(5).

58. Schalkwijk HH, Snoeck R, Andrei G. Acyclovir resistance in herpes simplex viruses: Prevalence and therapeutic alternatives. Biochem Pharmacol. 2022 Dec;206:115322.

59. Kuo JY, Yeh CS, Wang SM, Chen SH, Wang JR, Chen TY, et al. Acyclovir-resistant HSV-1 isolates among immunocompromised patients in southern Taiwan: Low prevalence and novel mutations. J Med Virol. 2023 Aug;95(8):e28985.

60. Meyding-Lamadé U, Jacobi C, Martinez-Torres F, Lenhard T, Kress B, Kieser M, et al. The German trial on Aciclovir and Corticosteroids in Herpes-simplex-virus-Encephalitis (GACHE): a multicenter, randomized, double-blind, placebo-controlled trial. Neurol Res Pract. 2019;1:26.

61. Ramos-Estebanez C, Lizarraga KJ, Merenda A. A systematic review on the role of adjunctive corticosteroids in herpes simplex virus encephalitis: is timing critical for safety and efficacy? Antivir Ther. 2014;19(2):133–9.

62. Solomon T, Hooper C, Easton A, Rosala-Hallas A, Facer B, Moore P, et al. Safety and efficacy of adjunct dexamethasone in adults with herpes simplex virus encephalitis in the UK (DexEnceph): a multicentre, observer-blind, randomised, phase 3, controlled trial. Lancet Neurol. 2026 Feb;25(2):136–46.

63. Poussier L, Mailles A, Tattevin P, Stahl JP, Fillâtre P. Characteristics, management and outcome of Herpes Simplex and Varicella-Zoster virus encephalitis: a multicentre prospective cohort study. Clin Microbiol Infect Off Publ Eur Soc Clin Microbiol Infect Dis. 2024 Jul;30(7):917–23.

64. Collins P. The spectrum of antiviral activities of acyclovir in vitro and in vivo. J Antimicrob Chemother. 1983 Sep;12 Suppl B:19–27.

65. Martinot M, Klein A, Demesmay K, Groza M, Mohseni-Zadeh M, Tebacher-Alt M, et al. Acute renal failure related to high doses of acyclovir (15 mg/kg/8 h) during treatment of varicella zoster virus encephalitis. Antivir Ther. 2019;24(4):309–12.

66. Lee EJ, Jang HN, Cho HS, Bae E, Lee TW, Chang SH, et al. The incidence, risk factors, and clinical outcomes of acute kidney injury (staged using the RIFLE classification) associated with intravenous acyclovir administration. Ren Fail. 2018 Nov;40(1):687–92.

67. Herlin LK, Hansen KS, Bodilsen J, Larsen L, Brandt C, Andersen CØ, et al. Varicella Zoster Virus Encephalitis in Denmark From 2015 to 2019-A Nationwide Prospective Cohort Study. Clin Infect Dis Off Publ Infect Dis Soc Am. 2021 Apr 8;72(7):1192–9.

68. Wu H, Wang R, Li Y, Sun X, Li J, Bi X. Cerebrovascular Complications After Adult-Onset Varicella-Zoster Virus Encephalitis in the Central Nervous System: A Literature Review. Neuropsychiatr Dis Treat. 2022;18:449–62.

69. Gaitatzis A, Sander JW. The long-term safety of antiepileptic drugs. CNS Drugs. 2013 Jun;27(6):435–55.

70. Leng X, Yuan F, Zhao J, Song C, Zhao Z, Zhang Y, et al. Long-term seizure outcome in patients with status epilepticus due to acute encephalitis. Seizure. 2019 Jul;69:70–5.

71. Peng A, Lai W, Li W, Qiu X, Zhang L, He S, et al. Antiepileptic drugs for acute encephalitic patients presented with seizure. Epilepsy Res. 2020 Aug;164:106347.

72. Solomon T, Dung NM, Kneen R, Thao LTT, Gainsborough M, Nisalak A, et al. Seizures and raised intracranial pressure in Vietnamese patients with Japanese encephalitis. Brain J Neurol. 2002 May;125(Pt 5):1084–93.

73. Alam AM, Chen JPK, Wood GK, Facer B, Bhojak M, Das K, et al. Correction: Increased volume of cerebral oedema is associated with risk of acute seizure activity and adverse neurological outcomes in encephalitis - regional and volumetric analysis in a multi-centre cohort. BMC Neurol. 2022 Dec 5;22(1):451.

74. Sellner J, Trinka E. Seizures and epilepsy in herpes simplex virus encephalitis: current concepts and future directions of pathogenesis and management. J Neurol. 2012 Oct;259(10):2019–30.

75. Misra UK, Tan CT, Kalita J. Viral encephalitis and epilepsy. Epilepsia. 2008 Aug;49 Suppl 6:13–8.

76. Hjalmarsson A, Blomqvist P, Sköldenberg B. Herpes simplex encephalitis in Sweden, 1990-2001: incidence, morbidity, and mortality. Clin Infect Dis Off Publ Infect Dis Soc Am. 2007 Oct 1;45(7):875–80.

77. Pandey S, Rathore C, Michael BD. Antiepileptic drugs for the primary and secondary prevention of seizures in viral encephalitis. Cochrane Database Syst Rev. 2016 May 22;2016(5):CD010247.

78. Singh TD, Fugate JE, Hocker S, Wijdicks EFM, Aksamit AJJ, Rabinstein AA. Predictors of outcome in HSV encephalitis. J Neurol. 2016 Feb;263(2):277–89.

79. Chalfin DB, Trzeciak S, Likourezos A, Baumann BM, Dellinger RP. Impact of delayed transfer of critically ill patients from the emergency department to the intensive care unit. Crit Care Med. 2007 Jun;35(6):1477–83.

80. Rincon F, Mayer SA, Rivolta J, Stillman J, Boden-Albala B, Elkind MSV, et al. Impact of delayed transfer of critically ill stroke patients from the Emergency Department to the Neuro-ICU. Neurocrit Care. 2010 Aug;13(1):75–81.

81. Helbok R, Olson DM, Le Roux PD, Vespa P. Intracranial pressure and cerebral perfusion pressure monitoring in non-TBI patients: special considerations. Neurocrit Care. 2014 Dec;21 Suppl 2:S85-94.

82. Armangue T, Moris G, Cantarín-Extremera V, Conde CE, Rostasy K, Erro ME, et al. Autoimmune post-herpes simplex encephalitis of adults and teenagers. Neurology. 2015 Nov 17;85(20):1736–43.

83. Armangue T, Spatola M, Vlagea A, Mattozzi S, Cárceles-Cordon M, Martinez-Heras E, et al. Frequency, symptoms, risk factors, and outcomes of autoimmune encephalitis after herpes simplex encephalitis: a prospective observational study and retrospective analysis. Lancet Neurol. 2018 Sep;17(9):760–72.

84. Nosadini M, Mohammad SS, Corazza F, Ruga EM, Kothur K, Perilongo G, et al. Herpes simplex virus-induced anti-N-methyl-d-aspartate receptor encephalitis: a systematic literature review with analysis of 43 cases. Dev Med Child Neurol. 2017 Aug;59(8):796–805.

85. Christie S, Chan V, Mollayeva T, Colantonio A. Systematic review of rehabilitation intervention outcomes of adult and paediatric patients with infectious encephalitis. BMJ Open. 2018 May 14;8(5):e015928.

86. Cooper J, Kierans C, Defres S, Easton A, Kneen R, Solomon T. Care beyond the hospital ward: understanding the socio-medical trajectory of herpes simplex virus encephalitis. BMC Health Serv Res. 2017 Sep 12;17(1):646.
